# Phytochemical Screening and Antioxidant Potential of Selected Extracts from *Betula alba* var. *pendula* Roth., *Glycyrrhiza glabra* L., and *Avena sativa* L.

**DOI:** 10.3390/plants12132510

**Published:** 2023-06-30

**Authors:** Adelina Ghica, Veronica Drumea, Alina Moroșan, Dan Eduard Mihaiescu, Liliana Costea, Emanuela Alice Luță, Dragos Paul Mihai, Dalila Teodora Balaci, Ancuța Cătălina Fița, Octavian Tudorel Olaru, Rica Boscencu, Cerasela Elena Gîrd

**Affiliations:** 1 Faculty of Pharmacy, “Carol Davila” University of Medicine and Pharmacy, Traian Vuia 6, 020956 Bucharest, Romania; emanuela.luta@drd.umfcd.ro (E.A.L.); teodora.balaci@umfcd.ro (D.T.B.); catalina.fita@umfcd.ro (A.C.F.); octavian.olaru@umfcd.ro (O.T.O.); rica.boscencu@umfcd.ro (R.B.); cerasela.gird@umfcd.ro (C.E.G.); 2 Biotehnos SA, Gorunului Street No. 3-5, 075100 Otopeni, Romania; veronica.drumea@biotehnos.com; 3 Department of Organic Chemistry “Costin Nenițescu”, Faculty of Applied Chemistry and Materials Science, University POLITEHNICA of Bucharest, 011061 Bucharest, Romania; dan.mihaiescu@upb.ro

**Keywords:** phytochemical screening, radical scavenging activity, FT-ICR-MS, molecular formula, molecular docking, Keap1-Nrf2 pathway, skin disorders

## Abstract

The aim of the present study was to obtain, characterize, and evaluate the antioxidant potential of some extracts obtained from the bark of *Betula alba* var. *pendula* Roth., the root of *Glycyrrhiza glabra* L., and the green herb of the *Avena sativa*. The results revealed that the lowest IC50 value, determined by all three methods, was obtained for *Betulae extractum* (BE) (73.6 µg/mL—DPPH method, 11.2 µg/mL—ABTS method, and 58.7 µg/mL—FRAP method), followed by *Liquiritiae extractum* (LE) (805.6 µg/mL, 92.1 µg/mL, and 722 µg/mL) and *Avenae extractum* (1.13 mg/mL—DPPH method, 99.7 µg/mL—ABTS method, and 135.1 µg/mL—FRAP method). These results correlate with total polyphenols content (expressed in g tannic acid/100 g dry extract), with BE having more polyphenols than LE and AE (47.96 ± 9.7083 for BE, compared with 9.31 ± 0.9913 for LE and 40.55 ± 6.3715 for AE). The total flavonoid content (expressed as g rutoside/100 g dry extract) is similar for BE and LE (3.75 ± 0.3140 and 3.44 ± 0.3037) and smaller for AE (1.95 ± 0.0526). Therefore, *Betulae extractum* has the strongest antioxidant action, with an IC50 value very close to the standard used as a reference (ascorbic acid—16.5 μg/mL solution). The FT-ICR-MS analysis confirmed the presence of the major compounds in all three extracts. The antioxidant properties of the studied extracts were further supported by molecular docking experiments that revealed the potential of the analyzed phytochemicals to act as both noncovalent and covalent activators of the Nrf2 signaling pathway, with promising benefits in treating various skin disorders.

## 1. Introduction

Radical scavenging activity is very important in preventing cell damage by free radicals, including well known reactive oxygen species (ROS). Antioxidants have the role of counteracting the negative impact of free radicals and thus preventing the development of excess oxidative stress-induced illnesses [1]. The plants are rich in substances with antiradical activity, such as flavonoids and phenolic compounds, so the extracts could be an alternative therapy for various diseases [2,3].

The *Betulaceae* family is an important category of species that exhibit various pharmacological and therapeutic properties. *Betulae* species, part of the *Betulaceae* group, are known for their high contents of pentacyclic triterpenes, flavones, tannins, glycosides, saponins, coumarins, essential oils, vitamins, sterols, etc., distributed in various parts of the plant and which provide pharmacological activities, such as antimicrobial, anti-inflammatory, immunological, antiviral, antifungal, analgesic, etc. Traditionally, birch extracts were used in combination to relieve arthritis, fever, gout, headaches, kidney stones, and rheumatism [4,5]. The composition and pharmacological potential of some *Betula* species present great interest, especially for their wound healing and anti-inflammatory properties. Antioxidant potential was studied the most for birch leaf extracts based on phytochemical composition, and it was studied less for the cortex extracts [5,6,7,8,9].

Well known for its radical scavenging potential is *Glycyrrhiza glabra* L. root extract, mainly through its compound glycyrrhetinic acid [10,11]. Licorice radix has a complex composition, whose predominant compounds belong to the classes of flavonoids (52 compounds) and triterpenoids (47 compounds) [12]. Many studies have highlighted the important contribution of flavonoids (flavones such as rutin, luteolin, apigenin, isoflavones, such as glabridin, and phenol chalconoids, such as licochalcones B and D) in the development of antiradical action, which was found to be over 100-fold stronger towards the antiradical activity of vitamin E [13,14,15,16]. Additionally, it has a very good anti-inflammatory effect and releases pain, activities demonstrated by the liquiritigenin inhibition properties on the TMEM16A channel (transmembrane channel) [17]. Additionally, 18β-glycyrrhetinic acid anti-inflammatory and pro-apoptotic effects in in vitro and in vivo models of rheumatoid models were observed [18]. Finally, it even acts as an immunostimulatory agent, as seen in a study on rainbow trout fingerlings [19].

The *Avena sativa* L. green herb, harvested before flowering, is well known for its neurological activities, stimulation of dopaminergic neurotransmission implicated in cognitive functioning, motivation, and depression [20]. Traditionally, oat is considered healthy because it contains proteins and lipids, essential amino acids, such as lysine, polyphenols, including caffeic acids, coumaric acids, gallic acids, hydroxybenzoic acids, saponins, and 2–6% of β-glucan. Additionally, oats have 5–12% fat, with approximately 95% being palmitic acid, oleic acid, linoleic acid, and linolenic acid, which are associated with the prevention of dementia and antioxidant activity. β-Glucan also has an important role in wound healing and antioxidant activities [21]. The colloidal oatmeal (oat grains ground to a fine powder) approved by FDA is an ingredient in over-the-counter products and also a traditional herbal medicinal product authorized by EMA, with beneficial effects on minor skin irritation and itching due to rashes, eczema, and dry and itchy skin [22]. However, for the green herb extract, few studies are available regarding its applicability [21]. 

Molecular docking studies allow one to obtain information from a molecular perspective, predicting the binding conformation of the identified phytochemicals from the extracts to specific molecular targets with an impact on exerting in vivo antioxidant activity.

Nuclear factor erythroid 2-related factor 2 (Nrf2, nuclear factor erythroid-derived 2-like 2) is a transcription factor that binds to antioxidant response elements (AREs) after nuclear translocation, promotes endogenous antioxidant defense mechanisms, and also inhibits the NLRP3 inflammasome [23]. Nrf2 is inhibited by direct interaction with Keap1 (Kelch-like ECH-associated protein 1), which regulates the nuclear factor’s activity by facilitating its ubiquitination and subsequent proteasome-dependent degradation [24]. The Keap1 structure is characterized by three domains with key roles in its function: the N-terminal BTB domain, the central intervening region (IVR), and the C-terminal Kelch domain. The BTB domain is necessary for homodimerization and, together with the IVR domain, interacts with Cul3 (Cullin 3), implicated in Nrf2 ubiquitination [25]. The Kelch domain, after Keap1 dimerization, binds to either ETGE or DLG motifs of Nrf2, capturing the nuclear factor into the cytoplasm [26]. Keap1–Nrf2 interaction and ubiquitination of Nrf2 can be hindered either by blocking the interaction site between the two proteins (by noncovalent binding at the protein–protein binding site or covalent binding to Cys434 in the Kelch domain) or by preventing the interaction between the BTB domain and Cul3 (through covalent modifications of key reactive cysteine residues, such as Cys77, Cys151, and Cys171), thus leading to nuclear translocation and antioxidant, anti-inflammatory activities [25,27,28,29]. Among many oxidative stress-related and inflammatory disorders, the Nrf2 signaling pathway could be pharmacologically targeted to also alleviate skin conditions, such as psoriasis, vitiligo, melanoma, and possibly atopic dermatitis and UV-induced damage [30,31,32,33]. Interestingly, various natural compounds were uncovered as Keap1 inhibitors and Nrf2 activators, such as curcumin, xanthohumol, 10-shogaol, and sulforaphane [34,35,36]. 

Considering all the above, the present study aims to compare the radical scavenging activity of the *Betulae cortex*, *Liquiritiae radix*, and *Avenae herba* ethanolic extracts and to characterize them through a phytochemical screening, with the purpose of including the extracts in future topical products for improving various skin illnesses. Therefore, in this present study, we also aimed to investigate the potential of the analyzed phytochemicals to bind and inhibit Keap1, both covalently and noncovalently, thus leading to Nrf2 nuclear translocation and beneficial antioxidant and anti-inflammatory effects in various skin disorders.

## 2. Results

### 2.1. The Extraction Process Yields

The values of the extraction yields, calculated by subtracting the weight of the dry extract from the initial bark, root, or herb powder weight, were 4% (BE) and 20% (LE and AE).

### 2.2. The Determination of Total Flavonoid Content (TFC), Total Polyphenol Content (TPC), and Total Phenolic Acid Content (TPA) Using Spectrophotometric Methods

The results obtained from the determinations are presented in Table 1.

**Figure 1:**
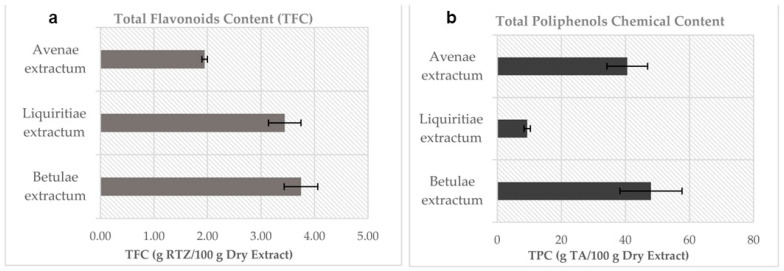
Chemical content in plant dry extracts: (**a**). TFC by CH_3_COONa-AlCl_3_ assay and (**b**). TPC by the Folin-Ciocalteu assay. (n = 5; error bars represent standard deviation).

The results showed that BE has the highest content in TFC and TPC among all studied extracts. LE has almost twice as many flavonoids as AE, whereas the TPC of AE is four-fold higher than LE (Figure 1). Phenolic acids (TPA) could be determined only for the BE extract. In the case of AE, the phenolic acids are concentrated in the outer bran layers [37], which explains the lack of them in the ethanolic extract. For LE, the variability in phenolic constituents and the variability in phenolic content are due to the plant used, environmental factors, and the collection period [38]. 

The standard curves of rutoside for the total flavonoid content assay (TFC), of tannic acid for the total polyphenols content assay (TPC), and of chlorogenic acid for the total phenolic acid content assay (TPA) can be found in Supplementary Material Figures S50–S52. 

### 2.3. GC-MS Analysis

GC-MS chromatography was used to reveal the main phytochemical compounds of the extracts and to identify the fatty acids and the presence of volatile substances. 

**Figure 2:**
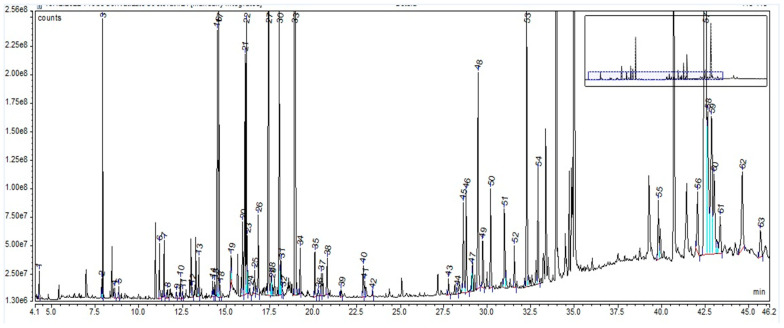
GC-MS chromatogram of *Betulae extractum*: peak 1. glycolic acid, 2TMS; peak 2. phosphoric acid, 3TMS; peak 3. glycerol, 3TMS; peak 4. succinic acid, 2TMS; peak 5. glyceric acid, 3TMS; peak 6. malic acid, 3TMS; peak 7. erythritol, TMS; peak 8. L-5-oxoproline, 2TMS; peak 9. erythronic acid, 3TMS; peak 10. 4-hydroxyphenylethanol, 2TMS (tyrosol); peak 11. L-(+)-tartaric acid, 4TMS; peak 12. 3-hydroxybenzoic acid, 2TMS; peak 13. arabinose, 4TMS; peak 14. ribitol, 5TMS; peak 15. L-(−)-arabitol, 5TMS; peak 16. phenolic derivative; peak 17. xylitol, 5TMS derivative; peak 18. adonitol, 5TMS; peak 19. hydrocinnamic acid, 3TMS; peak 20. D-(−)-fructofuranose, 5TMS (isomer 1); peak 21. D-(−)-fructofuranose, 5TMS (isomer 2); peak 22. D-(−)-fructopyranose, 5TMS; peak 23. D-protocatechoic acid, 3TMS; peak 24. D-pinitol; peak 25. D-allofuranose, 5TMS; peak 26. quininic acid, 5TMS; peak 27. D-mannose, 5TMS; peak 28. talose, 5TMS; peak 29. D-altrose, 5TMS; peak 30. D-mannitol, 6TMS; peak 31. D-sorbitol, 6TMS; peak 32. D-glucitol, 6TMS; peak 33. D-glucopyranose, 5TMS; peak 34. D-gluconic acid, 6TMS; peak 35. palmitic acid, TMS; peak 36. catechollactate, 3TMS; peak 37. 4-coumaric acid, 2TMS; peak 38. myo-inositol, 6TMS; peak 39. caffeic acid, 3TMS; peak 40. linoleic acid, TMS; peak 41. oleic acid, TMS; peak 42. stearic acid, TMS; peak 43. D-(+)-turanose, 8TMS; peak 44. 2-palmitoylglycerol, 2TMS; peak 45. 2-a-mannobiose, 8TMS (isomer 1); peak 46. 1-monopalmitin, 2TMS; peak 47. 2-a-mannobiose, 8TMS (isomer 2); peak 48. sucrose, 8TMS; peak 49. 2-a-mannobiose, 8TMS (isomer 2); peak 50. galactopyranose, 5TMS; peak 51. D-(+)-trehalose, 8TMS ether; peak 52. glycerol monostearate, 2TMS; peak 53. ß-Lactose, 8TMS; peak 54. catechine (2R-E), 5TMS derivative; peak 55. lupeolic derivative 1, 3TMS; peak 56. betulinic derivative, TMS; peak 57. lupeol, TMS; peak 58. betulin; peak 59. betulinaldehide; peak 60. lupeolic derivative 2, 3TMS; peak 61. lupeolic derivative 3, 3TMS; peak 62. ursolic acid, 2TMS; peak 63. lupeolic derivative 4, 3TMS. The vertical axis notation“e6”, “e7” and “e8” corresponds to “×10^6^”, “×10^7^”, “×10^8^”.

The GC-MS chromatogram (Figure 2), resulting from the derivatization method applied to all three extracts, revealed, for the BE, a total of 63 phytochemical compounds (as trimethylsilyl (TMS) derivatives) grouped in nine classes (Supplementary Material Table S2), of which the main components are 3-epi-betulinaldehide (40.69%), D-glucopyranose (12.30%), D-mannose (4.16%), betulinaldehide (3.35%), and betulin (3.32%). The compounds, with their retention time and percentage area, are listed in Supplementary Materials Table S3.

**Figure 3:**
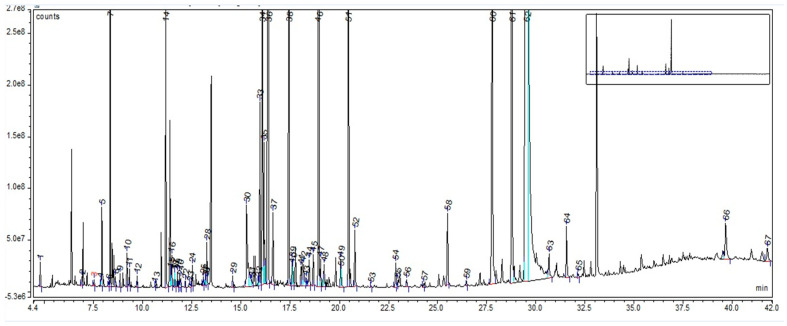
GC-MS chromatogram of *Liquiritiae extractum*: peak 1. L-alanine, 2TMS; peak 2. L-valine, 2TMS; peak 3. benzoic acid, TBDMS; peak 4. phosphoric acid, TMS; peak 5. glycerol, 3TMS; peak 6. L-leucine, 2TMS; peak 7. L-proline, 2TMS; peak 8. succinic acid, 2TMS; peak 9. glyceric acid, 3TMS; peak 10. fumaric acid, di-TMS; peak 11. serine, 3TMS; peak 12. L-threonine, 3TMS; peak 13. homoserine, 3TMS; peak 14. malic acid, 3TMS; peak 15. meso-erythritol, 4TMS; peak 16. pipecolic acid, 2TMS; peak 17. L-aspartic acid, 3TMS; peak 18. 5-oxoproline, 2TMS; peak 19. 4-aminobutanoic acid, 3TMS; peak 20. 1-deoxypentitol, 4TMS; peak 21. erythronic acid, 4TMS; peak 22. L-threonic acid, 3TMS; peak 23. hydroxyglutaric acid, 3TMS; peak 24. L-valine, 2TMS; peak 25. L-glutamic acid, 3TMS; peak 26. phenylalanine, 2TMS; peak 27. 4-hydroxybenzoic acid, 2TMSl peak 28. arabinoic acid, 3TMS; peak 29. xylitol, 5TMS; peak 30. hydrocinnamic acid, 2TMS; peak 31. 2-keto-L-gluconic acid; peak 32. methyl a-D-glucofuranoside, 4TMS; peak 33. D-(−)-fructofuranose, pentakis (trimethylsilyl) ether (isomer 1); peak 34. D-(−)-fructofuranose, pentakis (trimethylsilyl) ether (isomer 2); peak 35. D-(−)-fructopyranose, 5TMS; peak 36. D-pinitol, 5TMS; peak 37. D-allofuranose, 5TMS; peak 38. D-mannose, 5TMS; peak 39. talose, 5TMS; peak 40. D-altrose, 5TMS; peak 41. D-mannitol, 6TMS; peak 42. D-sorbitol, 6TMS; peak 43. hydrocaffeic acid, 3TMS; peak 44. D-pinitol, 5TMS ether; peak 45. D-talofuranose, 5TMS (isomer 1); peak 46. D-glucopyranose, 5TMS; peak 47. D-talofuranose, 5TMS (isomer 2); peak 48. D-gluconic acid, 6TMS; peak 49. cinnamic acid, 3TMS; peak 50. palmitic acid, TMS; peak 51. 4-coumaric acid, 2TMS; peak 52. myo-inositol, 6TMS; peak 53. caffeic acid, 3TMS; peak 54. linoleic acid, TMS; peak 55. oleic acid, TMS; peak 56. stearic acid, TMS; peak 57. D-(+)-Cellobiose, (isomer 1), 8TMS; peak 58. galacturonic acid, 5TMS; peak 59. D-(+)-Cellobiose, (isomer 2), 8TMS; peak 60. D-(+)-Turanose, 8TMS ether; peak 61. D-trehalose, 7TMS; peak 62. sucrose, 8TMS; peak 63. maltose, 8TMS; peak 64. glycerol monostearate, 2TMS; peak 65. equilin, TMS; peak 66. 3-a-mannobiose, 8TMS isomer 1; peak 67. aucubin, 6TMS ether. The vertical axis notation“e6”, “e7” and “e8” corresponds to “×10^6^”, “×10^7^”, “×10^8^”.

In the case of LE, the GC-MS chromatogram highlighted a total of 67 compounds (as trimethylsilyl (TMS) derivatives) grouped in 10 classes (Supplementary Material Table S4). Their retention time and percentage area are listed in Supplementary Materials Table S5. The highest percentages were assigned to sucrose (61.8%), D-pinitol (7.31%), D-glucopyranose (4.13%), L-proline (2.76%), and 4-coumarinic acid (1.56%), as can be seen in Figure 3. 

**Figure 4:**
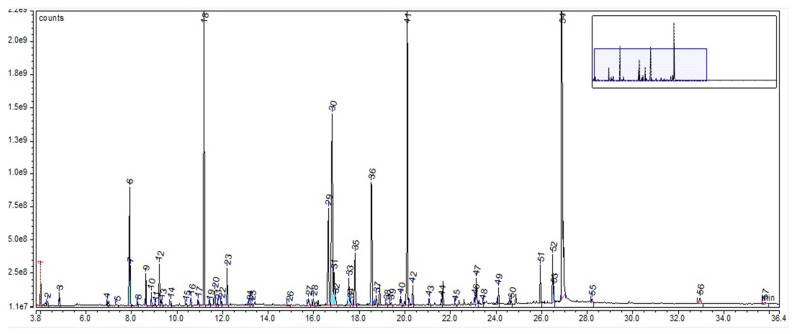
GC-MS chromatogram of *Avenae extractum;* peak 1. lactic acid, 2TMS; peak 2. glycolic acid, 2TMS; peak 3. L-alanine, 2TMS; peak 4. L-valine, 2TMS; peak 5. urea, 2TMS; peak 6. phosphoric acid, TMS; peak 7. glycerol, 3TMS; peak 8. L-isoleucine, 2TMS; peak 9. succinic acid, 2TMS; peak 10. glyceric acid, 3TMS; peak 11. itaconic acid, 2TMS; peak 12. fumaric acid, 2TMS; peak 13. L-serine, 3TMS; peak 14. L-threonine, 3TMS; peak 15. dihidroxibutanoic acid, 3TMS; peak 16. 2-Isopropyl-3-ketobutyrate, 2TMS; peak 17. L-aspartic acid, 3TMS; peak 18. malic acid, 3TMS; peak 19. erythritol, 4TMS; peak 20. 5-oxoproline, 2TMS; peak 21. 4-aminobutanoic acid, 3TMS; peak 22. erythronic acid, 4TMS; peak 23. L-Threonic acid, tris(trimethylsilyl) ether-3TMS; peak 24. L-glutamic acid, 3TMS; peak 25. phenylalanine, 2TMS; peak 26. xylitol, 5TMS; peak 27. 2-Keto-l-gluconic acid, 5TMS; peak 28. ribonic acid, 5TMS; peak 29. D-(−)-fructofuranose, pentakis (trimethylsilyl) ether (isomer 1); peak 30. D-(−)-fructofuranose, pentakis (trimethylsilyl) ether (isomer 2); peak 31. D-psicopyranose, 5TMS; peak 32. citric acid, 4TMS; peak 33. D-(+)-talofuranose, 5TMS; peak 34. D-fructose, 5TMS; peak 35. quininic acid, 5TMS; peak 36. a-D-mannopyranose, 5TMS; peak 37. D-glucose, 5TMS; peak 38. D-mannitol, 6TMS; peak 39. 4-coumaric acid, 2TMS; peak 40. D-allufuranose, 5TMS; peak 41. D-glucopyranose, 5TMS; peak 42. D-gluconic acid, 6TMS; peak 43. palmitic acid, TMS; peak 44. myo-inositol, 6TMS; peak 45. caffeic acid, 3TMS; peak 46. linoleic acid, TMS; peak 47. a-linolenic acid, TMS; peak 48. stearic acid, TMS; peak 49. glyceryl-glycoside, TMS; peak 50. D-(+)-Galacturonic acid, 5TMS; peak 51. sucrose, 8TMS; peak 52. D-(+)-turanose, 8TMS; peak 53. 1-monopalmitin, 2TMS; peak 54. sucrose, 8TMS; peak 55. glycerol monostearate, 2TMS; peak 56. ß-D-lactose, 8TMS; peak 57. D-(+)-trehalose, 8 TMS. The vertical axis notation“e7”, “e8” and “e9” corresponds to “×10^7^”, “×10^8^”, “×10^9^”.

For AE, the GC-MS chromatogram highlighted a total of 57 compounds (as trimethylsilyl (TMS) derivatives) grouped in 10 classes (Supplementary Material Table S6). Their retention time and percentage area are listed in Supplementary Materials Table S6. The highest percentages were assigned to sucrose (28.03%), D-glucopyranose (12.62%), D-(−)-fructofuranose (16.34% isomer 1 + isomer 2), malic acid (9.62%), D-mannopyranose (6.09%), quininic acid (2.73%), lactic acid (1.35%), and linolenic acid (1.11%), as can be seen in Figure 4. The compounds, with their retention time and percentage area, are listed in Supplementary Materials Table S7.

Applying the specific method for identification of the fatty acids, the GC-MS results showed the presence of the following fatty acids (Table 2): 

Based on the calibration curve obtained for linoleic acid and linolenic acid, the quantities found were (Table 3):

The volatile compounds identified through the head space technique were in small number and only in the case of *Betula alba* var. *pendula* Roth. bark ethanolic extract, being represented by sesquitujene, α-santalen, α-bergamoten, α-cucurmen, ß-farnesen, and bisabolen. 

### 2.4. Fourier-Transform Ion Cyclotron Resonance Mass Spectrometer (FT-ICR-MS) Analysis

**Figure 5:**
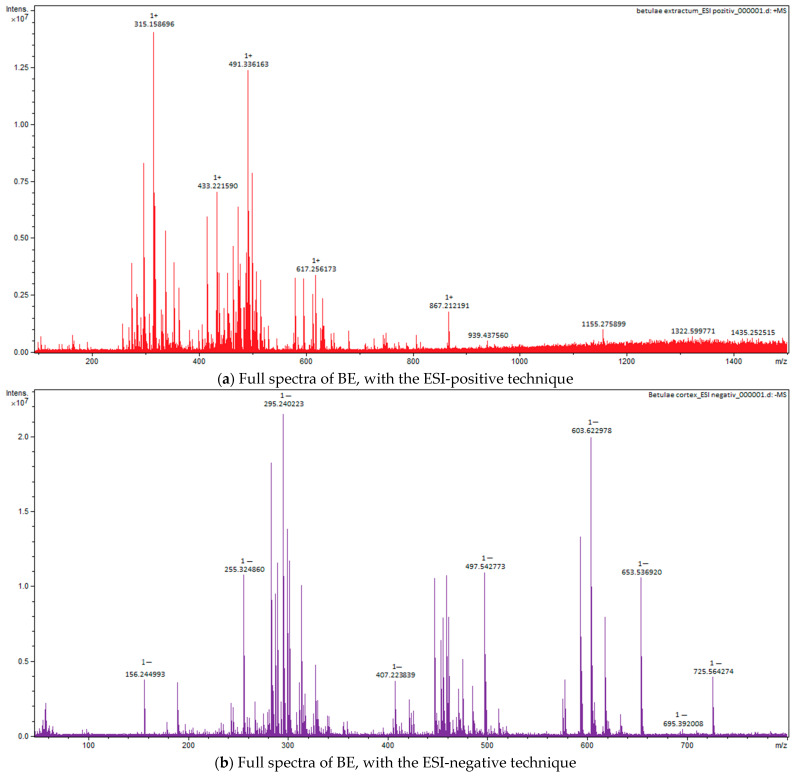
Full spectra of BE, with the ESI-positive (**a**) and negative (**b**) techniques.

**Figure 6:**
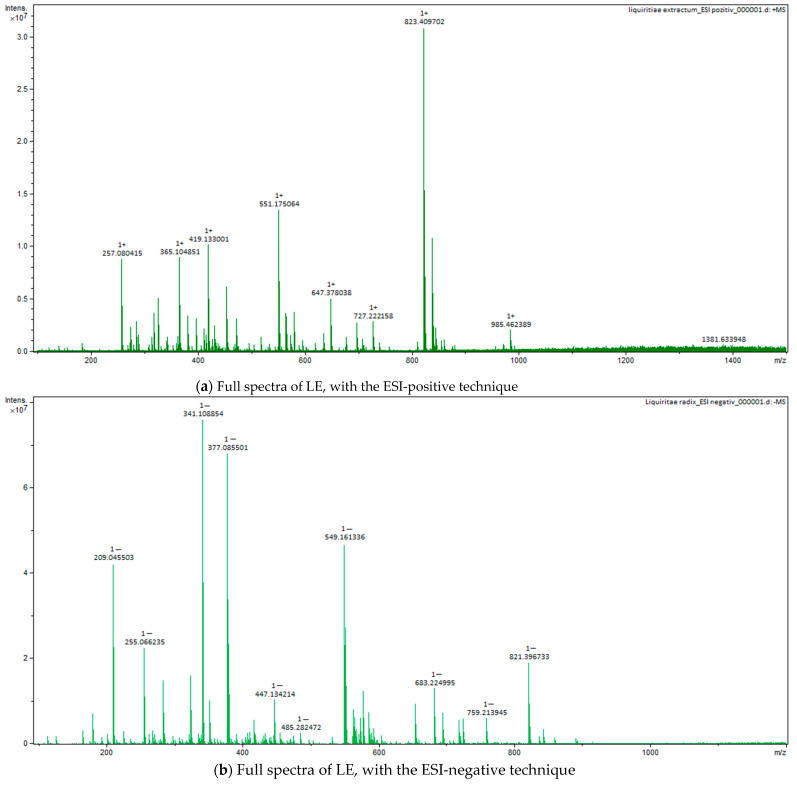
**Figure 6**. Full spectra of LE, with ESI-positive (**a**) and negative (**b**) techniques.

**Figure 7:**
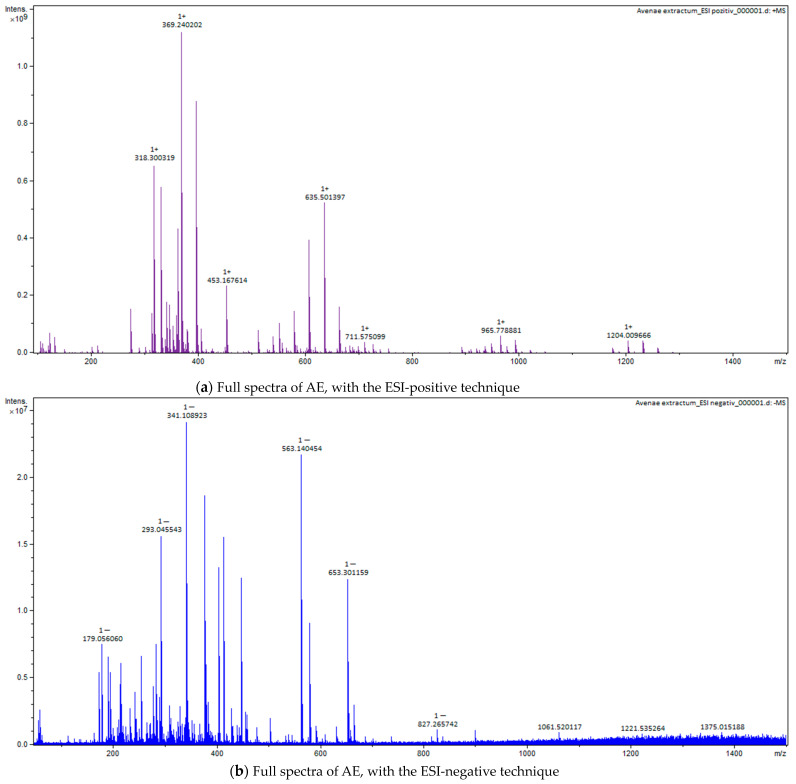
Full spectra of AE, with the ESI-positive (**a**) and negative (**b**) techniques.

For all three extracts, for Fourier Transform Ion Cyclotron Resonance Mass Spectrometer (FT-ICR-MS) analysis, ESI positive and negative techniques were applied, with identification of the important compounds, as can be seen in the full spectra (Figure 5, Figure 6 and Figure 7). The main classes of compounds are polyphenols, triterpenes, flavonoids, and fatty acids (Table 4). FT-ICR-MS spectra can be found in Supplementary Materials Figures S1–S49. Detailed information about some compounds from *Betulae extractum* (betulinic acid, oleanolic acid, ursolic acid, betulin, erythrodiol, betulinaldehide, betulonic acid, lupenone, lupeol, stearic acid, oleic acid, linoleic acid, betuloside, caffeic acid, kaempferol, myo-inositol), from *Liquiritiae extractum* (glycyrrhizin/glycyrrhizinic acid, glycyrrhetinic acid, liquiritigenin/isoliquiritigenin, glabridin, linoleic acid, palmitic acid, myo-inositol, glycyrrhetinic acid), and from *Avenae extractum* (avenacoside A, linolenic acid, oleic acid, palmitic acid, linoleic acid, sucrose, myo-inositol, D-mannose, D-glucopyranose, beta-glucan, kestose/neokestose, tryptophan, tricin, vitexin, caffeic acid, ferulic acid, citric acid), such as measured mass (m/z) and calculated mass (m/z) values can be found in Supplementary Materials Table S1. 

### 2.5. Radical Scavenging Activity

The antioxidant activity of the plant extracts can be assessed through different methods, but it is recommended to use at least two different techniques [39]. For the quantification of BE, LE, and AE antioxidant activity, there were applied three important methods: scavenging activity of 2,2 diphenyl-1-picrylhydrazyl (DPPH) radical, scavenging activity of ABTS radical, and using, as oxidant, ABTS (2,2′-azino-bis-3-ethylbenzthiazoline-6-sulphonic acid) and the Ferric Reducing Antioxidant Power (FRAP) method. The results obtained with these three methods are found in Table 5, highlighting a higher antioxidant activity for BE, with an IC50 value very close to vitamin C, which is used as a reference. The standard curve of ascorbic acid can be found in the Supplementary Materials, Figure S53.

### 2.6. Statistical Analysis

#### 2.6.1. Multiple Box Plots for the Antioxidant Activity

In order to visualize the statistical summary of each antioxidant group, we created several box plots for each set of experimental data allocated to each plant extract analyzed with a certain antioxidant method. Every box plot has the ability to illustrate the distribution of data values within the same experimental group.

Box plots are actually the graphic representation of the minimum value, the maximum value, the first quartile, the median, and the third quartile, which belong to the analyzed data set. Additionally, every outlier that deviates from the normal distribution of the data can be identified by measuring the distance between the third quartile and the first quartile, called the interquartile range (IQR). Any data value can be considered an outlier if it is 1.5 times the IQR larger than the third quartile or 1.5 times the IQR smaller than the first quartile.

Since we have to analyze several antioxidant variables for different plant extract groups, we create multiple side-by-side box plots and then interpret and compare them. Thus, the multiple box plots created help us easily visualize the differences in the distributions between the three studied vegetal extracts.

**Figure 8:**
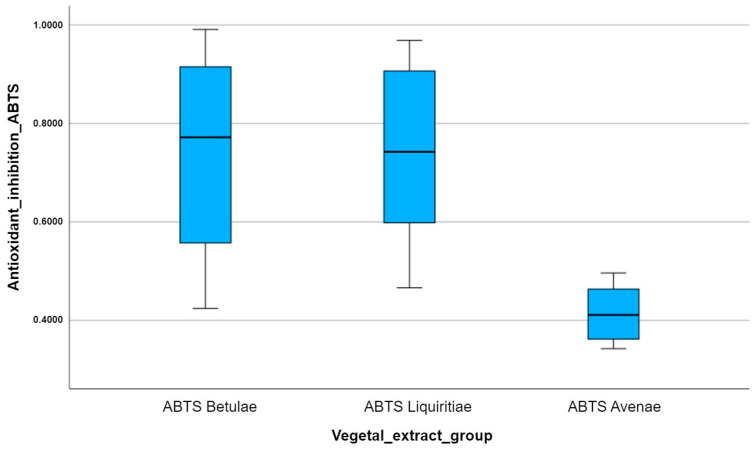
A comparison of the multiple box plots for the ABTS antioxidant method.

After comparing the multiple box plots for the ABTS antioxidant method applied to the three vegetal extracts, we can observe the following: the median inhibition of free radical reactivity ABTS per vegetal extract is highest for *Betulae extractum* (BE) and lowest for *Avenae extractum* (AE); the variation in the number of points scored per plant extract is highest for *Betulae extractum* (BE), which can be seen by how long the box plot is compared to *Liquiritiae extractum* (LE) and *Avenae extractum* (AE) (Figure 8); the vegetal extract with the highest antioxidant activity is *Betulae extractum* (BE), while the vegetal extract with the lowest antioxidant activity is *Avenae extractum* (AE).

**Figure 9:**
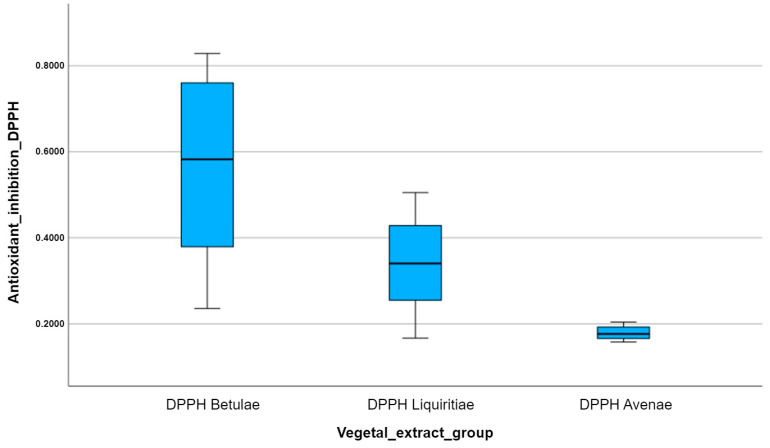
A comparison of the multiple box plots for the DPPH antioxidant method.

Box plots are useful because they can provide us with so much information about the distribution of data sets from a single plot. Therefore, we obtained the same results by applying the DPPH antioxidant method (Figure 9), since 2,2-diphenyl-1-picryl-hydrazine is a non-physiological free radical, which is very similar to the reactive species ABTS (2,2′-azinobis-3-ethylbenzotiazoline-6-sulfonic acid). So, *Betulae extractum* (BE) was the most potent antioxidant of all, followed by *Liquiritiae extractum* (LE) and *Avenae extractum* (AE); the median inhibition of free radical reactivity DPPH per vegetal extract is highest for *Betulae extractum* (BE) and lowest for *Avenae extractum* (AE); and the variation in the number of points scored per plant extract is highest for *Betulae extractum* (BE) due to the box plot size. 

**Figure 10:**
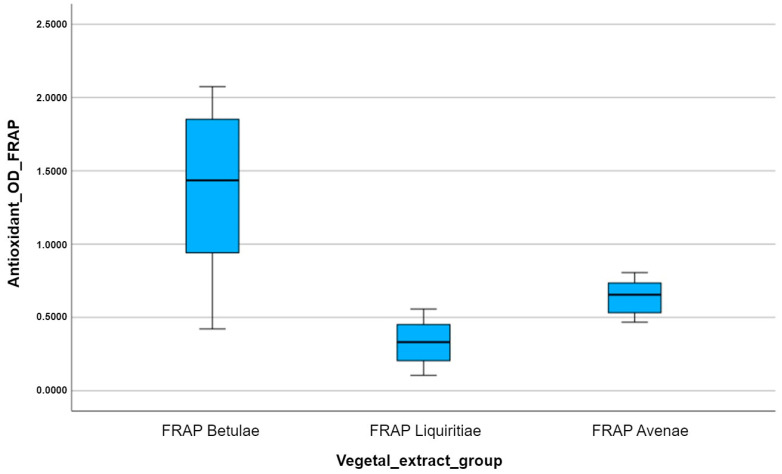
**A** comparison of the multiple box plots for the FRAP antioxidant method.

The comparative analysis of the multiple box plots for the FRAP antioxidant capacity of the vegetal extracts highlights the superior antioxidant potency of silver birch extract, in contrast to the other analyzed extracts (Figure 10). Using the FRAP assay, we obtain the lowest colorimetric reaction, in terms of obtained optical density, for licorice. The slightly different results compared to those obtained by the other two antioxidant methods can be explained by the different behavior of the tested plant extracts compared to the physiologically reactive species (ferric and ferrous ions), unlike the interaction of the extracts with non-physiological free radicals (ABTS and DPPH).

#### 2.6.2. Comparison of Antioxidant Activity between Multiple Plant Extract Groups

Statistically significant differences were found when comparing the free radical scavenging capacities of the analyzed vegetal extracts (one-way ANOVA). So, the differences between groups for the ABTS data set remain: ABTS Betulae vs. ABTS Avenae (*p* = 0.031, *p* < 0.05) and ABTS Liquiritiae vs. ABTS Avenae (*p* = 0.017, *p* < 0.05) (Table 6).

For the DPPH method, a test was performed to determine whether the difference between the groups occurred randomly or was statistically significant, and it was observed that there were statistically significant differences (*p* < 0.05) when comparing the antioxidant capacities of the studied plant extracts (One-Way ANOVA). Thus, after performing the Games–Howell post hoc test for unequal variances, it was found that there was a statistically significant difference between all the groups (Table 6): *DPPH Betulae* vs. *DPPH Liquiritiae* (*p* = 0.026, *p* < 0.05), *DPPH Betulae* vs. *DPPH Avenae* (*p* = 0.001, *p* < 0.05), and *DPPH Liquiritiae* vs. *DPPH Avenae* (*p* = 0.003, *p* < 0.05).

For the FRAP method, the results obtained by statistical validation using One-Way ANOVA showed statistically significant differences (*p* < 0.05) between the free radical reduction effects of the plant extracts (Table 6). After comparing the averages between groups for the FRAP method with the post hoc tests, statistically significant differences were observed, expressed by the *p* value as follows: *FRAP Betulae* vs. *FRAP Liquiritiae* (*p* = 0.000, *p* < 0.05), *FRAP Betulae* vs. *FRAP Avenae* (*p* = 0.006, *p* < 0.05), *FRAP Liquiritiae* vs. *FRAP Avenae* (*p* = 0.000, *p* < 0.05).

At each antioxidant method, the confidence interval was calculated, which indicates the degree of confidence that the statistical result did not occur by chance or by sampling error. The customary confidence level used was 95%, leading to a customary significance level, or p-value, of 5%. For every multiple comparison between groups, we have a lower bound and an upper bound calculated (the last two columns of Tables S8–S10 in Supplementary Material). In those cases, the 95% confidence interval was calculated from the mean and its standard error as a test of the reliability of the mean difference between the studied vegetal extract groups. 

#### 2.6.3. Correlation Analysis and Relationship Map

Chemical compounds found in vegetal extracts can be potent antioxidant molecules that act as reducing agents by donating electrons to free radicals in order to stabilize them and minimize the damage caused by free radicals to cells, DNA, and organ systems. There are some phytochemicals that contribute significantly to the antioxidant activity exerted by plant extracts due to their unique redox properties or their capability to scavenge reactive oxygen species effectively. Therefore, it is very important to quantify the existing relationship between the content of plant bioactive compounds and the antioxidant activity demonstrated in vitro. 

The aim of the Pearson correlation test applied in this study is to measure the magnitude, strength, and direction of a linear relationship between data. The Pearson correlation coefficient, also referred to as Pearson’s r value, was calculated for pairs of experimental data with different units of measure, establishing the correlation between continuous numerical variables (the concentration of active principles—(g active principle/100 g dry extract) and the antioxidant activity—(% inhibition) or (mg/mL)).

The Pearson correlation coefficient between the ABTS inhibition data set and flavonoid content (FL) revealed a very strong positive correlation (*FL* vs. *ABTS Betulae*: r = 0.943, *p* = 0.217; *FL* vs. *ABTS Liquiritiae*: r = 0.900, *p* = 0.288; *FL* vs. *ABTS Avenae*: r = 0.977, *p* = 0.137). 

The correlation analysis outlines a strong to very strong positive correlation between the DPPH inhibition data set and flavonoid content as follows: *FL* vs. *DPPH Betulae*: r = 0.979, *p* = 0.130 (very strong correlation); *FL* vs. *DPPH Liquiritiae*: r = 0.960, *p* = 0.181 (very strong correlation); *FL* vs. *DPPH Avenae*: r = 0.796, *p* = 0.414 (strong correlation). 

Similar results were obtained after the evaluation of the Pearson coefficient between the FRAP antioxidant method and the flavonoid content: *FL* vs. *FRAP Betulae*: r = 0.921, *p* = 0. 255 (very strong correlation); *FL* vs. *FRAP Liquiritiae*: r = 0.928, *p* = 0.244 (very strong correlation); *FL* vs. *FRAP Avenae*: r = 0.739, *p* = 0.471 (strong correlation). 

The values of the Pearson coefficient are positive for each evaluation made, which explains the direct correlation between the data (the higher the concentration of flavones, the greater the inhibition of free radicals by plant extracts; thus, the stronger the antioxidant activity.).

Regarding the relationship between the polyphenol content (TPC) and antioxidant activity, the Pearson coefficient highlighted a very strong negative correlation when compared with the EC50 obtained by the FRAP method (*TP* vs. *EC50 FRAP*: r = −0.954, *p* = 0.194). The negative Pearson coefficient, in this case, means an inverse relationship between data sets; as the TP concentration is higher, the EC50 value is lower, and the vegetal extract is a better antioxidant.

In order to quantify the relationship established between the antioxidant power and the concentration of active principles in the plant extracts, we created relationship maps. 

Relationship maps are useful for determining how variables relate to each other by providing a visual representation of the connections and influences that each node and link has over each other. Therefore, relationship maps visually represent connections and influences through nodes and links. Nodes represent variables and variable categories; links represent the strength of influence between nodes. Larger nodes and thicker link lines represent stronger connections and influence. Smaller nodes and thinner link lines represent weaker connections and influence.

**Figure 11:**
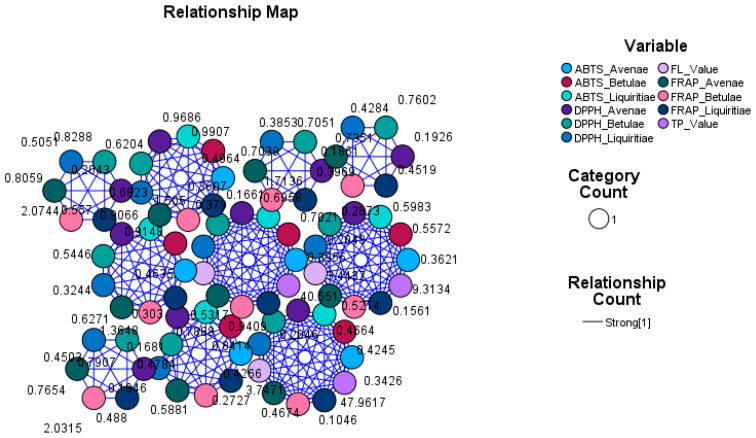
Relationship Map Circle Layout. ABTS, DPPH, FRAP—antioxidant method; FL value—flavonoid content; TP value—total phenolic content.

In the relationship map, which represents the connections between different antioxidant methods (ABTS, DPPH, and FRAP) applied to the three vegetal extract groups (*Betulae extractum*, *Liquiritiae extractum*, and *Avenae extractum*) and the concentration of bioactive compounds (total polyphenols and flavones), we can observe the thickness of each link that represents the strength of the relationship (so, the thicker the link line is, the stronger the relationship), but there are also visible the nodes for each variable (the bigger the nodes, the more influence as there are more cases related to that node) (Figure 11). A symmetry of the size of the nodes was obtained for all the tested variables and a uniformity of the connections, which highlights a strong relationship confirmed by using this graphic tool.

### 2.7. Molecular Docking

Molecular docking simulations were carried out to investigate the potential interactions between phytochemicals detected through GC-MS and Keap1, targeting both noncovalent and covalent binding sites. The docking protocol was validated by redocking the experimentally determined co-crystallized ligands and calculating RMSD values. For the Kelch domain noncovalent binding site, chromone-2-carboxylic acid showed a binding energy of −7.204 kcal/mol and a ligand efficiency of 0.515 (Supplementary Material Table S11), with the RMSD of the predicted pose being 0.0485Å (Supplementary Material Figure S54a). After docking the covalent inhibitor bardoxolone in the BTB domain at Cys151, the triterpenoid ligand exhibited a binding energy of −12.600 kcal/mol, and the LE was 0.350 (Supplementary Material Table S12), while the calculated RMSD after superposition on the initial conformation was 0.4927 Å (Supplementary Material Figure S54b). Thus, both co-crystallized ligands showed only slight variations in the binding pose after simulation, indicating that the docking procedure was successfully validated. Moreover, bardoxolone was covalently docked at three other cysteine residues, and results suggested that the ligand can react only with Cys151. For Cys171, no binding affinity could be estimated for bardoxolone, since steric clashes with the protein backbone and sidechain atoms prevented successful docking into her protein cavity. For Cys77, the triterpenoid derivative showed a positive binding energy (3.700 kcal/mol), which practically suggests no binding whatsoever, while, for Cys434, the predicted binding energy was −8.390 kcal/mol, which was significantly higher than the value obtained for Cys151. Therefore, the docking protocol successfully discriminated between residues that are reactive toward bardoxolone.

Since there are no solved complex structures for ligands that covalently bind reactive residues Cys77, Cys171, and Cys434, we docked the known ligands, and the generated models were validated by analyzing the predicted poses. For Cys171 (BTB domain), we docked the confirmed ligand pterisolic acid B as a positive control (Supplementary Material Figure S54d). Pterisolic acid had three unsatisfied hydrogen bond acceptor atoms (the unsaturated ketone and carboxylic acid moiety), but it formed two hydrogen bonds through the hydroxyl moiety. The predicted pose had a binding energy of −13.450 for Cys171, and the binding affinities for the other cysteines were practically lower. For Cys77 (BTB domain) and Cys434 (Kelch domain), we chose pubescenoside A as a positive control and known ligand. The binding mode of pubescenoside (Supplementary Material Figure S54c,e) was considered valid when it formed similar interactions as those described by the paper that identified it as a ligand [28]. Pubescenoside showed a binding energy of −17.790 kcal/mol for Cys77, and the binding affinity was similar for Cys171. However, the ligand conformation after binding to Cys171 was too strained to be considered plausible. Moreover, pubescenoside showed a better binding affinity for Cys434 than for Cys151. 

Noncovalent molecular docking results after simulating the phytochemicals detected in all three plant extracts are shown in Supplementary Material Table S11. The docking experiments yielded binding energies that varied between −10.112 and −5.928 kcal/mol (−7.7374 ± 1.113 kcal/mol), and the ligand efficiencies ranged from 0.110 to 0.539 (0.323 ± 0.102). The best docking score was obtained for glycyrrhizin, which, however, showed the lowest ligand efficiency. The lowest docking score was observed for cinnamic acid, which in turn showed the highest ligand efficiency. Seven out of twenty-six compounds had binding affinities lower than the positive control (aucubin, betuloside, caffeic acid, dihydrocaffeic acid, cinnamic acid, ferulic acid, and p-coumaric acid), while only one cinnamic acid had a higher ligand efficiency than chromone-2-carboxylic acid. However, high binding affinities (>0.350) were obtained for seven other compounds: caffeic acid, dihydrocaffeic acid, ferulic acid, p-coumaric acid, kaempferol, isoliquiritigenin, and liquiritigenin.

**Figure 12:**
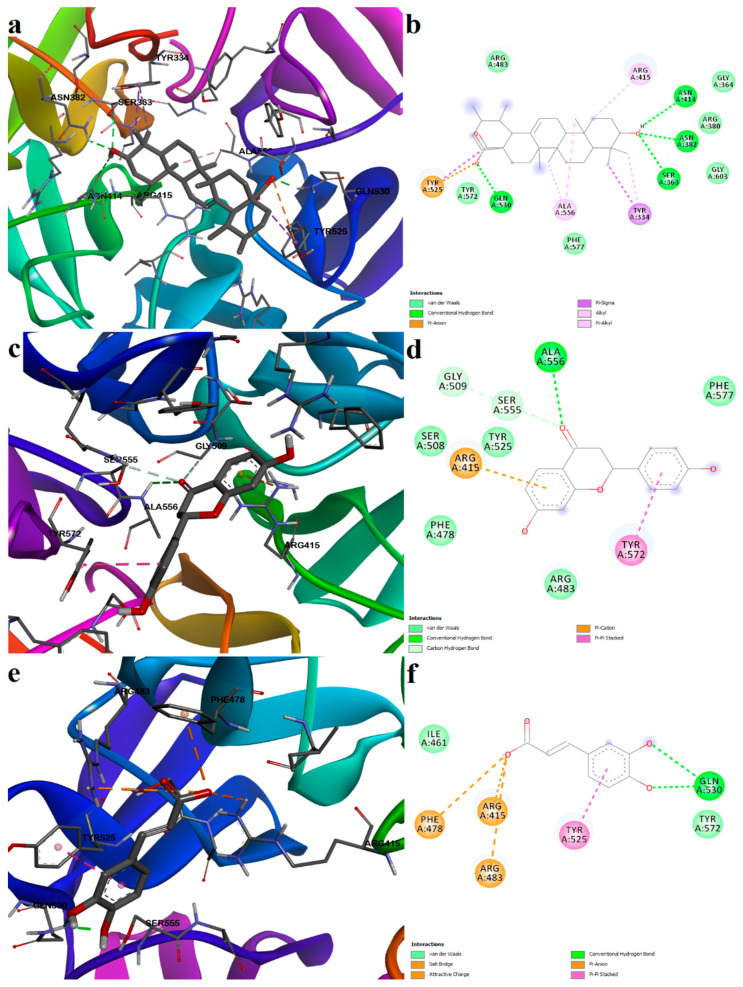
Predicted protein–ligand complexes after noncovalent docking with the Keap1 Kelch domain. (**a**)—binding pose of ursolic acid; (**b**)—two-dimensional depiction of the protein–ligand interactions between Keap1 and ursolic acid; (**c**)—binding pose of liquiritigenin; (**d**)—two-dimensional depiction of protein–ligand interactions between Keap1 and liquiritigenin; (**e**)—binding pose of caffeic acid; (**f**)—two-dimensional depiction of protein–ligand interactions between Keap1 and caffeic acid.

Ursolic acid is one of the identified pentacyclic triterpenoid derivatives that showed good binding potential with the Keap1 Kelch domain. Ursolic acid showed the second-highest binding affinity as a noncovalent ligand. The triterpenoid formed five polar interactions, such as four hydrogen bonds with Ser363, Asn382, Asn414, Gln530, and a pi–anion interaction between the carboxylic moiety and Tyr525. Moreover, several hydrophobic interactions stabilize the complex: alkyl and pi–alkyl interactions with Arg415, Ala556, pi–sigma interactions with Tyr334 and Tyr525, and several van der Waals interactions with other residues within the binding pocket (Figure 12a,b). 

Liquiritigenin and caffeic acid showed preferential binding to the same sub-pocket that the positive control occupies. Moreover, liquiritigenin is a flavone that shares the same structural scaffold as chromone-2-carboxylic acid (the benzopyran-4-one substructure), and it had a binding energy of −7.226 kcal/mol. The ligand formed four polar interactions: a conventional hydrogen bond with Ala556, two carbon-hydrogen bonds with Gly509 and Ser555, and a pi–cation interaction with Arg415. Moreover, a stacked pi interaction was formed with Tyr572, and van der Waals interactions were formed with five other residues (Figure 12c,d).

Caffeic acid, dihydrocaffeic acid, p-coumaric acid, and ferulic acid are structurally similar to 3-(4-hydroxyphenyl) propanoic acid, another known inhibitor of the Keap1–Nrf2 interaction [25]. All four compounds had similar binding energies, ligand efficiencies, and formed four or five polar interactions with the receptor. The carboxylic moiety of caffeic acid interacted with two arginine residues (Arg415 and Arg483) within the binding sites through a salt bridge and attractive charges, and with Phe478 through pi–anion interactions. Furthermore, the aromatic hydroxyl groups formed two hydrogen bonds with Gln530. The protein–ligand complex is further stabilized by hydrophobic interactions, such as pi–pi stacked and van der Waals interactions (Figure 12e,f).

**Figure 13:**
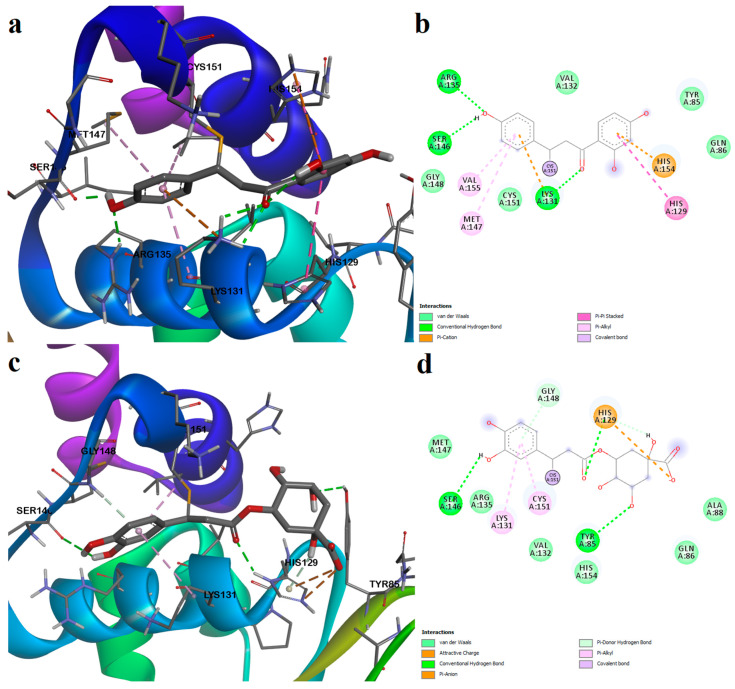
Predicted protein–ligand complexes after covalent docking with Cys151 (Keap1 BTB domain). (**a**)—binding pose of isoliquiritigenin; (**b**)—two-dimensional depiction of protein–ligand interactions between Keap1 and isoliquiritigenin; (**c**)—binding pose of chlorogenic acid; (**d**)—two-dimensional depiction of protein–ligand interactions between Keap1 and chlorogenic acid.

We identified, through GC-MS analysis, only two phytochemicals suitable for covalent docking, isoliquiritigenin and chlorogenic acid (Figure 13), since their chemical structures contain α, β-unsaturated carbonyl moieties (ketones). Such conjugate additions cannot occur under normal conditions for unsaturated carboxylic acids, and thus compounds such as cinnamic acid cannot react with the nucleophilic cysteines. 

The binding affinities for isoliquiritigenin increased in the following order: Cys77 < Cys151 < Cys434 < Cys151 (Table S6). However, binding pose analysis revealed that the most favorable conformation was obtained after its reaction with Cys151, the binding energy being −11.560 kcal/mol. The polyphenolic compound isoliquiritigenin also formed weak interactions with residues that bind the positive control bardoxolone. Thus, isoliquiritigenin formed hydrogen bonds with Arg135, Ser146, and Lys131 and formed pi–cation interactions with His154 and Lys131. Moreover, the aromatic rings interacted with other residues that form the binding pocket through pi–pi stacked, pi–alkyl, and van der Waals interactions (Figure 13a,b).

Chlorogenic acid, a polyphenol (the ester of caffeic acid and quinic acid), showed the highest affinity for Cys171, followed by Cys151, Cys77, and Cys434. The most optimal binding pose was observed after binding to Cys151, and the predicted conformation was rather similar to isoliquiritigenin (Figure 13c,d). The reactive Cys151 formed both strong (covalent bond) and weak (pi–alkyl) interactions with chlorogenic acid. Moreover, the aromatic hydroxyl moiety formed a hydrogen bond with Ser146. Two more hydrogen bonds are formed with Tyr85 and His129. Similar to the positive control (bardoxolone), the carboxylic acid moiety of chlorogenic acid interacted with the positively charged His129 through attractive charges and pi–anion interactions. A pi–alkyl interaction was also formed with Lys131, and several van der Waals interactions were also noted.

**Figure 14:**
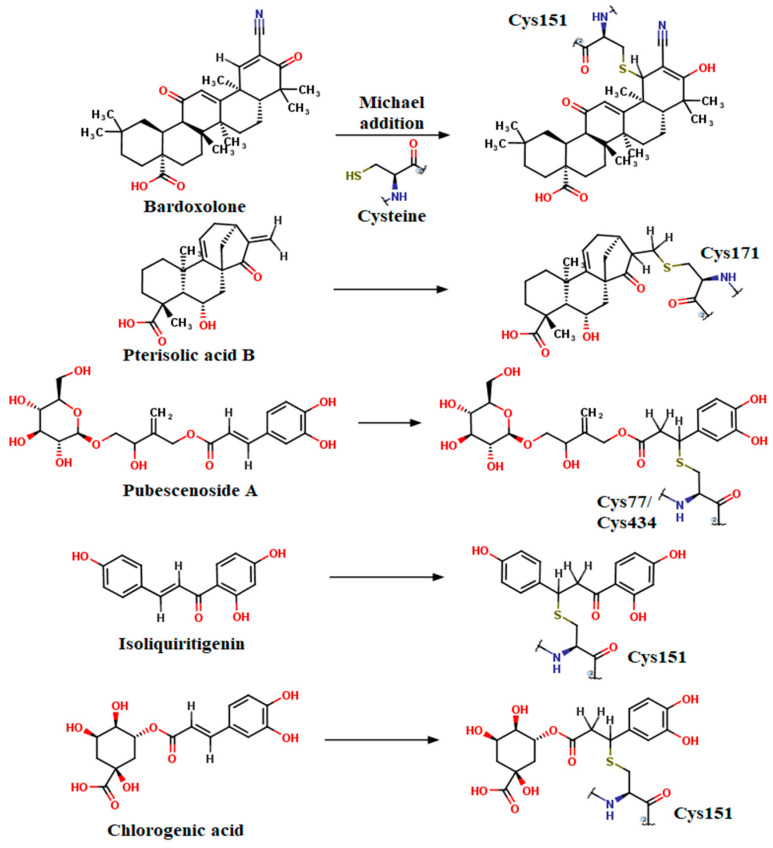
Michael reaction between docked compounds (acceptors) and reactive cysteines (donors) situated in the Keap1 BTB domain (Cys77, Cys151, Cys171) and Kelch domain (Cys434).

In Figure 14, we illustrated the Michael addition reaction between the used positive controls (bardoxolone, pterisolic acid B, and pubescenoside A), isoliquiritigenin, chlorogenic acid, and reactive cysteines of Keap1, and all ligands acted as electrophilic acceptors through the α, β-unsaturated carbonyl moiety.

## 3. Discussion

Ethanol, used as the extraction solvent, provides many advantages compared with other organic solvents in terms of safety (it is recognized as GRAS, Generally Recognized as Safe), costs, compatibility, and it has the capacity to extract more metabolites than other mixtures with water, mainly due to its polarity. A high polarity provides the ability to extract a wider variety of compounds (phenols, flavonoids, catechols, tannins, etc.) and to obtain better extraction yields [40]. Thus, for the extraction processes, 50% ethanol was used. For licorice root extract, our obtained yield (20%) was comparable with that obtained by Chandrasekharan Dhanya et al. (29%), using absolute ethanol [41]. 

The phytochemical profile was achieved using complementary techniques for the phytochemical compound’s identification and quantification. The polyphenols play an important role in the antioxidant activity because these types of compounds have aromatic rings that allow them to act as reducing agents due to their phenolic hydrogen atoms’ mobility, stabilization, and relocation of the unpaired electrons of their structures and due to their metal chelating properties [42]. The extraction process, using 50% ethanol, allowed us to obtain an extract enriched in polyphenols. Ilva Nakurte et al. [43] obtained a birch outer bark ethanolic extract (from *Betula pendula* Roth) in three extraction stages, and they found a total polyphenol content in the range of 2.56 to 7.42 g of gallic acid equivalents per 100 g of extract. Farag Mohamed et al. obtained a licorice methanolic extract with 1 to 5% polyphenol content. Our findings revealed a content of polyphenols of 47.9617 ± 9.7083 for BE and 9.3134 ± 0.9913 for LE, expressed as g tannic acid/100 g dry extract. For LE, the main phenolic compounds are liquiritigenin and its chalcone-type derivative, isoliquiritigenin, which are responsible for its antioxidant activity. Additionally, glabridin (an isoprenylated flavonoid) plays an important role in antitumoral activity due to its P450 enzyme inhibition properties [44]. In the case of AE, many study references are found about oat seeds, but not so many for oat young herb. The total polyphenol content obtained in our study was very close to the BE content (40.551 ± 6.3715 g tannic acid/100 g dry extract) and about four times higher than the LE content. In the case of total flavonoid content, BE and LE had the highest results, expressed as g of rutoside/100 g dry extract, almost twice the quantity found in AE: 3.7471 ± 0.3140 (BE), 3.4437 ± 0.3037 (LE), and 1.9471 ± 0.0526 (AE). Concerning the total phenolic acid content, only BE was found to contain 25.3448 ± 1.6728, expressed as g chlorogenic acid/100 g dry extract. 

The GC-MS analysis, in the case of LE, revealed many compounds grouped into several classes, such as fatty acids, tannins, carboxylic acids, alpha-hydroxy carboxylic acids, amino acids, phytoestrogens, and sugars. Findings related to phytochemical analysis showed that 50% of the dry weight of *Glycyrrhiza* roots is defined by water-soluble compounds, such as sugars (5–15%; glucose, sucrose, and mannitol), 3–10% D-glucose, and amines (1–2%) [45]. Other studies’ findings showed a sucrose content of 45–58% [44], very close to our results (54%). Additionally, for LE, GC-MS analysis revealed 3% D-glucose, 1% amino acids, 85% sugars, and 2.5% alpha-hydroxy carboxylic acid contents. In the case of BE, GC-MS analysis revealed many important classes of compounds, including triterpenes (38%), tannins (2.5%), carboxylic acids (9%), alpha-hydroxy carboxylic acids (1%), and sugars (37%). *Avenae extractum* also has a number of compound classes identified through GC-MS analysis: hexoses (39.43%), disaccharides (29.84%), carboxylic acids (21.99), fatty acids (3.57%), polyols (2.20%), and amino acids (1.78%). Additionally, gas chromatography showed a higher linoleic and linolenic acid content in AE than in BE and LE (0.19 g % linoleic acid and 0.92 g % linolenic acid). 

Due to their thermolability, the triterpene saponins and flavonoids have difficulty being directly analyzed by GC-MS. Using derivatization methods, which involve hydrolysis followed by methylation and silylation, some compounds could have been identified [46]. Through applied GC methods, we could not identify triterpenes or flavonoids in licorice root extract, but their presence was confirmed by FT-ICR, which was based on specific molecular weight: liquiritigenin, isoliquiritigenin, glabridin, glycyrrhetinic acid, and glycyrrhizin. The same situation was applied to AE, too; GC-MS could not identify any saponin or flavonoids, but FT-ICR confirmed the presence of avenacoside A (saponin) and tricin and vitexin (flavonoids). This was not the situation for BE, where GC-MS analysis, through the derivatization method, showed a 38% triterpene content: 1.5% ursolic acid, 1.4% oleanolic acid, 2.87% betulin, 29% lupeol, and 2.5% betulin aldehyde. Their confirmation was also supported by FT-ICR analysis: betulinic acid, betulin, lupeol, betulinaldehide, lupenone, betulonic acid, betuloside, caffeic acid, and kaempferol.

In the literature, information about the antioxidant activity of *Betulae folium* extract can be found easily from previous studies (including extract obtained with 50% ethanol [47,48]), but fewer information can be found for the *Betulae cortex* extract. In our study, for all three in vitro methods used to evaluate the antioxidant activity, the results showed a higher antioxidant activity for BE compared with LE and AE. This antioxidant activity is positively correlated with the results from the total polyphenols determination, with BE having a higher content than LE and AE. For the ABTS method, the performed samples showed different reaction kinetics for the three extracts, as well as the interdependence between the phytochemical profile and the free radical reaction time. BE has a very high reaction time, much higher than other plant extracts, annihilating reactive oxygen species (ROS) in a very short time, even at very low concentrations. *Betulae extractum* has a strong antioxidant action, having an IC50 value very close to the standard used as a reference (ascorbic acid—0.0165 mg/mL).

The values of the Pearson coefficient (r) are negative in the case of the correlation of the antioxidant effect with the concentration of active principles (TPC), which explains the inverse correlation between the data (the higher the amount of active principles, the lower the IC50 value of the extracts, and so the antioxidant action is stronger). The total polyphenols content (TPC) is in direct correlation with the antioxidant activity of plant extracts, and the compared data (TPC concentration vs. IC50 value) show a very strong correlation in the case of all antioxidant determination methods (ABTS, DPPH, and FRAP) (|r| is between 0.900 and 1.000).

Considering that, from a mathematical point of view, the Pearson correlation coefficient (r) has certain intervals that express the degree of correlation between the experimental data sets, where we have values lower than 0.900 of the r coefficient, it is possible to quantify the existing relationship between the analyzed data, even if statistically, the results obtained for the entire target population cannot be extrapolated, because, for the correlation between the antioxidant activity and the concentration of polyphenols, *p* > 0.05 was obtained.

The Pearson correlation between the methodologies used in this study (DPPH, ABTS, and FRAP) to evaluate the antioxidant action in the extracts analyzed for each set of data pairs obtained by different methods shows values above 0.900, which explains a very strong and statistically significant correlation (*p* = 0.0001) between the experimentally applied methodologies. The Pearson correlation results between the methods (r > 0.900), as well as the coefficients of determination (R^2^ > 0.900), reveal a very well correlated antioxidant activity of the extracts that present independent values, and it is not significantly influenced by methodology errors.

Molecular docking simulations were also performed to evaluate the possibility of the analyzed phytochemicals to stimulate the Keap1-Nrf2 pathway. Keap1, the inhibitor of nuclear factor Nrf2, is an attractive biological target for alleviating many diseases that are characterized by pronounced oxidative stress and inflammation, including various dermatological disorders [30,31,32,33]. Keap1 can be pharmacologically inhibited either by covalent modifications of the cysteine residues by electrophiles, or by impeding its direct interaction with Nrf2 [25,27,28,29]. The docking experiments revealed that many of the screened phytochemicals have the potential to act as noncovalent binders at the Keap1–Nrf2 binding interface. Several pentacyclic triterpenoids were analyzed, such as betulin, betulinaldehyde, betulinic acid, betulonic acid, lupeol, lupenone, oleanolic acid, and ursolic acid. Among these compounds, ursolic acid showed the best binding potential toward the Keap1 Kelch domain, and other studies revealed, in fact, that the natural compound activates Nrf2 and promotes neuroprotection in mice models of cerebral ischemia and brain injury [49,50]. Moreover, other authors reported that betulinic acid, lupeol, and oleanolic acid can also activate the Nrf2 pathway [51,52,53]. The aglycone of glycyrrhizin (a saponin) is also a triterpenoid derivative (glycyrrhetic acid or enoxolone), and glycyrrhizin showed the highest binding affinity for the Kelch domain and formed the highest number of polar interactions with the residues within the binding site. Interestingly, previous studies showed that glycyrrhizin activated the Nrf2 pathway and ameliorated fat-diet-induced obesity in rats [54].

Cinnamic acid showed a very good binding affinity and formed five electrostatic interactions. Although cinnamic acid showed a low binding energy, other studies indicated that it is inactive towards Nrf2, and only its esters showed biologic activity [55]. Therefore, the docking results for cinnamic acid can be regarded as false positives. On the other hand, caffeic acid showed similar binding affinity for the Kelch domain, and other authors revealed that it actually activated the ERK/Nrf2 pathway in HepG2 cells [56]. Furthermore, ferulic acid and p-coumaric acid, which are structurally similar to caffeic acid, were also shown in preclinical studies to stimulate the Nrf2 pathway [57,58].

Liquiritigenin is a dihydroxyflavanone that was shown to activate the Nrf2 antioxidant defense system [59]. We, herein, presented a potential binding conformation of liquiritigenin within the Keap1 Kelch domain, with the possibility of disrupting the Keap1–Nrf2 interaction and promoting nuclear translocation of Nrf2. Moreover, liquiritigenin occupied the same binding subpocket as caffeic acid. Isoliquiritigeniin, on the other hand, was shown to covalently bind with Cys151 within the BTB domain of Keap1 [36]. By molecular docking simulations, we observed that isoliquiritigenin also forms many favorable noncovalent interactions with the binding pocket. Moreover, we showed that chlorogenic acid binds to Cys151 in a similar manner, with a satisfactory binding energy. Chlorogenic acid and its isomers were shown to directly interact with Keap1 in Caco-2 cells and stimulate Nrf2 signaling [60]. Furthermore, chlorogenic acid is the ester of caffeic acid and quinic acid and could also activate Nrf2 after hydrolysis to caffeic acid. 

Docking studies also yielded interesting results for kaempferol (flavonol), vitexin (apigenin flavone glucoside), and tricin (O-methylated flavone). Previous studies highlighted that vitexin can protect melanocytes from oxidative stress by activating the MAPK-Nrf2/ARE signaling pathway [61], while conflicting results were observed for kaempferol, since it can either downregulate Nrf2 mRNA in non-small cell lung cancer cells [62] or elevate the protein levels of Nrf2 and HO-1 in aortic tissues [63]. Other screened compounds, such as glabridin and aucubin, had modest interactions with Keap1, but were shown, in previous studies, to regulate Nrf2 signaling [64,65]. The three analyzed extracts possess a great potential of exerting antioxidant and anti-inflammatory effects in vivo, and further studies are warranted to assess their therapeutic potential.

## 4. Materials and Methods

### 4.1. Plant Material

Plant materials, *Glycyrrhiza glabra* L. root and *Betula alba* var. *pendula* Roth. bark, were purchased online, from the United Kingdom (in 2021), in dried states. The birch bark was collected from the northerly climates of Europe. *Avena sativa* young herb, harvested when it is 20–25 cm tall, was purchased from Romania, in 2021, in the form of a powder, obtained by freezing to −40 °C, then slow drying under advanced vacuum and very fine grinding (200 mesh). Before the extraction process, the birch bark and licorice root were ground and homogenized using a Cyclotec 1093 laboratory mill. The oat powder was used, as it was purchased, without further grinding. The content of the water was max. 7% for birch bark, max. 8% for licorice root, and max. 6.5% for oat herb.

### 4.2. Chemicals

All the chemicals used were analytical-reagent grade, and the water was ultrapure, obtained with a Millipore Integral 3 water purification system. The chemicals included: hexane (Sigma-Aldrich, Germany), methanol (Sigma-Aldrich, Germany), boron fluoride (Sigma-Aldrich, Germany), sodium hydroxide (Sigma-Aldrich, Germany), N,O-Bis(trimethylsilyl)trifluoroacetamide (BSTFA) derivatization reagent (Sigma-Aldrich, Germany), acetonitrile RS (Carlo Erba, France), methanol for LC-MS (Honeywell, Germany), acetonitrile for HPLC (Sigma-Aldrich, Germany), ethanol (Sigma-Aldrich, Germany), sodium acetate (Sigma-Aldrich, Germany), aluminum chloride (Sigma-Aldrich, Germany), rutoside (rutin, Sigma-Aldrich, Germany), tannic acid (Sigma-Aldrich, Germany), hydrochloric acid 0.5 N (Sigma-Aldrich, Germany), Arnow reagent (sodium nitrite, Sigma-Aldrich, Germany), chlorogenic acid (Sigma-Aldrich, Germany), 2,2 diphenyl-1-picrylhydrazyl (DPPH) (Sigma-Aldrich, Germany), ascorbic acid (vitamin C) (Roth, Germany), 2,2′-(1,2-hydrazinediylidene)bis[3-ethyl-2,3-dihydro-6-benzothiazolesulfonic acid, diammonium salt (ABTS ammonium salt) (Sigma-Aldrich, Germany), potassium persulfate (Merck, Germany), trichloroacetic acid (Merck, Germany), ferric chloride (Sigma-Aldrich, Germany), phosphate buffer, and potassium hexacyanoferrate (III) solution pH 6.6 (Sigma-Aldrich, Germany).

### 4.3. Extraction Method and Extraction Process Yield

The extraction process was carried out in two successive stages with 50% ethanol (for the first stage, the ratio of plant material to 50% ethanol was 1:10, after a soaking process of the plant material, and, for the second one, it was 1:5 for 100 g of plant material, the combined extractive solutions being subjected to a concentration process on a rotary evaporator (Buchi R 210-215) and, subsequently, to a drying process by lyophilization (Christ Alpha 1-2/Braun, BiotechInt., India). The solvent was chosen mainly because of its properties to obtain very good extraction yields and extracts with high polyphenols content. Based on the plant material mass taken into the process and the total weight of extract obtained, the extraction process yield was calculated.

### 4.4. Determination of Total Flavonoid Content (TFC), Total Polyphenol Content (TPC), and Total Phenolic Acid Content (TPA) Using Spectrophotometric Methods

#### 4.4.1. Determination of the Total Flavonoid Content (TFC)

For the total flavonoid content determination, a colorimetric method, based on the reaction of flavone heterosides and free aglicons with aluminum chloride (AlCl_3_), was used, thus obtaining yellow reaction compounds. Aliquots of 0.1 g extract were dissolved in 25 mL of 50% ethanol. Volumes of 0.4 mL, 0.6 mL, 0.8 mL, 1.0 mL, and 1.2 mL were transferred to 10 mL volumetric flasks. In each flask were added 2 mL of sodium acetate 100 g/L and 1 mL of aluminum chloride 25 g/L, and the volumes were adjusted to 10 mL with 50% ethanol. At the same time, proper control samples were prepared in the same manner as described above, but without the addition of sodium acetate and aluminum chloride. After 30 min, the absorbance was measured at 427 nm (Jasco V-530 spectrophotometer, Tokyo, Japan). Rutoside (rutin) was used as the standard for the linear calibration curve in the concentration range of 5 to 35 µg/mL, with R^2^ = 0.9992 (Supplementary Materials Figure S32). The results (TFC) were expressed as mg of rutin equivalents per gram of sample (mg/g) [66].

#### 4.4.2. Determination of the Total Polyphenol Content (TPC)

The determination of total polyphenol content (TPC) was based on the reaction of Folin-Ciocalteu [67]. Aliquots of 0.05 g extract were dissolved in 100 mL of 50% ethanol. Volumes of 0.5 mL, 0.6 mL, 0.7 mL, 0.8 mL, and 0.9 mL were transferred to 10 mL volumetric flasks and adjusted to 1 mL by adding distilled water. After that, each sample was mixed with 1 mL of Folin-Ciocalteu phenol reagent and adjusted to 10 mL with sodium carbonate solution 200 g/L. The samples were shaken and kept in dark conditions for 40 min. Then, the absorbance was measured at 725 nm (Jasco V-530 spectrophotometer, Tokyo, Japan) relative to a blank sample (1 mL of distilled water, 1 mL of Folin-Ciocalteu phenol reagent and 8 mL of sodium carbonate). As a standard for linear calibration, we used tannic acid in the concentration range of 2.04 to 9.18 µg/mL, with R^2^ = 0.999 (Supplementary Materials Figure S33). The results (TPC) were expressed as mg of tannic acid equivalents per gram of sample (mg/g).

#### 4.4.3. Determination of the Total Phenolic Acid Content (TPA)

The determination of the total phenolic acid content (TPA) was based on the reaction between phenolic acids and nitric acid, resulting in nitro derivatives. Aliquots of 0.05 g extract were dissolved in 100 mL of 50% ethanol. Volumes of 0.8 mL, 1.0 mL, 1.2 mL, 1.4 mL, and 1.6 mL were transferred to 10 mL volumetric flasks. In each flask, we added 2 mL of hydrochloric acid 0.5 N, 2 mL of Arnow reagent (sodium nitrite solution 100 g/L), and 2 mL of sodium hydroxide 85 g/L and adjusted to 10 mL by adding distilled water. Immediately, the absorbance was measured at 525 nm (Jasco V-530 spectrophotometer, Tokyo, Japan) relative to a sample without Arnow reagent. As a standard for the linear calibration curve, we used chlorogenic acid in the concentration range of 11.3 to 52.8 µg/mL, with R^2^ = 0.9998 (Supplementary Materials Figure S34). The results (TPA) were expressed as mg of chlorogenic acid equivalents per gram of sample (mg/g) [66].

### 4.5. GC-MS Analysis

The GC-MS analysis of the ethanolic extracts was performed using a Thermo Scientific TRACE 1310 gas chromatograph, coupled with a triple quadrupole mass spectrometer (TSQ 8000 EVO), with TRIPLUS RSH injection module and SCAN acquisition. The analysis procedure involved three methods: the derivatization method, fatty acid determination, and volatile compound determination.

#### 4.5.1. Derivatization Method

For this GC method, 20 mg of each extract was weighed in derivatization vessel, and 0.5 mL acetonitrile and 0.5 mL BSTFA were added; then, the vessel was stapled and subjected to derivatization at a temperature of 105 °C, for 1 h. After cooling, the vessel was uncapped, and the sample was filtered, using PTFE 0.2 µm filters, and analyzed.

Chromatographic analysis was performed on a TG-5SILMS column (5% diphenyl/95% dimethyl polysiloxane), 30 m × 0.25 mm × 0.25 µm, with helium as a carrier gas (1 mL/min). Experimental conditions were: injection volume, 1 µL; split ratio, 60; purge flow, 3 mL/min; injector temperature, 280 °C. Oven heating was programmed from 100 to 170 °C, with 10 °C/min and from 170 °C to 300 °C, with 5 °C/min, where it remained constant for 23 min. The column was connected to the quadrupole ionic source at a temperature of 230 or 280 °C. Mass spectra were recorded from 40 to 600 amu and stored until data processing.

Integrated chromatographic peaks were identified using the National Institute of Standards and Technology (NIST) library, version 2.2. The percentage ratio was calculated by relating the individual area of each identified compound to the sum of all the integrated areas. This method did not identify all the compounds, only those that are suitable for these analysis conditions.

#### 4.5.2. A Specific Method for the Identification of the Fatty Acids

Starting from the derivatization method, in which all the (some)classes of compounds identified in the extracts are revealed, a specific method for the identification of fatty acids was performed: 100 mg powder (precisely weighed) was treated with hexane, 0.5 M methanolic solution of sodium hydroxide and methanol, and kept at 80 °C for 30 min. Then, the sample was treated with 14% boron fluoride solution and kept at 80 °C for 10 min. After cooling, we added sodium chloride solution, and, after the phase separation, we analyzed the upper hexane phase after filtration through PTFE 0.2 µm filter.

Chromatographic analysis was performed on a TG-5SILMS column (5% diphenyl/95% dimethyl polysiloxane), 30 m × 0.25 mm × 0.25 µm, with helium as a carrier gas (1.2 mL/min). Experimental conditions were: injection volume, 0.5 µL; split ratio, 1:100; purge flow, 3 mL/min; injector temperature, 250 °C. Oven heating was programmed from 170 to 200 °C, with 3 °C/min and from 200 to 260 °C, with 5 °C/min, where it remained constant for 2 min. The column was connected to the quadrupole ionic source at a temperature of 230 °C. Mass spectra were recorded from 40 to 500 amu and stored until data processing. 

The identification was based on the comparison with the spectra of the standard components and with the spectra from the MS library.

The calibration was performed for two components: linoleic acid and linolenic acid, prepared individually and mixed in a solution of appropriate concentrations (correlated with the levels in the samples).

#### 4.5.3. Determination Method of Volatile Substances

For the volatile compounds, determination was performed using the "headspace” technique, where the extracts were weighed directly into the headspace bottle, and 5 mL of purified water were added before analysis.

Chromatographic analysis was performed on a TG-5SILMS column (5% diphenyl/95% dimethyl polysiloxane), 30 m × 0.25 mm × 0.25 µm, with helium as a carrier gas (1.2 mL/min). The experimental conditions were:

- incubation (sample volatilization in the thermostat), 10 min at 90 °C; syringe temperature, 100 °C;
- injection volume, 200 µL; split ratio, 5; purge flow, 3 mL/min; injector temperature, 250 °C. Oven heating was programmed from 50 °C to 220 °C, with 20 °C/min, where it remained constant for 1.5 min.

The column was connected to the quadrupole ionic source at a temperature of 230 or 250 °C. Mass spectra were recorded from 50 to 500 amu and stored until data processing.

### 4.6. Fourier-Transform Ion Cyclotron Resonance Mass Spectrometry (FT-ICR-MS) Analysis

FT-ICR-MS analysis was performed with a high-resolution mass spectrometer with a 15 T superconducting magnet (solar X-XR, QqqFT-ICR HR, Bruker Daltonics, Germany), using the electrospray ionization (ESI) technique (HR-MS). For the negative ESI ionization, the sample was introduced by direct infusion, with a flow rate of 120 µL/h, with a nebulizing gas pressure (N_2_) of 4 bar at 200 °C and a flow rate of 7 L/min. The spectra were recorded over a mass range between 46 and 800 uam, at a source voltage of 5700 V. For the positive ESI ionization, the sample was introduced by direct infusion, with a flow rate of 120 µL/h, with a nebulizing gas pressure (N2) of 3.2 bar at 180 °C, and a flow rate of 5 L/min. The spectra were recorded in a mass range between 46 and 800 uam, at a source voltage of 5500 V. Metabolites were identified by the isotopic patterns of the molecular ions and the exact mass. Because of the high mass resolution involved in the FT-ICR analysis, the compounds can be identified by the precise isotopic pattern. The SolariX software provides high-resolution predicted isotopic pattern, which can be compared with the experimental spectral data.

### 4.7. Radical Scavenging Activity

The antioxidant properties of the plant extracts were determined by three in vitro methods, including the 2,2 diphenyl-1-picrylhydrazyl (DPPH) scavenging method, ABTS antioxidant activity method, using as oxidant ABTS (2,2′-azino-bis-3-ethylbenzthiazoline-6-sulphonic acid), and ferric reducing antioxidant power (FRAP). There are multiple methods of testing the antioxidant properties (in vitro and in vivo) [68,69], but the in vitro assays are simple, rapid, and inexpensive to perform. The ABTS method is based on scavenging of stable ABTS^•+^ radical by antioxidants, where the radical loses its blue coloration, being a discoloration reaction [70]. It can be applied to determine the antioxidant capacity for both hydrophilic and lipophilic compounds. The sample’s absorbance was measured at the wavelength 734 nm (maximum absorbance value for ABTS radical) after a reaction time of 4 min. All the measurements were carried out in triplicate. The reduction in the absorbance values represents the inhibition of the ABTS^•+^ solution and was calculated according to the following equation [66]:

ABTS^•+^ Inhibition (%) = ((Absorbance of ABTS − Absorbance of ABTS + sample extract)/Absorbance of ABTS) × 100

In the FRAP method, the evaluation of antioxidant capacity is based on the calculation of the low pH mixture of Fe^3+^-2,3,5-triphenyl-1,3,4-triaza-2-azoniacyclopenta-1,4-dienechloride (TPTZ) reduction to Fe^2+^-tripyridyltriazine (colored in violet-blue). The reaction is monitored at a wavelength of 700 nm. All the measurements were carried out in triplicate. The results were obtained by comparing the absorbance change in the test mixture with the absorbance change for increasing concentrations of Fe^3+^ [69,70].

The DPPH method is the most popular one for testing the antioxidant activity and involves mixing the compound or potential extract with DPPH solution and recording the absorbance at the wavelength 515 nm after a radical–substrate reaction time of 30 min, compared to ethanol [71,72]. Antioxidant activity was evaluated based on the IC50 value (the concentration of a compound/extract with antioxidant action that reduces by 50% the activity of free radical). The calibration curve was obtained using vitamin C solution in the concentration range between 2–22 µg/mL, with ethanol as solvent. Inhibition (%) of DPPH radical activity was calculated according to the following formula [73]:

DPPH I (%) = ((Absorbance of control − Absorbance test compounds)/Absorbance of control) × 100

For all three methods, based on the values obtained, percent inhibition values (µg/mL) were calculated, inhibition curves were constructed as a function of concentration, and using the corresponding linear equations, the IC50 values (µg/mL) were determined for each extract. The concentration range for *Betulae extractum*, *Liquiritiae extractum,* and *Avenae extractum* used in each in vitro method for antioxidant activity evaluation are presented in Supplementary Material Table S13.

### 4.8. Molecular Docking Simulations

Molecular docking simulations were performed to predict the potential of identified phytochemicals to inhibit Keap1 and stimulate Nrf2 signaling pathway. Both noncovalent and covalent docking experiments were carried out to investigate binding possibilities at both the Keap1-Nrf2 interaction site and the key reactive cysteines.

The RCSB PDB database was used to retrieve the crystal structures of target proteins. For noncovalent docking, the crystal structure of the Kelch domain of human Keap1 in the complex with small fragment inhibitor chromone-2-carboxylic acid (4-oxo-4H-1-benzopyran-2-carboxylic acid) was used (PDB ID: 5WHO, 2.23 Å resolution [27]). For covalent docking, we retrieved several crystal structures, depending on the target cysteine: the Kelch domain of Keap1 in an open, unliganded conformation for docking at Cys434 (PDB ID: 5WFL, 1.93 Å resolution [27]), the unliganded BTB domain of Keap1 for docking at cysteines 77 and 171 (PDB ID: 4CXI, 2.35 Å resolution), and the BTB domain in the complex with the covalent triterpenoid inhibitor bardoxolone for docking at Cys151 (PDB ID: 4CXT, 2.66 Å resolution [25]).

Target structures were further prepared for docking simulations with the YASARA Structure [74], as follows: solvent molecules and ions were removed, structural errors were corrected, and missing residues were added. The structure was protonated according to the physiological pH (7.4), and the hydrogen-bonding network was optimized. The retrieved protein structures were then energetically minimized using the NOVA2 forcefield.

The docking protocols were validated by extracting the co-crystallized ligands and redocking them into the binding sites. Thereafter, the predicted conformations of the ligands were superposed on the experimentally determined structure, and the Root-Mean-Square Deviation (RMSD) values were calculated. Ligands used for validation also served as positive controls [75,76]. Since there are no available solved structures of covalent inhibitors bound at residues Cys77, Cys171, and Cys434, we docked known covalent ligands found in the literature, which served as positive controls for these particular reactive sites. Therefore, for Cys77 in the BTB domain and Cys434 in the Kelch domain, we used pubescenoside A, which was reported to bind, with high specificity, to these residues [29]. Furthermore, we used pterisolic acid B for the Cys171 residue, which was also reported to preferentially bind at this site [29]. The obtained complexes with pubescenoside A and pterisolic acid B were considered valid after optimal poses were obtained, such as when the expected binding pocket was occupied, conformations were not highly strained, and the number of unsatisfied interactions was acceptable [77].

Ligands for docking were prepared by generating the corresponding three-dimensional structures with DataWarrior 5.2.1 [78], energy minimization using MMFF94s+ forcefield, and protonation at physiological pH. The AutoDock Vina v1.1.2 algorithm [79] was used within YASARA for noncovalent docking with rigid receptor atoms, and the search space (25 × 25 × 25 Å) was set to include the interaction site between Keap1 Kelch domain and Nrf2. For covalent docking, AutoDock v4.2 was used with the Lamarckian Genetic Algorithm and flexible residues [80]. The covalent docking procedure was based on Michael addition reaction, where the cysteine residue acted as the nucleophilic donor, and the studied ligands were considered electrophilic acceptors [81]. A total of 12 docking runs were executed for each ligand.

Molecular docking results were retrieved as the binding energy (ΔG, kcal/mol) and ligand efficiency (LE, ΔG\no. of heavy atoms) of the top scoring binding pose for each docked compound. Conformations of the predicted complexes and molecular interactions were analyzed using BIOVIA Discovery Studio Visualizer (BIOVIA, Discovery Studio Visualizer, Version 17.2.0, Dassault Systèmes, 2016, San Diego, CA, USA).

### 4.9. Statistical Analysis

All statistical analyses were performed using IBM SPSS Statistics Software Version 29.0.0.0 (241) (IBM Corporation, Chicago, IL, USA). For each set of experimental data, the essential conditions for the application of statistical tests were evaluated, such as the normality of data and the homogeneity of variances. The normal distribution of the data was assessed by the Shapiro–Wilk test, the Kolmogorov-Smirnov test, and by histograms. To detect significant differences between the data groups, one-way ANOVA (single-factor ANOVA) and post hoc tests were applied for mean comparison. The Games–Howell test for unequal variances was used, depending on data distribution, sampling dispersion, and the number of studied groups. Levene’s test was used to verify the homogeneity of variances for the experimental data sets. The Welch ANOVA analysis was run as a robust test when the condition of homogeneity of variances was violated. The correlation between certain groups of analyzed experimental data was also established by calculating the Pearson correlation coefficient. Interpretations were made after the mandatory application criteria were met (continuity of variables, independence of measurements, normality and linearity of data, and absence of outliers). In all cases, the significance level was set at 0.05. When *p* < 0.05, the obtained results were considered statistically significant. The slope of the calibration curve and the coefficient of determination (R^2^) were obtained by using MS Excel 2019 from Microsoft (Redmond, WA, USA).

## 5. Conclusions

The results of the present study showed that the investigated species contains a significant amount of polyphenolic compounds. These compounds are found in high concentrations in *Betulae extractum* and *Avenae extractum*, compared to *Liquiritiae* one. Additionally, *Betulae extractum* contains the highest content of total flavonoids content and is the only extract that has phenolic acids, too, which explains the good radical scavenging activity of the birch bark, in comparison with licorice root and oar herb.

The GC-MS analysis highlighted that *Betulae extractum* contains fatty acids, tannins, triterpenes, carboxylic acids, α-hydroxy carboxylic acids, sugars, phosphoric acid, butylated hydroxytoluene, 4-hexylphenol, and volatile compounds, such as sesquitujene, α-santalen, α-bergamoten, α-cucurmen, β-farnesen and bisabolen. *Liquiritiae extractum* contains fatty acids, tannins, carboxylic acids, α-hydroxy carboxylic acids, sugars, amino acids, phytoestrogenic hormone (equilin), propylenglicol, phosphoric acid, benzoic acid, aucubin, 4-aminobutanoic acid, and no volatile substances. *Avenae extractum* contains hexoses, disaccharides, carboxylic acids, fatty acids, amino acids, polyols, phenolic compounds, urea, and alpha hydroxy acids. Major compounds were also confirmed by the FT-ICR-MS analysis. For birch bark, we identified betulinic acid, betulin, betulinaldehide, betulonic acid, lupenone, lupeol, stearic acid, oleic acid, linoleic acid (fatty acids also confirmed throught GC specific identification method), betuloside, caffeic acid, kaempferol, myo-inositol; for licorice root—glycyrrhizin, glycyrrhetinic acid, liquiritigenin, isoliquiritigenin, glabridin, linoleic acid, palmitic acids (fatty acids also confirmed throught GC specific identification method), and myo-inositol; and, for oat herb, we identified avenacoside A, linoleic acid, oleic acid, palmitic acid, linolenic acid (fatty acids also confirmed throught GC specific identification method), tricin, vitexin, myo-inositol, tryptophan, citric acid, caffeic acid, ferulic acid, sucrose, D-mannose, D-glucopyranose, beta-glucan, kestose, and neokestose.

Furthermore, molecular docking simulations indicated that the phytochemicals contained in *Betula alba* var. *pendula* Roth bark extract, *Glycyrrhiza glabra* L. root extract, and *Avena sativa* L. herb extract could act as noncovalent and covalent activators of the Nrf2 signaling pathway with potential antioxidant and anti-inflammatory activities.

The study provided a basis for further investigation in the means of in vitro activity and antimicrobial potential with the purpose of developing topical formulations with impact in the improvement of various skin ailments.

## Figures and Tables

**Table 1:** The content of total flavonoid content (TFC), total polyphenol content (TPC), and total phenolic acid content (TPA).

Vegetal Extract	TFC (g Rutoside/100 g Dry Extract)	TPC (g Tannic Acid/100 g Dry Extract)	TPA (g Chlorogenic Acid/100 g Dry Extract)
*Betulae extractum* (BE)	3.747 ± 0.3140	47.96 ± 9.7083	25.34 ± 1.6728
*Liquiritiae extractum* (LE)	3.44 ± 0.3037	9.31 ± 0.9913	ND
*Avenae extractum* (AE)	1.95 ± 0.0526	40.55 ± 6.3715	ND

Results were expressed as Mean ± SD (n = 5); ND—not determined.

**Table 2:** The fatty acids identified with the GC-MS analysis in BE, LE, and AE.

*Betulae extractum* (BE)	*Liquiritiae extractum* (LE)	*Avenae extractum* (AE)
Myristic acid	Palmitic acid	Myristic acid
Palmitic acid	Linoleic acid	Palmitic acid
Linoleic acid	Oleic acid	Linoleic acid
Oleic acid	Stearic acid	Linolenic acid
Stearic acid		Stearic acid
Behenic acid		Palmitelaidic acid

**Table 3:** The quantities of linoleic acid and linolenic acid found in BE, LE, and AE.

Type of Extract	Linoleic Acid (g %)	Linolenic Acid (g %)
*Betulae extractum* (BE)	0.015	<0.01
*Liquiritiae extractum* (LE)	0.01	<0.01
*Avenae extractum* (AE)	0.19	0.92

**Table 4:** Compounds classes identified in ethanolic extracts.

Type of Extract	Compound	Compound Class
*Betulae extractum* (BE)	Betulinic acid	Triterpenes
	Oleanolic acid	
	Ursolic acid	
	Betulin	
	Erythrodiol	
	Betulinaldehide	
	Betulonic acid	
	Lupenone	
	Lupeol	
	Stearic acid	Fatty acids
	Oleic acid	
	Linoleic acid	
	Betuloside	Phenolic compounds
	Caffeic acid	
	Kaempferol	
	Myo-Inositol	Polyols
*Liquiritiae extractum* (LE)	Glycyrrhizin/Glycyrrhizinic acid	Triterpenes
	Glycyrrhetinic acid	
	Liquiritigenin/Isoliquiritigenin	Phenolic compounds
	Glabridin	
	Linoleic acid	Fatty acids
	Palmitic acid	
	Myo-inositol	Polyols
*Avenae extractum* (AE)	Avenacoside A	Steroidal saponin
	Linolenic acid	Fatty acids
	Oleic acid	
	Palmitic acid	
	Linoleic acid	
	Tricin	Flavonoids
	Vitexin	
	Myo-Inositol	Polyol
	Tryptophan	Amino acid
	Citric acid	Tricarboxilic acid
	Caffeic acid	Phenolic compounds
	Ferulic acid	
	Sucrose	Sugars
	D-mannose	
	D-glucopyranose	
	Beta-glucan	
	Kestose	
	Neokestose	

**Table 5:** Antioxidant activity of *Betulae extractum*, *Liquiritiae extractum,* and *Avenae extractum*.

Vegetal Extract	DPPH Method, IC50 (µg/mL)	ABTS Method, IC50 (µg/mL)	FRAP Method, EC50 (µg/mL)
*Betulae extractum* (BE)	73.6	11.2	58.7
*Liquiritiae extractum* (LE)	805.6	92.1	722.0
*Avenae extractum* (AE)	1122.6	99.7	135.1
Vitamin C (reference substance)	16.5	-	-

**Table 6:** Multiple comparisons between groups for the ABTS, DPPH, and FRAP methods (Games-Howell Post-Hoc test—unequal variance).

(I) Vegetal Extract Group	(J) Vegetal Extract Group	Mean Difference (I-J)		
		ABTS	DPPH	FRAP
*Betulae extractum* (BE)	*Liquiritiae*	0.0010	0.2258 *	1.0506 *
	*Avenae*	0.3240 *	0.3842 *	0.7373 *
*Avenae extractum* (AE)	*Betulae*	−0.3240 *	−0.2258 *	−1.0506 *
	*Liquiritiae*	−0.3229 *	0.1584 *	−0.3132 *
*Liquiritiae extractum* (LE)	*Betulae*	−0.0010	−0.3842 *	−0.7373 *
	*Avenae*	0.3229 *	−0.1584 *	0.3132 *

* The mean difference is significant at the 0.05 level.

## Data Availability

Not applicable.

## Supplementary Materials

The following supporting information can be downloaded at: https://www.mdpi.com/article/10.3390/plants12132510/s1, Figure S1. Betulinic acid/Oleanolic acid/Ursolic acid (C_30_H_48_O_3_)—m/z is 457.36, ESI positive; Figure S2. Betulin/Erythrodiol (C_30_H_50_O)—m/z is 443.38, ESI positive; Figure S3. Betulinaldehide (C_30_H_48_O_2_)—m/z is 441.37, ESI positive; Figure S4. Betulonic acid (C_30_H_46_O_3_)—m/z is 455.35, ESI positive; Figure S5. Lupenone (C_30_H_48_O)—m/z is 425.37, ESI positive; Figure S6. Lupeol (C_30_H_50_O)—m/z is 427.39, ESI positive; Figure S7. Stearic acid (C_18_H_36_O_2_)—m/z is 285.27, ESI positive; Figure S8. Betuloside (C_16_H_24_O_7_)—m/z is 329.15, ESI positive; Figure S9. Betulinic acid/ Oleanolic acid/Ursolic acid (C_30_H_48_O_3_)—m/z is 455.35, ESI negative; Figure S10. Betulonic acid (C_30_H_46_O_3_)—m/z is 453.33, ESI negative; Figure S11. Betuloside (C_16_H_24_O_7_)—m/z is 327.14, ESI negative; Figure S12. Caffeic acid (C_9_H_8_O_4_)—m/z is 179.03, ESI negative; Figure S13. Kaempferol (C_15_H_10_O_5_)—m/z is 285.04, ESI negative; Figure S14. Linoleic acid (C_18_H_32_O_2_)—m/z is 279.23, ESI negative; Figure S15. Oleic acid (C_18_H_34_O_2_)—m/z is 281.24, ESI negative; Figure S16. Stearic acid (C_18_H_36_O_2_)—m/z is 283.26, ESI negative; Figure S17. Myo-inositol (C_6_H_12_O_6_)—m/z is 179.05, ESI negative; Figure S18. Glycyrrhizin/Glycyrrhizinic acid (C_42_H_62_O_16_)—m/z is 823.41, ESI positive; Figure S19. Glycyrrhetinic acid (C_30_H_46_O_4_)—m/z is 471.34, ESI positive; Figure S20. Liquiritigenin/Isoliquiritigenin (C_15_H_12_O_4_)—m/z is 257.08, ESI positive; Figure S21. Glabridin (C_20_H_20_O_4_)—m/z is 325.14, ESI positive; Figure S22. Palmitic acid (C_16_H_32_O_2_)—m/z is 257.24, ESI positive; Figure S23. Glycyrrhizin/Glycyrrhizinic acid (C_42_H_62_O_16_)—m/z is 821.39, ESI negative; Figure S24. Glycyrrhetinic acid (C_30_H_46_O_4_)—m/z is 469.33, ESI negative; Figure S25. Liquiritigenin/Isoliquiritigenin (C_15_H_12_O_4_)—m/z is 255.06, ESI negative; Figure S26. Glabridin (C_20_H_20_O_4_)—m/z is 323.12, ESI negative; Figure S27. Linoleic acid (C_18_H_32_O_2_)—m/z is 279.23, ESI negative; Figure S28. Palmitic acid (C_16_H_32_O_2_)—m/z is 255.23, ESI negative; Figure S29. Myo-inositol (C_6_H_12_O_6_)—m/z is 179.05, ESI negative; Figure S30. Avenacoside A (C_51_H_82_O_23_)—m/z is 1063.53, ESI positive; Figure S31. Linolenic acid (C_18_H_30_O_2_)—m/z is 279.23, ESI positive; Figure S32. Oleic acid (C_18_H_34_O_2_)—m/z is 283.26, ESI positive; Figure S33. Palmitic acid (C_16_H_32_O_2_)—m/z is 257.24, ESI positive; Figure S34. Sucrose (C_12_H_22_O_11_)—m/z is 343.12, ESI positive; Figure S35. Tryptophan (C_11_H_12_N_2_O_2_)—m/z is 205.09, ESI positive; Figure S36. Tricin (C_17_H_14_O_7_)—m/z is 331.08, ESI positive; Figure S37. Vitexin (C_21_H_20_O_10_)—m/z is 433.11, ESI positive; Figure S38. Linolenic acid (C_18_H_30_O_2_)—m/z is 277.21, ESI negative; Figure S39. Oleic acid (C_18_H_34_O_2_)—m/z is 281.24, ESI negative; Figure S40. Palmitic acid (C_16_H_32_O_2_)—m/z is 255.23, ESI negative; Figure S41. Linoleic acid (C_18_H_32_O_2_)—m/z is 279.23, ESI negative; Figure S42. Sucrose (C_12_H_22_O_11_)—m/z is 341.10, ESI negative; Figure S43. Myo-Inositol/D-mannose/D-glucopyranose (C_6_H_12_O_6_)—m/z is 179.05, ESI negative; Figure S44. Beta-glucan/Kestose/Neokestose (C_18_H_32_O_16_)—m/z is 503.16, ESI negative; Figure S45. Tricin (C_17_H_14_O_7_)—m/z is 329.06, ESI negative; Figure S46. Vitexin C_21_H_20_O_10_)—m/z is 431.09, ESI negative; Figure S47. Caffeic acid (C_9_H_8_O_4_)—m/z is 179.03, ESI negative; Figure S48. Ferulic acid (C_10_H_10_O_4_)—m/z is 193.05, ESI negative; Figure S49. Citric acid (C_6_H_8_O_7_)—m/z is 191.01, ESI negative; Table S1: Calculated mass and measured mass for some compounds from *Betulae extractum*, *Liquiritiae extractum* and *Avenae extractum* identified by FT-ICR analysis; Figure S50. Rutoside standard calibration curve; Figure S51. Tannic acid standard calibration curve; Figure S52. Chlorogenic acid standard calibration curve; Figure S53. Ascorbic acid standard curve; Table S2. The compounds identified with the GC-MS analysis in the *Betulae extractum* (BE), grouped in classes; Table S3. Compounds identified with GC-MS analysis for *Betulae extractum*; Table S4. The compounds identified with the GC-MS analysis in the *Liquiritiae extractum* (LE), grouped in classes; Table S5. Compounds identified with GC-MS analysis for *Liquiritiae extractum*; Table S6. Compounds identified with the GC-MS analysis for *Avenae extractum* (AE), grouped in classes; Table S7. Compounds identified with GC-MS analysis for *Avenae extractum*; Table S8. Multiple comparisons between groups for ABTS method (Games-Howell Post-Hoc Test—unequal variances); Table S9. Multiple comparisons between groups for DPPH method (Games-Howell Post-Hoc Test—unequal variances); Table S10. Multiple comparisons between groups for FRAP method (Games-Howell Post-Hoc Test—unequal variances); Figure S54. Binding poses of positive controls; Table S11. Molecular docking results after simulating noncovalent interactions between phytochemicals and Keap1 Kelch domain; Table S12. Molecular docking results after simulating covalent interactions with reactive cysteines within Keap1 BTB and Kelch domains; Table S13. The concentration range for *Betulae extractum*, *Liquiritiae extractum* and *Avenae extractum* used in the in vitro methods for antioxidant activity evaluation.
