# Supplementary material for: Phytochemical Screening and Antioxidant Potential of Selected Extracts from Betula alba var. pendula Roth., Glycyrrhiza glabra L., and Avena sativa L
Source: Plants (Basel). 2023 Jun 30;12(13):2510. doi: 10.3390/plants12132510 (PMC10346185; doi:10.3390/plants12132510)
Supplement: Supplementary file 1 [file plants-12-02510-s001.zip › plants-2470623-supplementary.pdf]

Supplementary Materials

S1. Results

S1.1. FT-ICR-MS spectra

1. *Betulae extractum* (BE), ESI positive

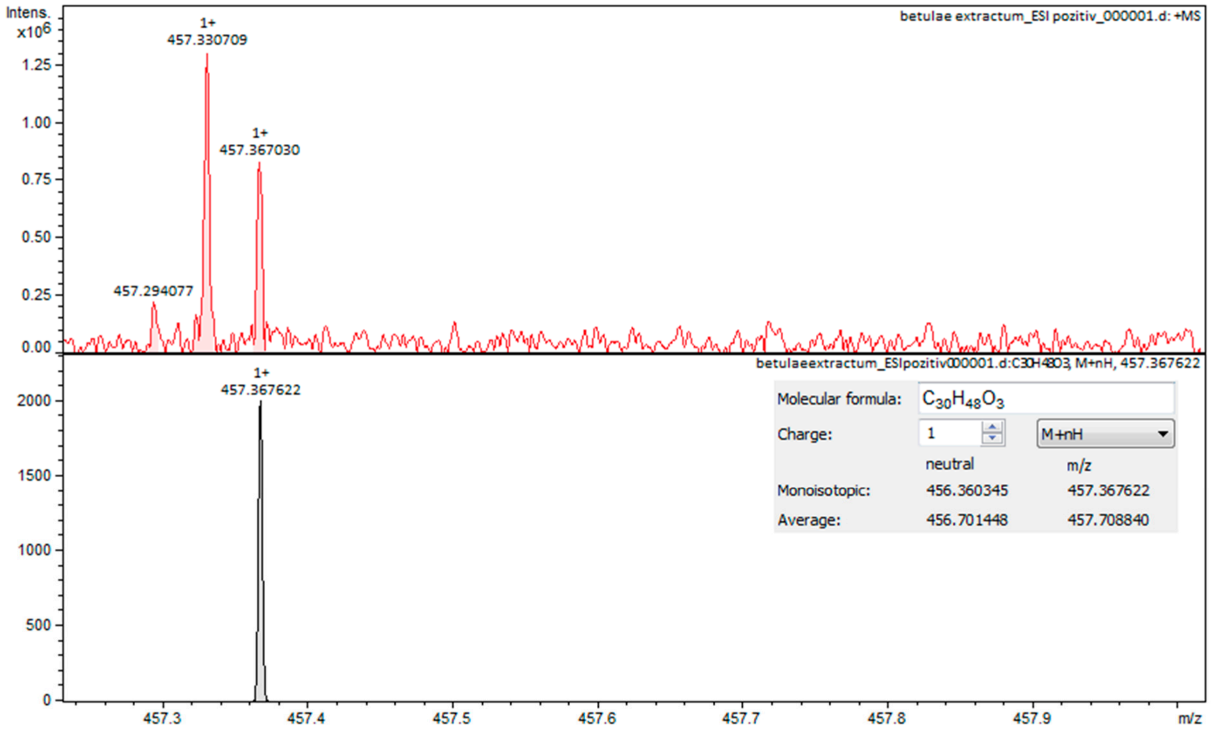

Figure S1. Betulinic acid / Oleanolic acid / Ursolic acid ($C_{30}H_{48}O_3$) – m/z is 457.36, ESI positive

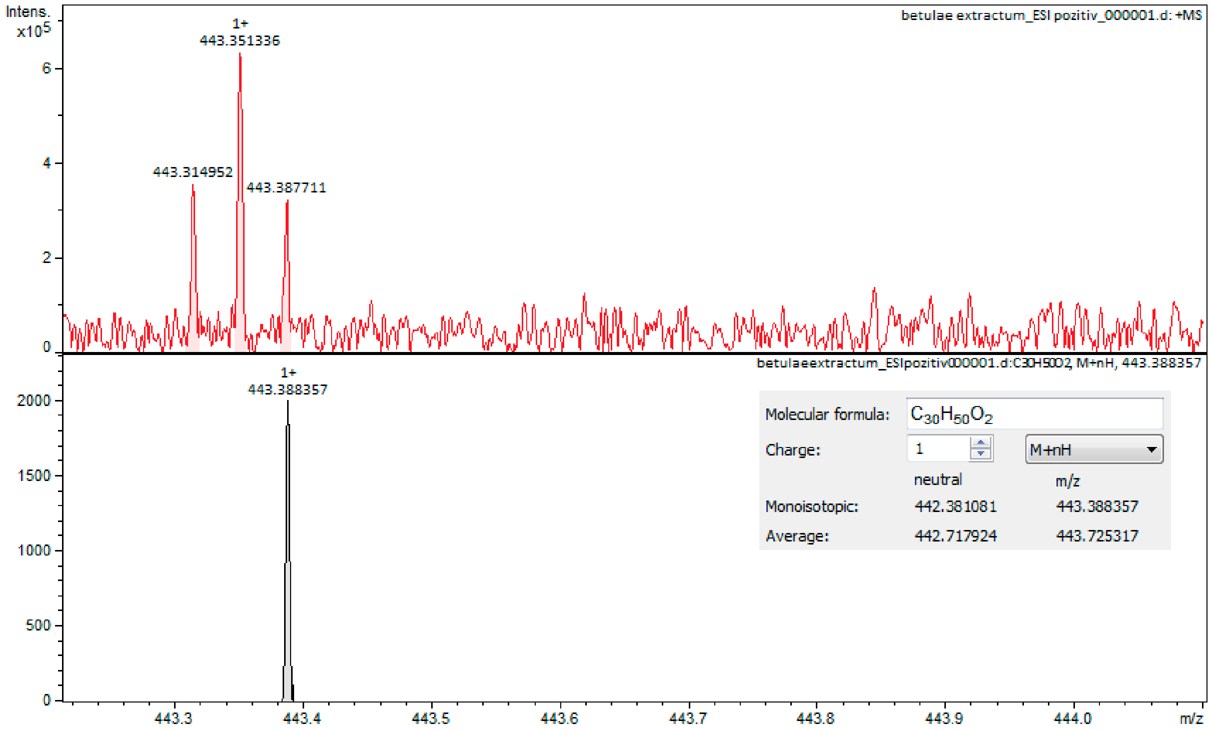

Figure S2. Betulin / Erythrodiol ($C_{30}H_{50}O$) – m/z is 443.38, ESI positive

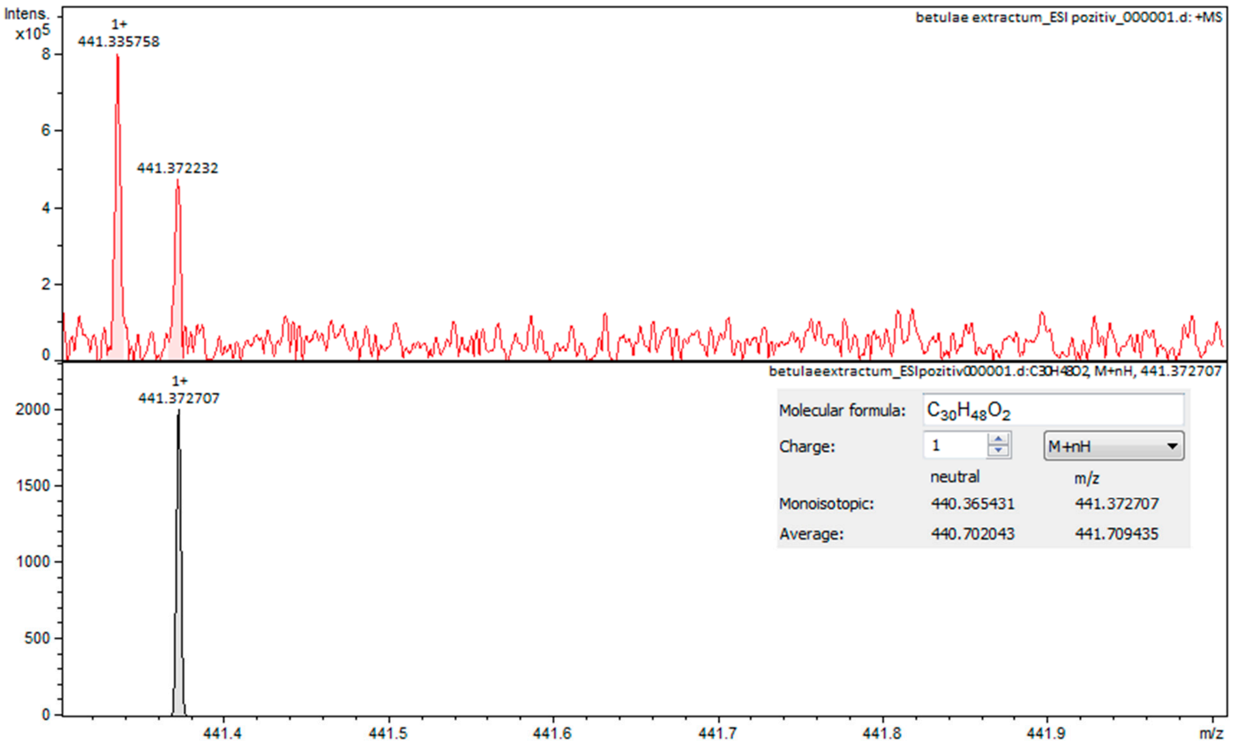

Figure S3. Betulinaldehide ($C_{30}H_{48}O_2$) – m/z is 441.37, ESI positive

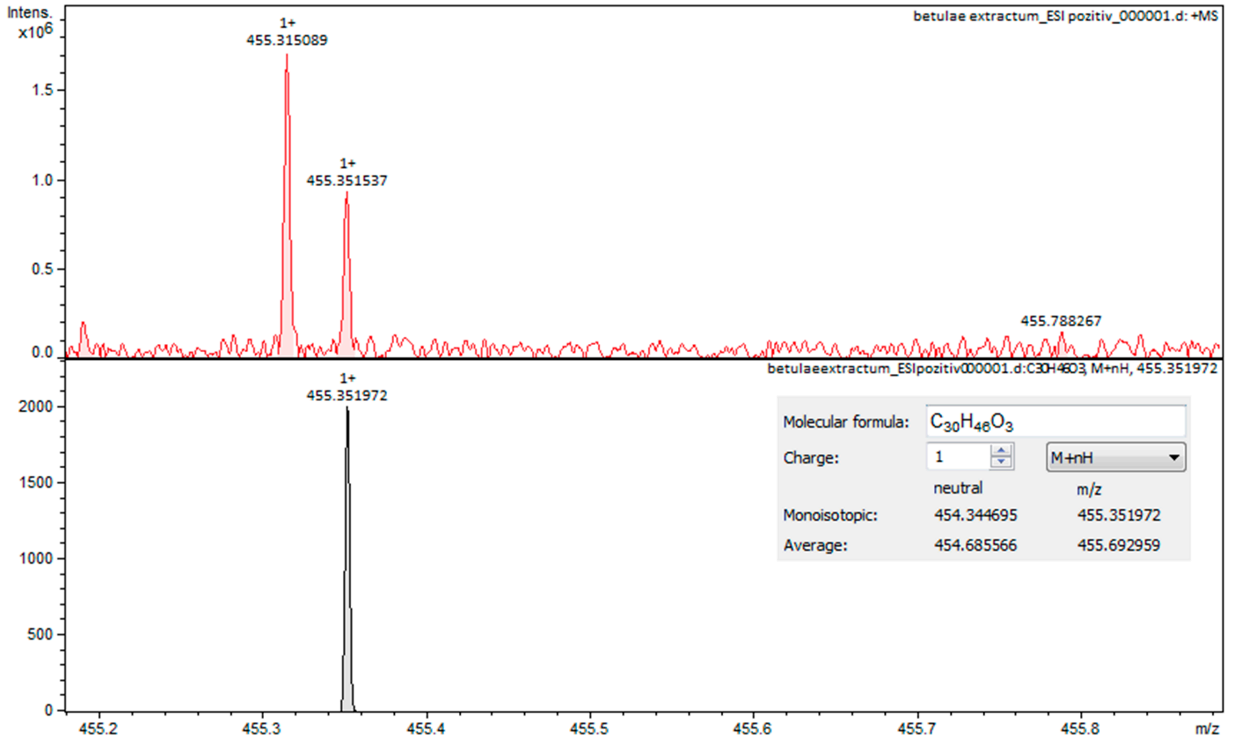

Figure S4. Betulonic acid ($C_{30}H_{46}O_3$) – m/z is 455.35, ESI positive

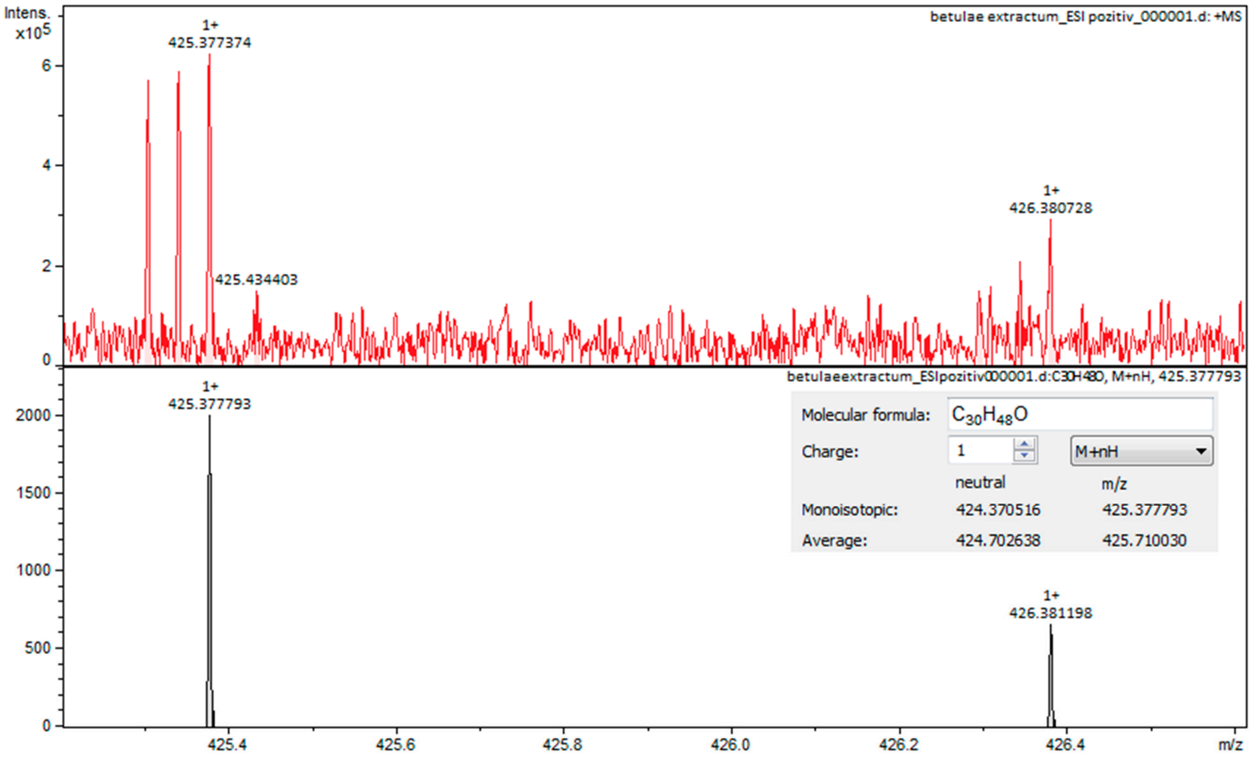

Figure S5. Lupenone ($C_{30}H_{48}O$) – m/z is 425.37, ESI positive

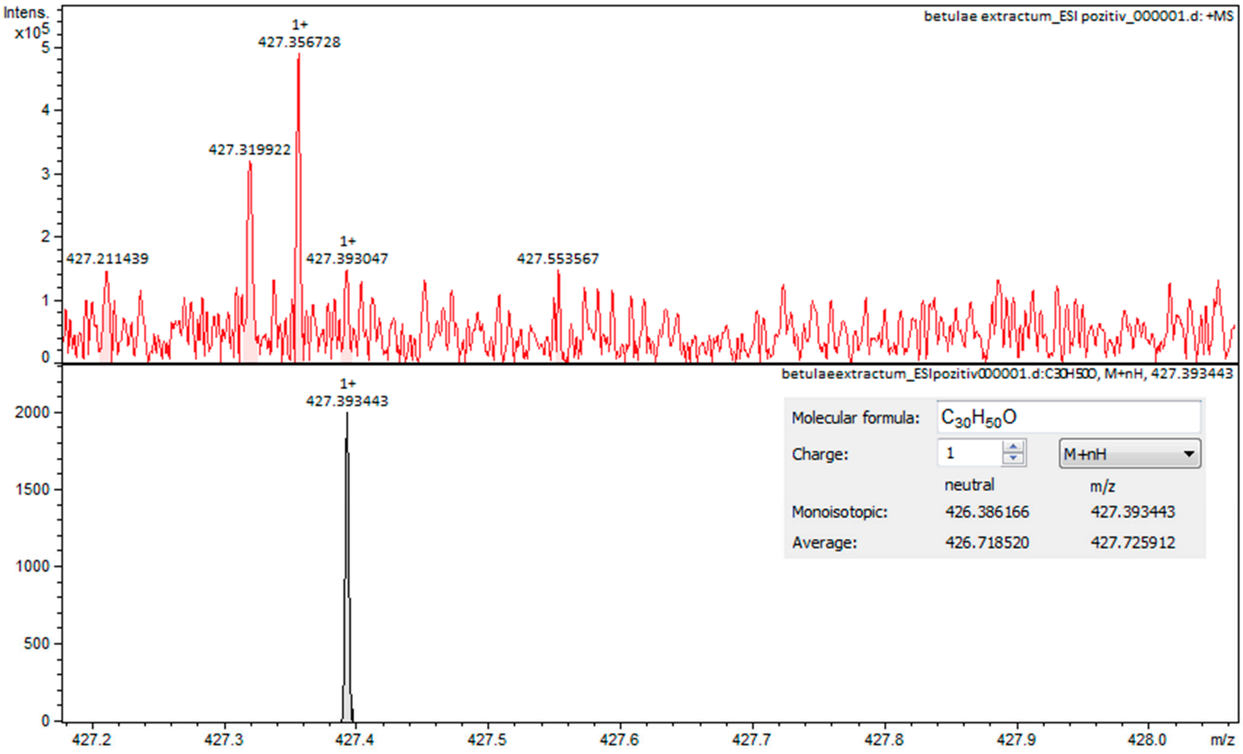

Figure S6. Lupeol ($C_{30}H_{50}O$) – m/z is 427.39, ESI positive

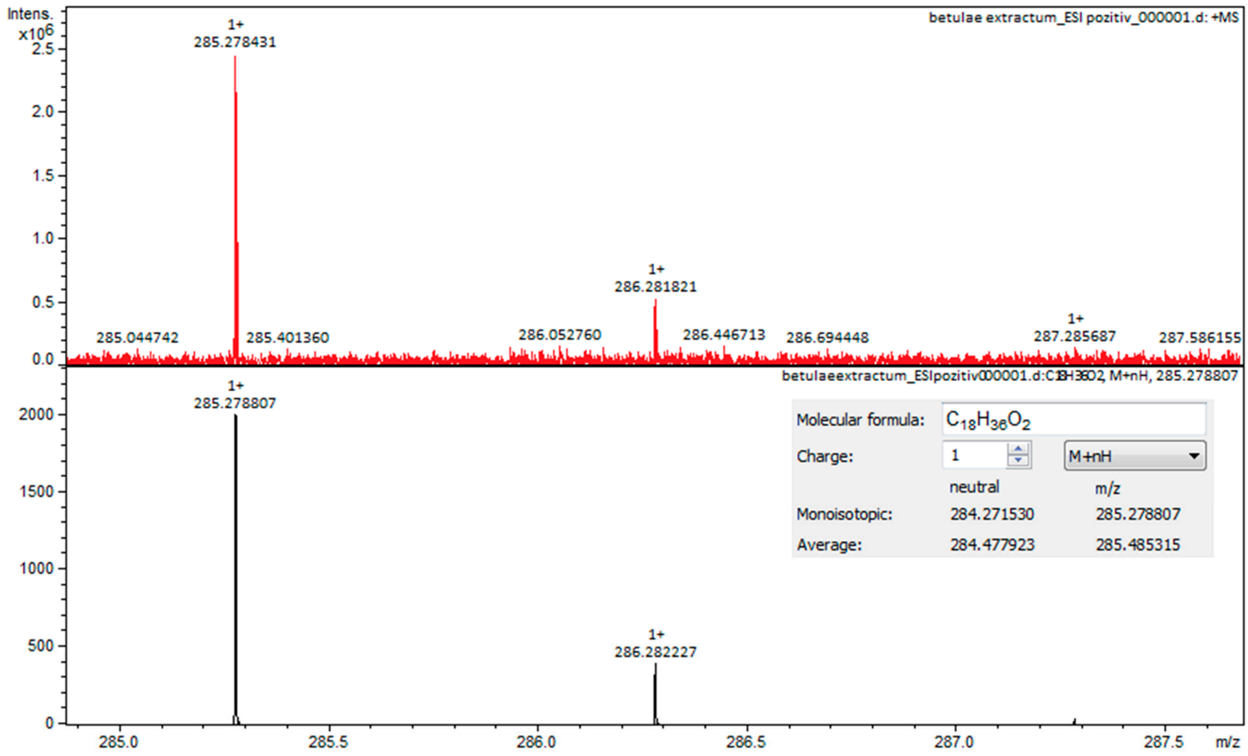

Figure S7. Stearic acid ($C_{18}H_{36}O_2$) – m/z is 285.27, ESI positive

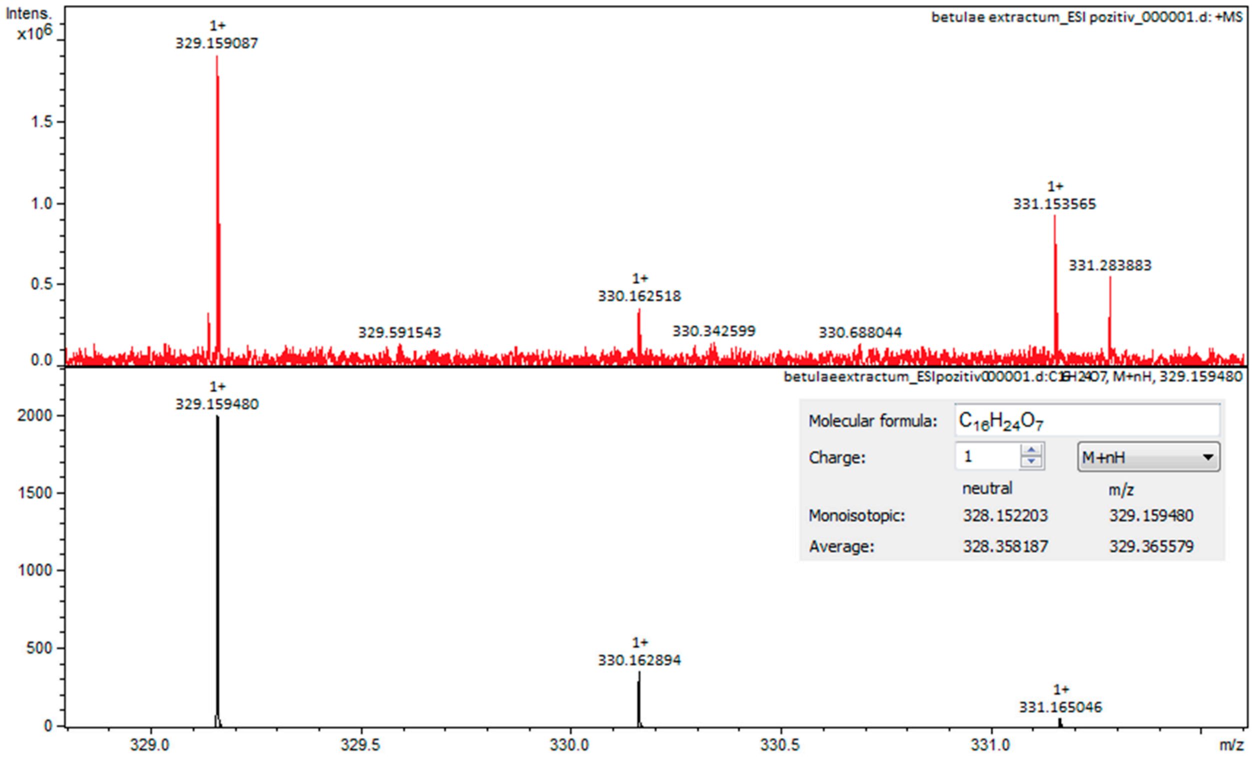

Figure S8. Betuloside ($C_{16}H_{24}O_7$) – m/z is 329.15, ESI positive

2. ***Betulae extractum*** **(BE), ESI negative**

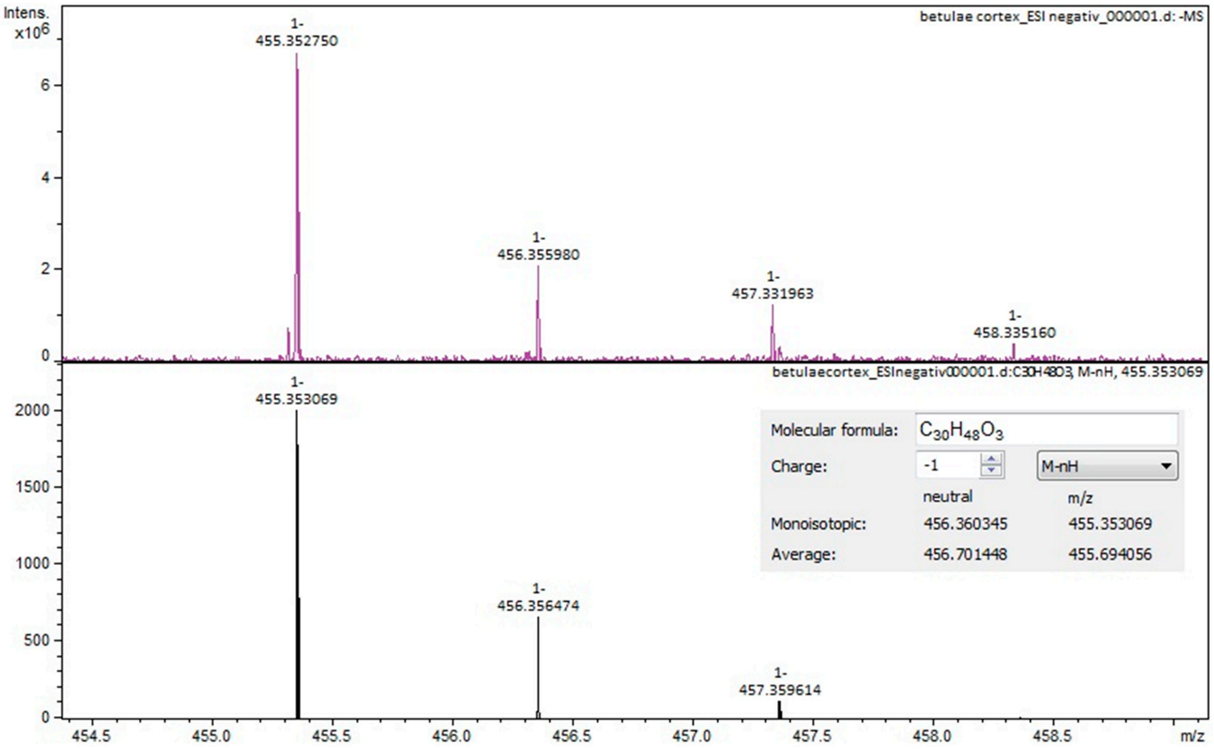

Figure S9. Betulinic acid/ Oleanolic acid / Ursolic acid ($C_{30}H_{48}O_3$) – m/z is 455.35, ESI negative

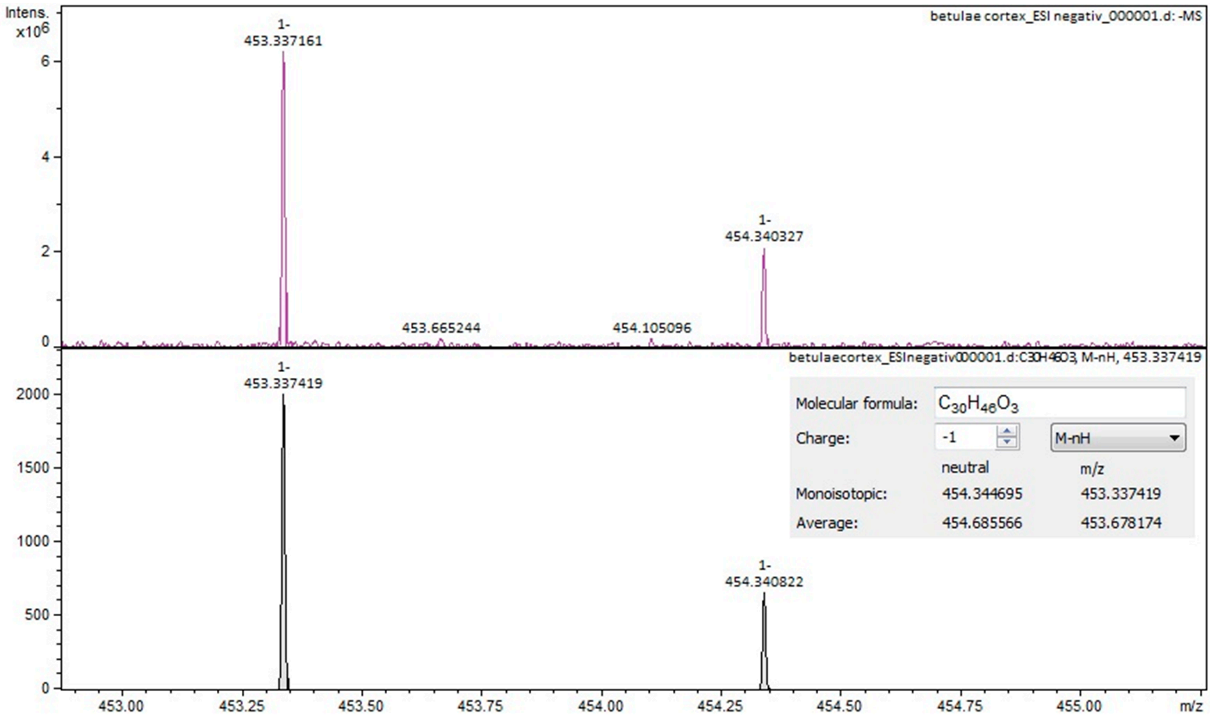

Figure S10. Betulonic acid ($C_{30}H_{46}O_3$) – m/z is 453.33, ESI negative

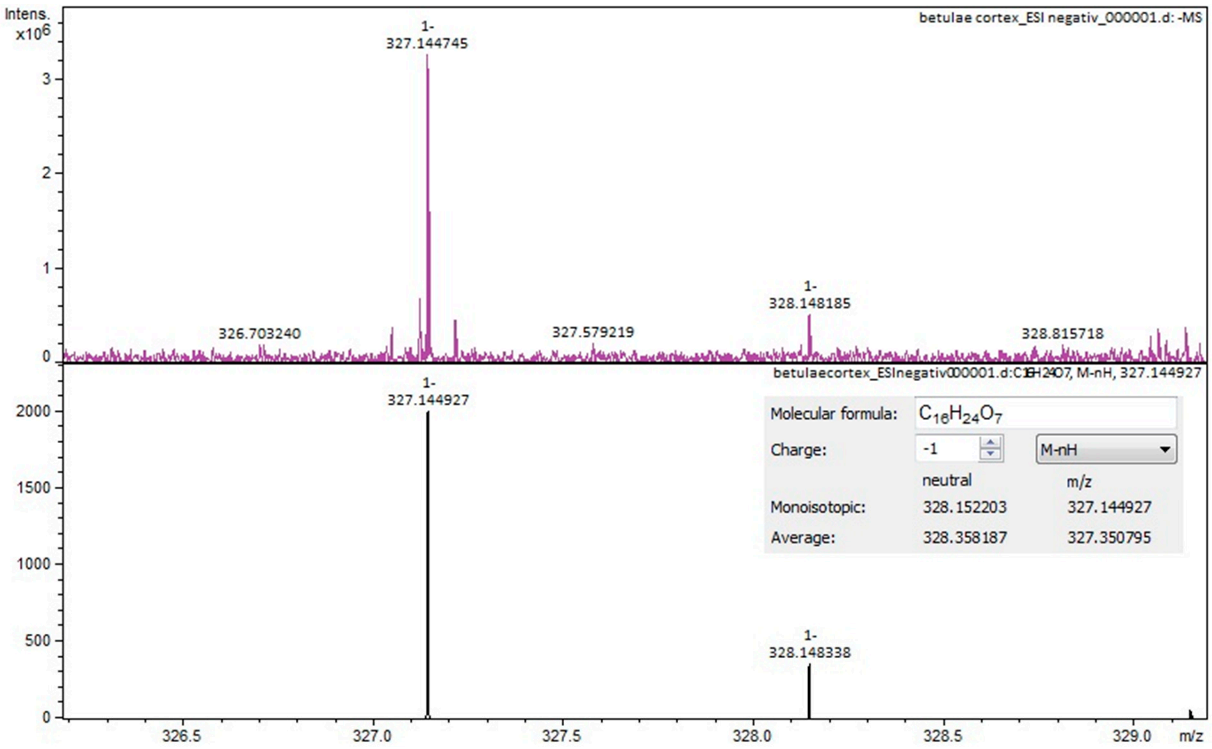

Figure S11. Betuloside ($C_{16}H_{24}O_7$) – m/z is 327.14, ESI negative

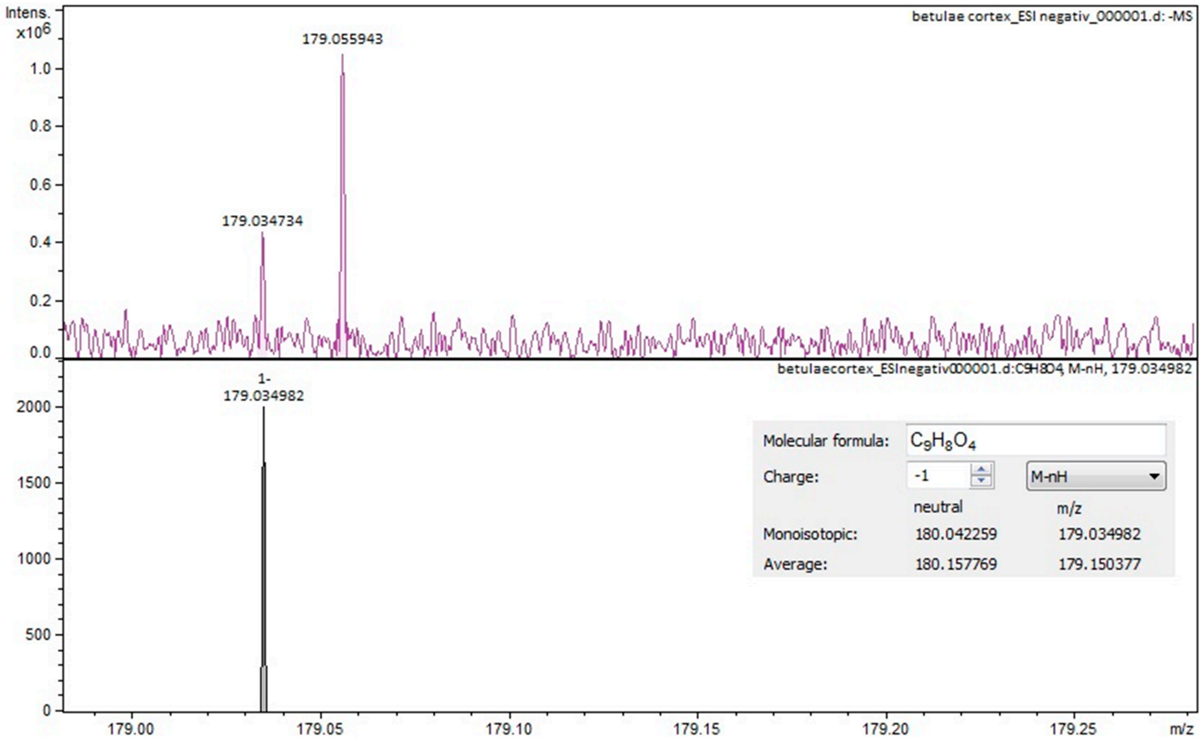

Figure S12. Caffeic acid ($C_9H_8O_4$) – m/z is 179.03, ESI negative

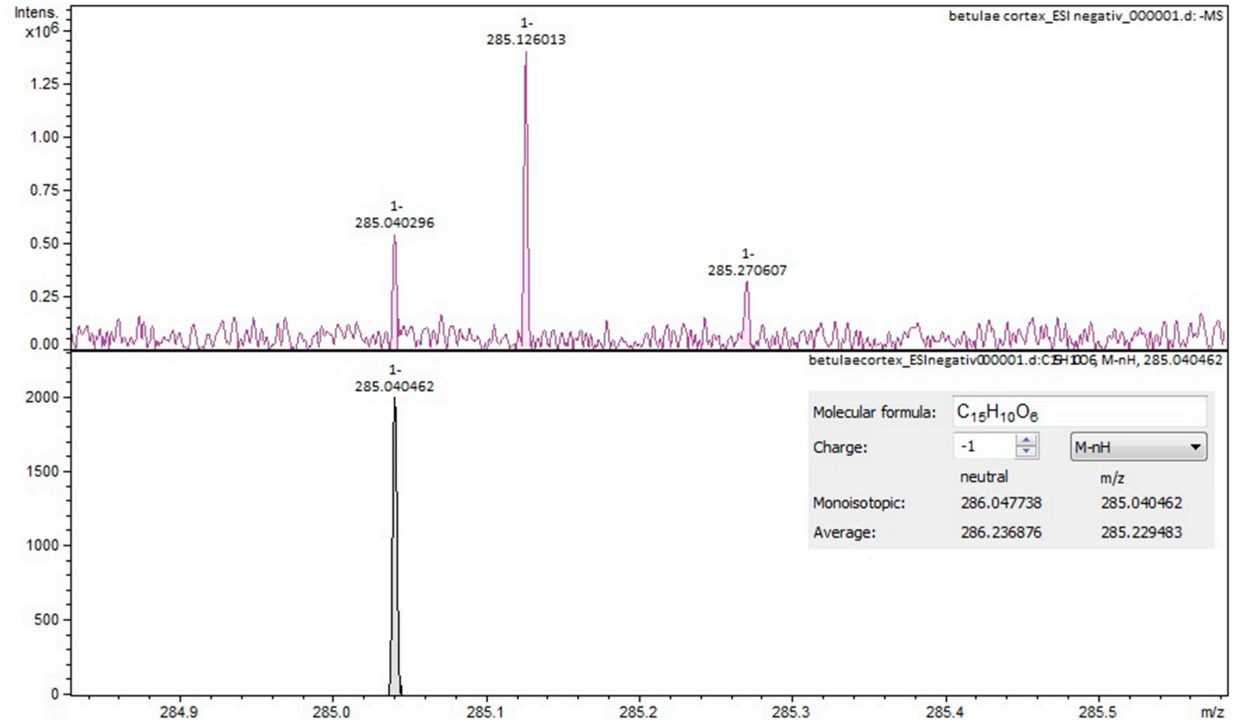

Figure S13. Kaempferol ($C_{15}H_{10}O_5$) – m/z is 285.04, ESI negative

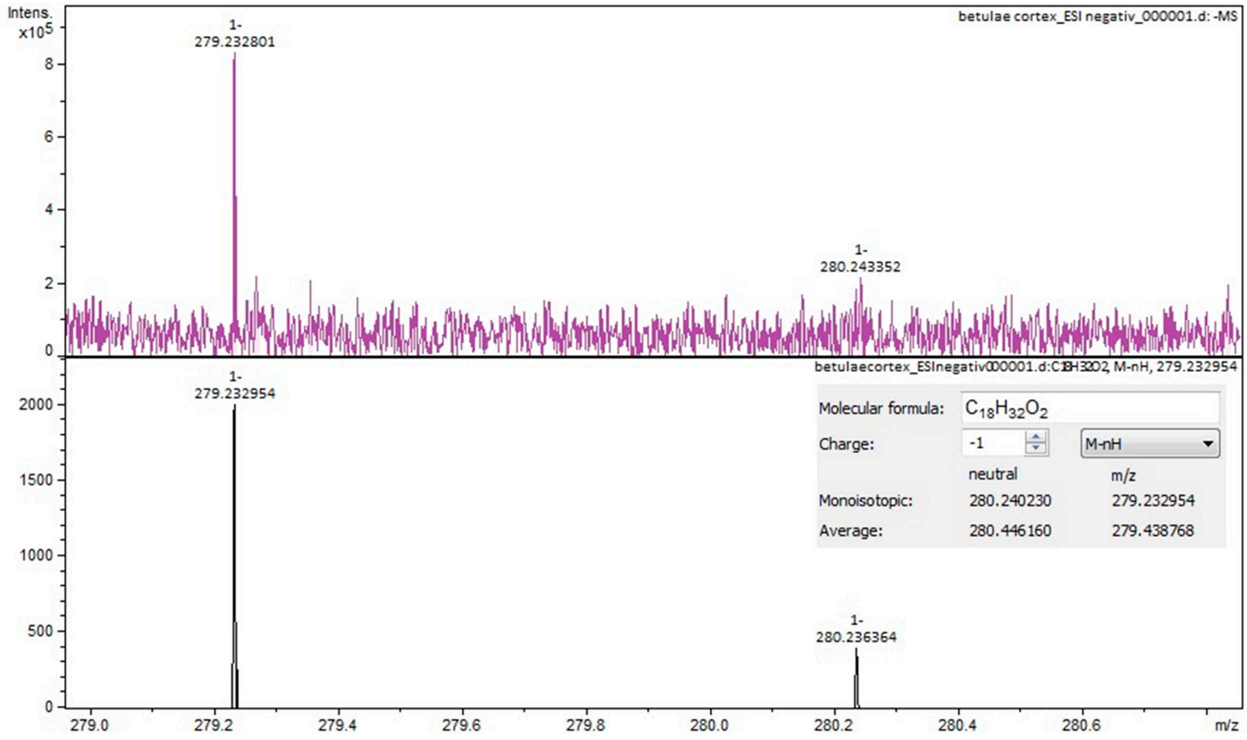

Figure S14. Linoleic acid ($C_{18}H_{32}O_2$) – m/z is 279.23, ESI negative

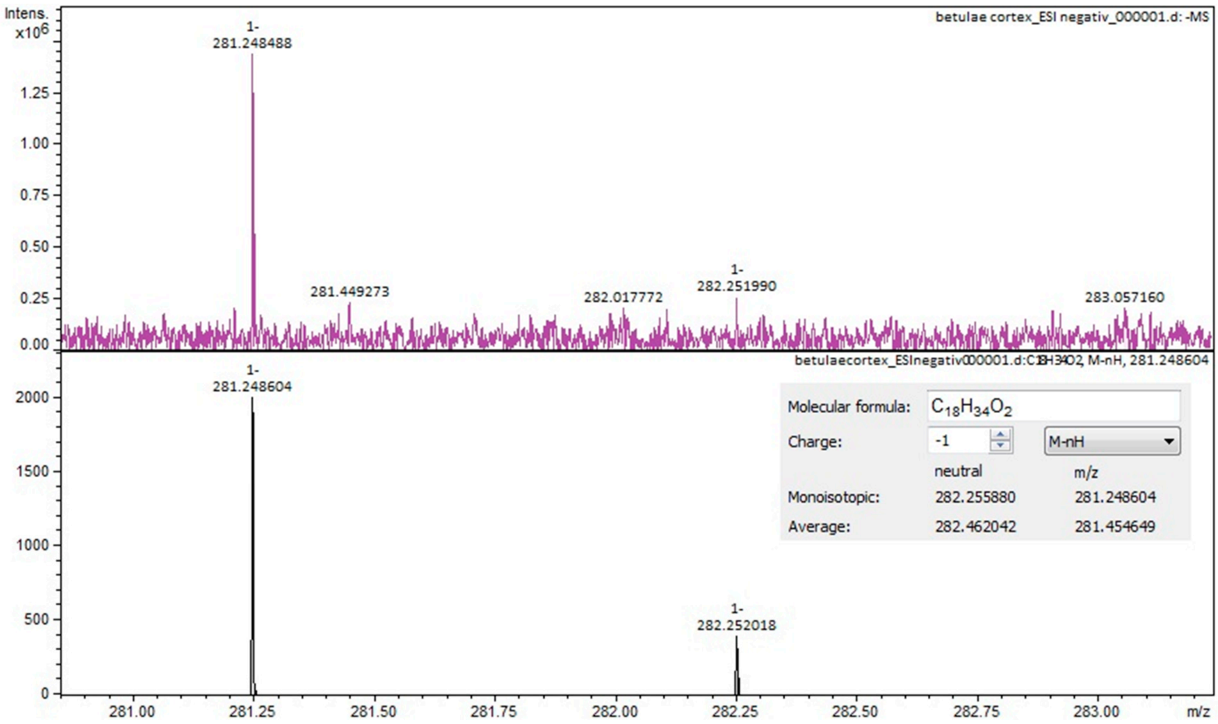

Figure S15. Oleic acid ($C_{18}H_{34}O_2$) – m/z is 281.24, ESI negative

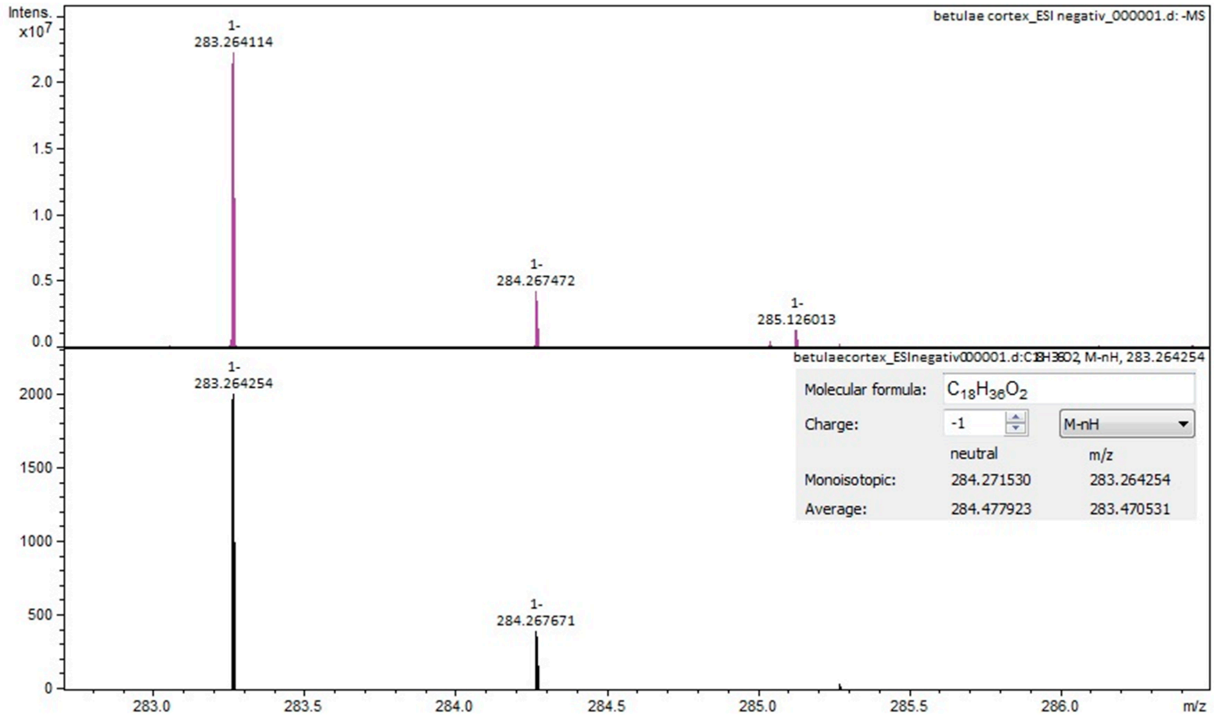

Figure S16. Stearic acid ($C_{18}H_{36}O_2$) – m/z is 283.26, ESI negative

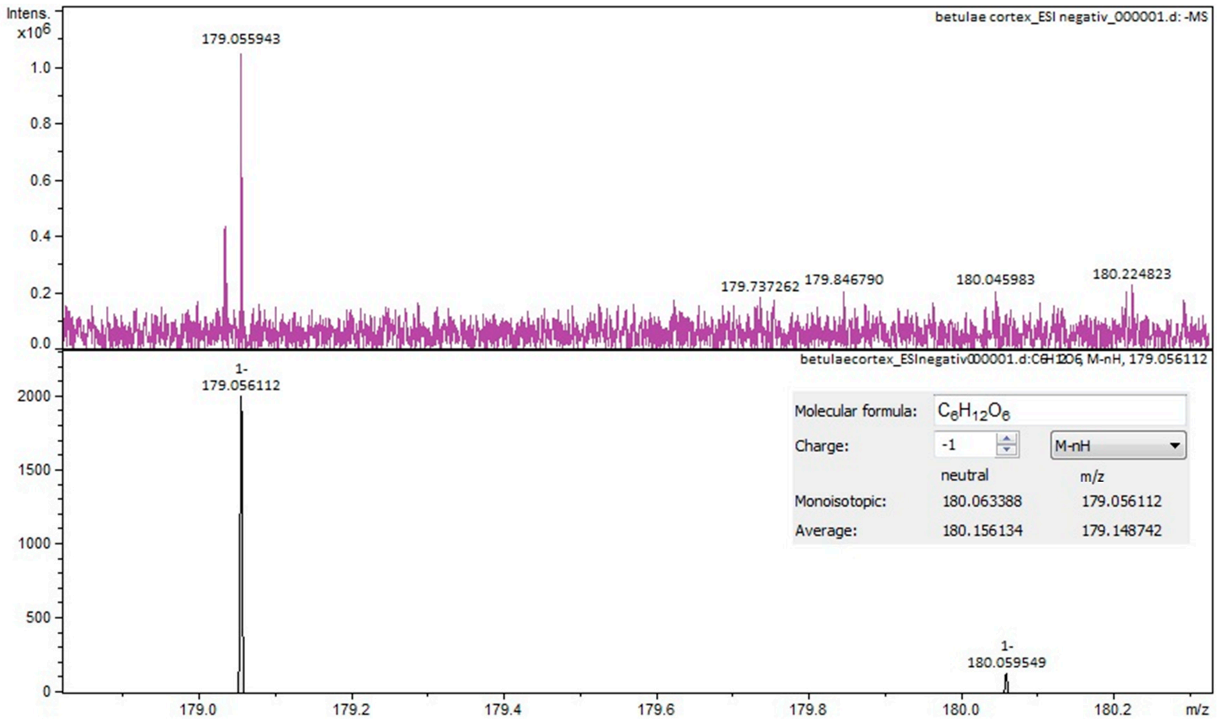

Figure S17. Myo-inositol ($C_6H_{12}O_6$) – m/z is 179.05, ESI negative

3. *Liquiritiae extractum* (LE), ESI positive

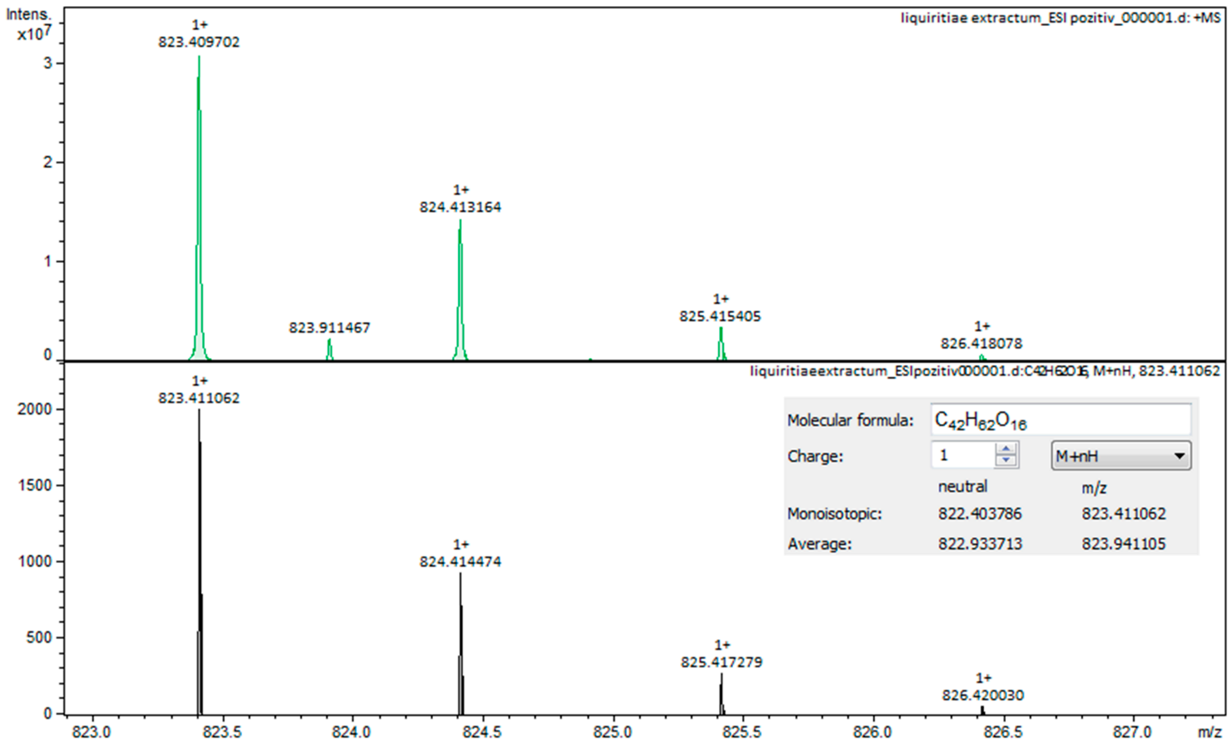

Figure S18. Glycyrrhizin / Glycyrrhizinic acid ($C_{42}H_{62}O_{16}$) – m/z is 823.41, ESI positive

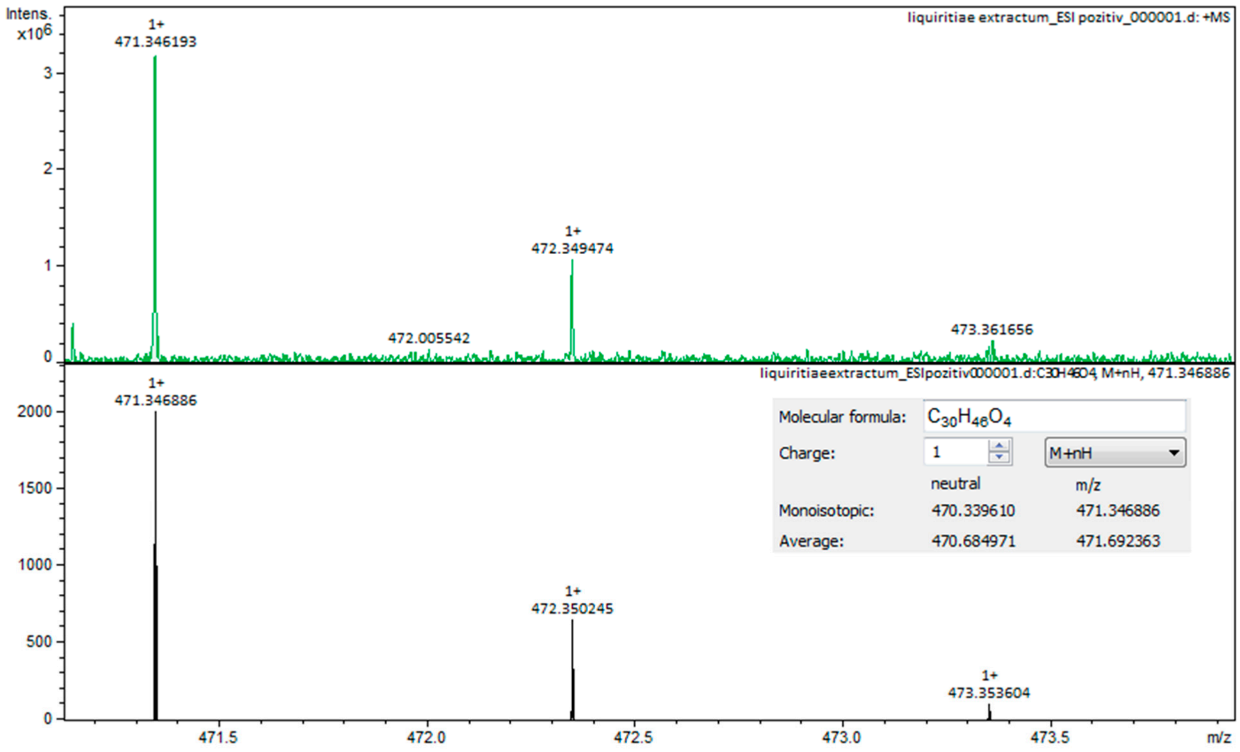

Figure S19. Glycyrrhetinic acid ($C_{30}H_{46}O_4$) – m/z is 471.34, ESI positive

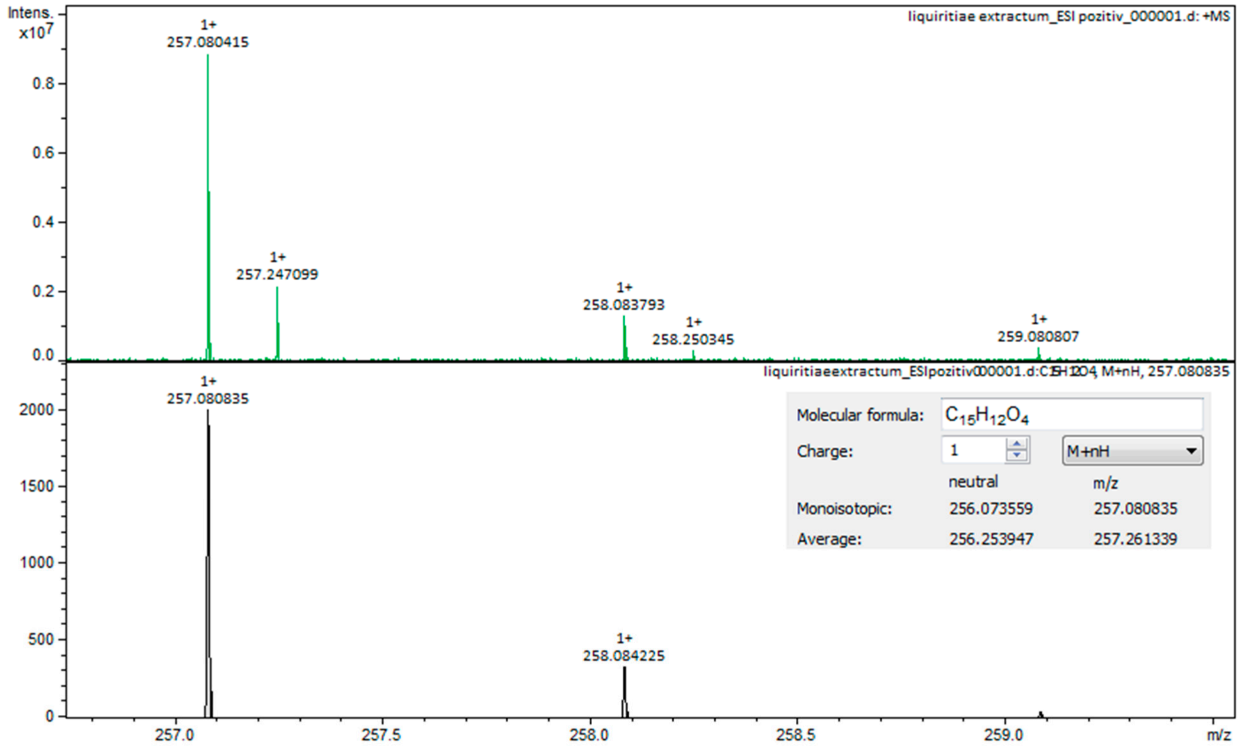

Figure S20. Liquiritigenin / Isoliquiritigenin ($C_{15}H_{12}O_4$) – m/z is 257.08, ESI positive

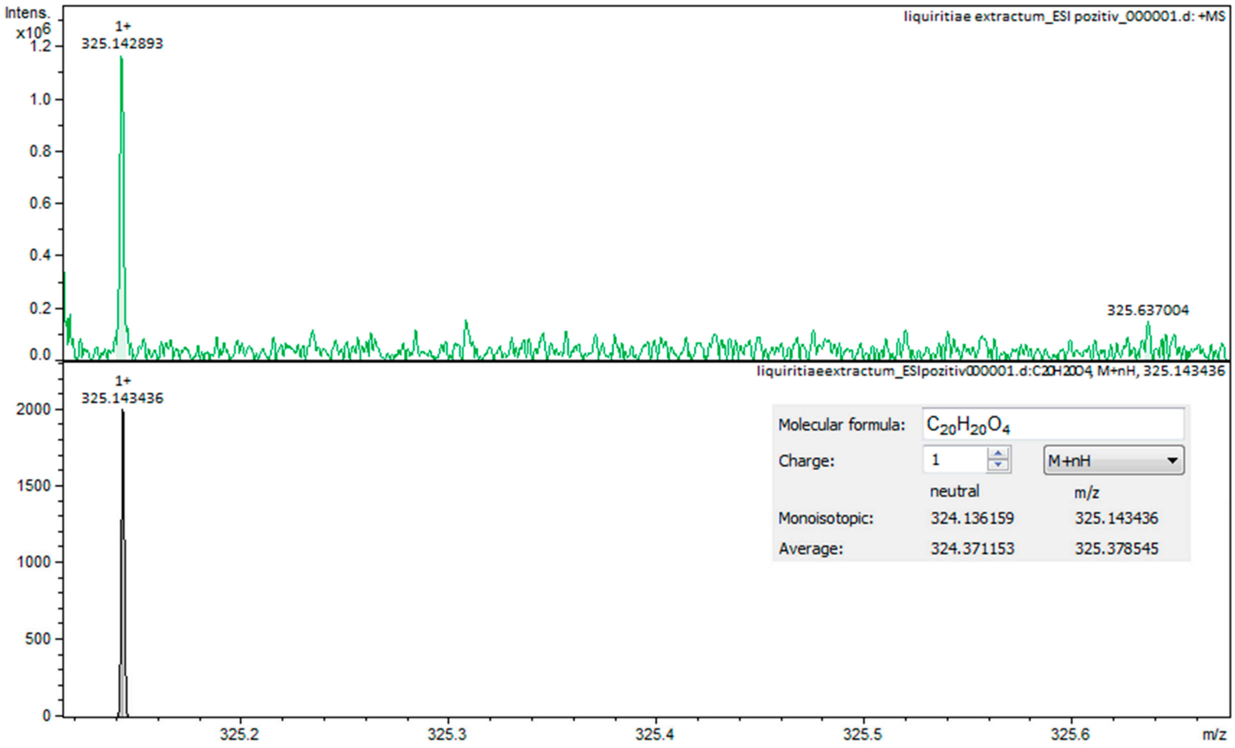

Figure S21. Glabridin ($C_{20}H_{20}O_4$) – m/z is 325.14, ESI positive

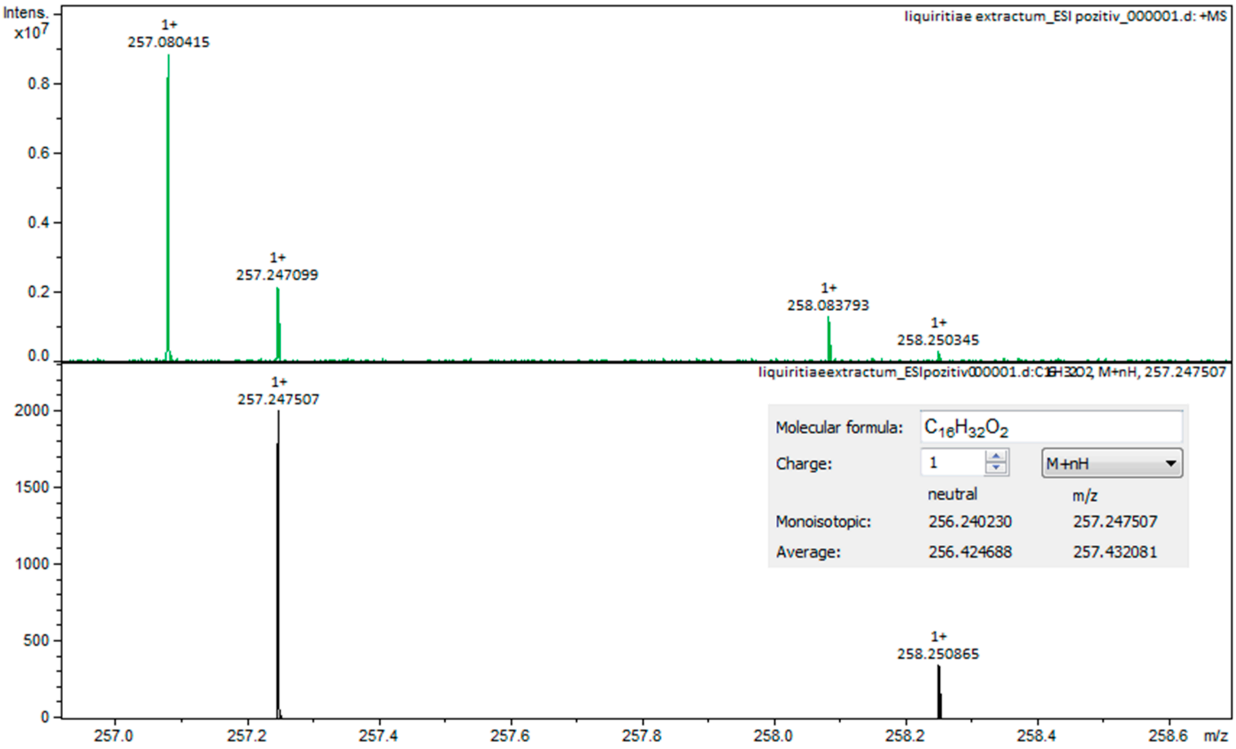

Figure S22. Palmitic acid ($C_{16}H_{32}O_2$) – m/z is 257.24, ESI positive

4. *Liquiritiae extractum* (LE), ESI negative

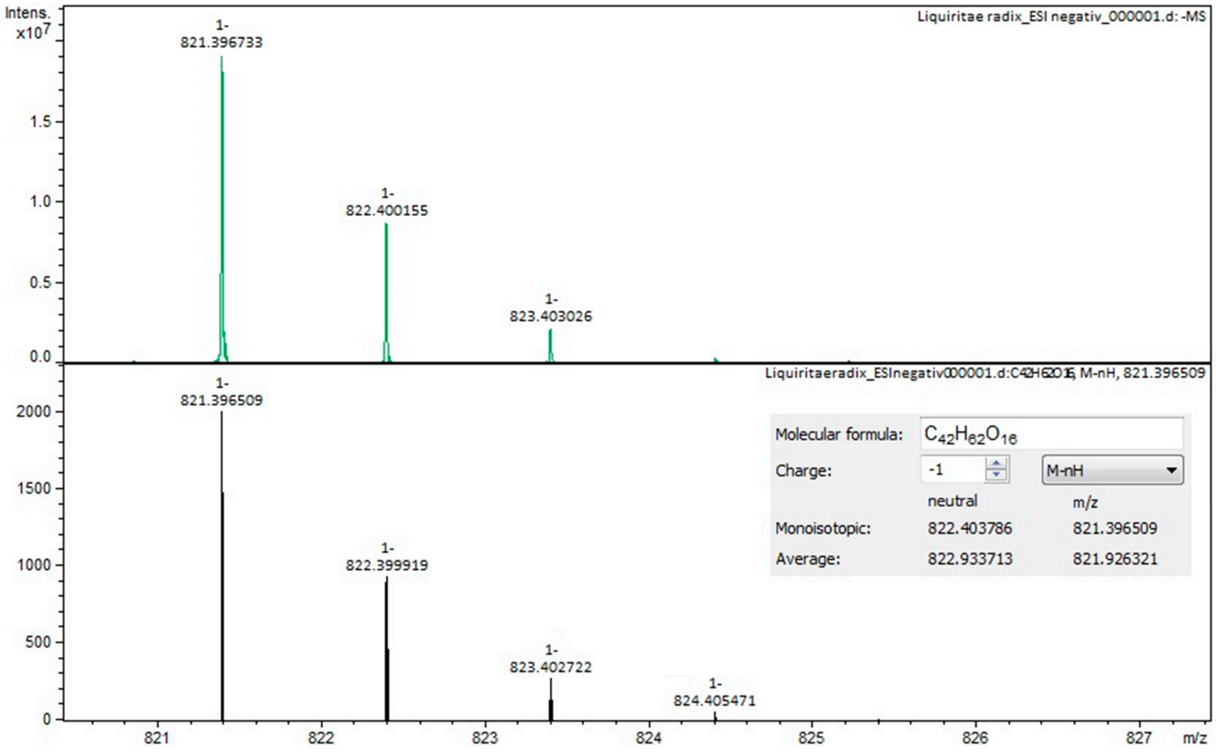

Figure S23. Glycyrrhizin / Glycyrrhizinic acid ($C_{42}H_{62}O_{16}$) – m/z is 821.39, ESI negative

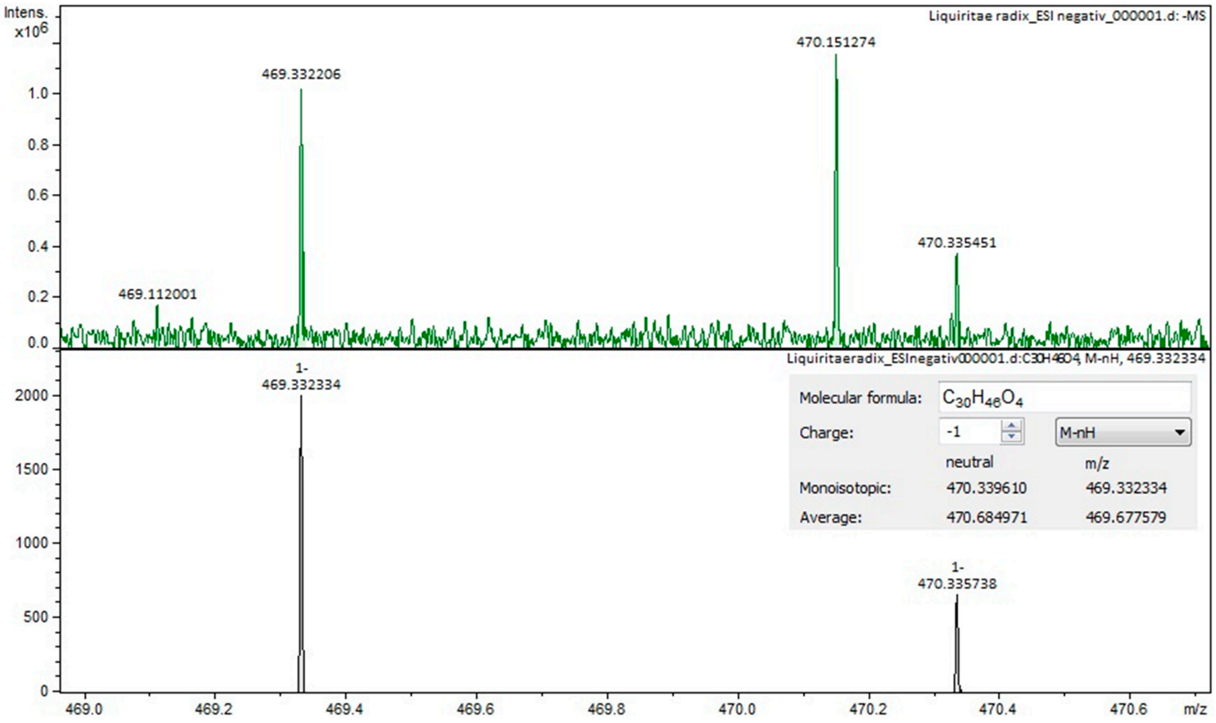

Figure S24. Glycyrrhetinic acid ($C_{30}H_{46}O_4$) – m/z is 469.33, ESI negative

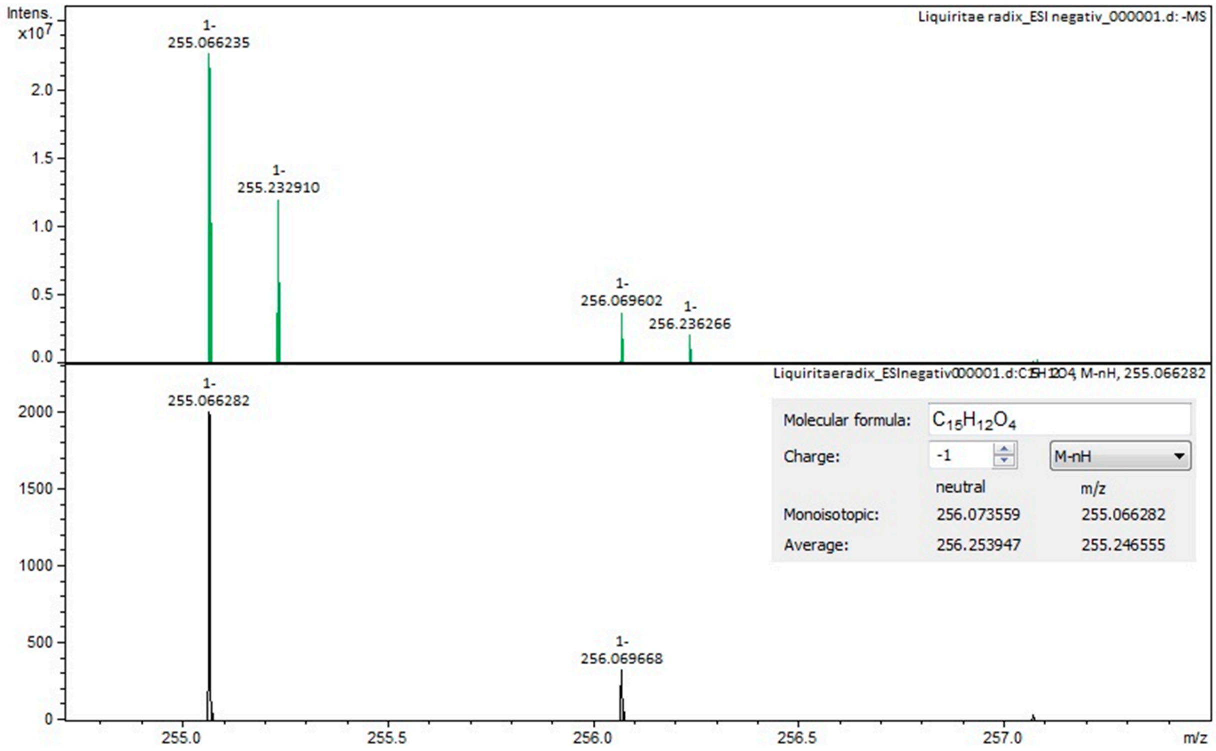

Figure S25. Liquiritigenin / Isoliquiritigenin ($C_{15}H_{12}O_4$) – m/z is 255.06, ESI negative

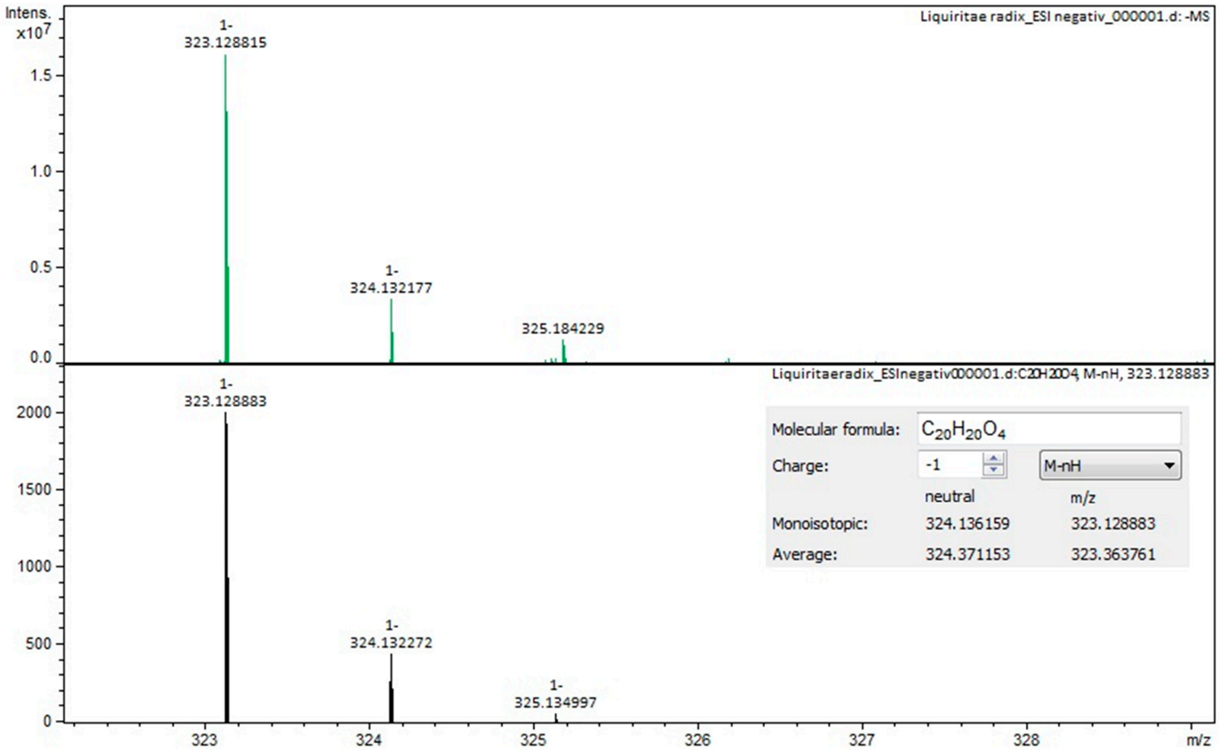

Figure S26. Glabridin ($C_{20}H_{20}O_4$) – m/z is 323.12, ESI negative

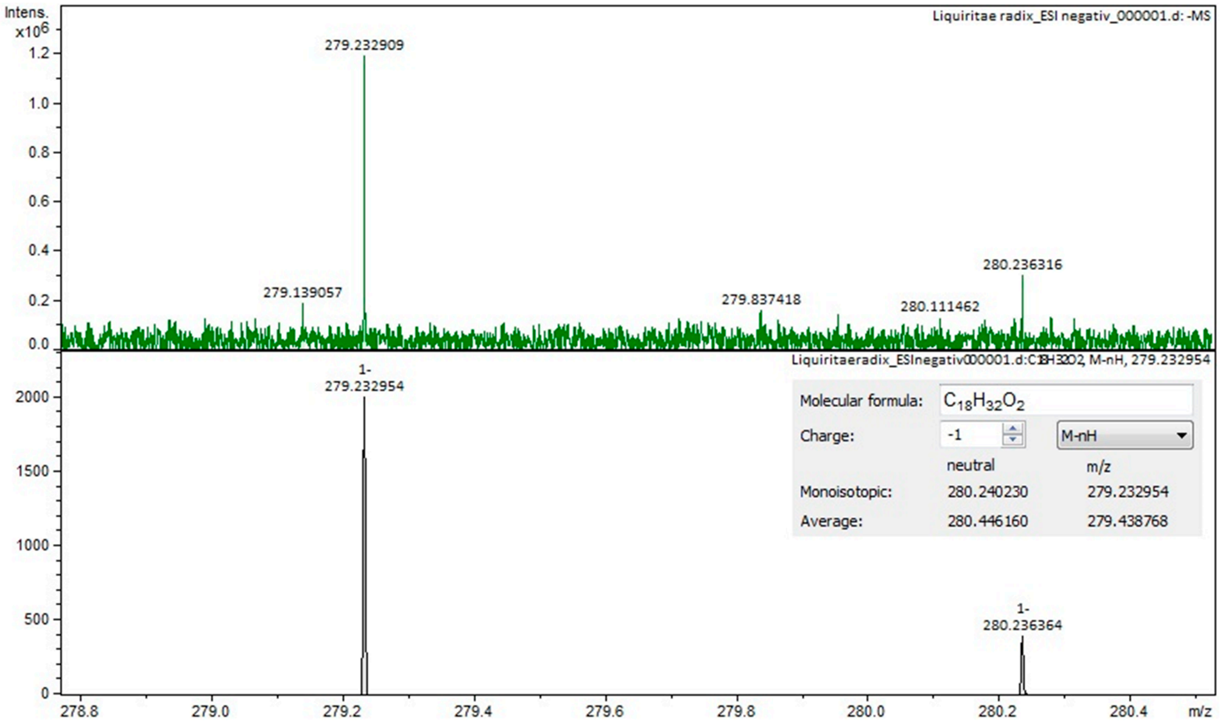

Figure S27. Linoleic acid ($C_{18}H_{32}O_2$) – m/z is 279.23, ESI negative

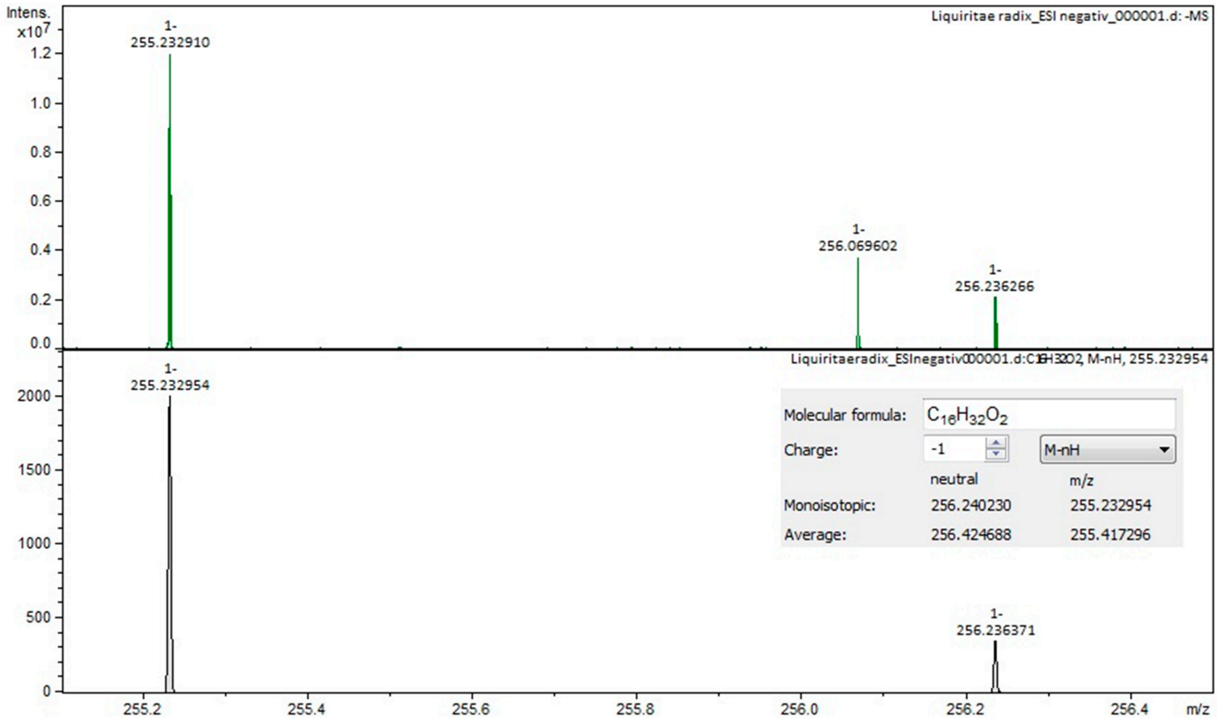

Figure S28. Palmitic acid ($C_{16}H_{32}O_2$) – m/z is 255.23, ESI negative

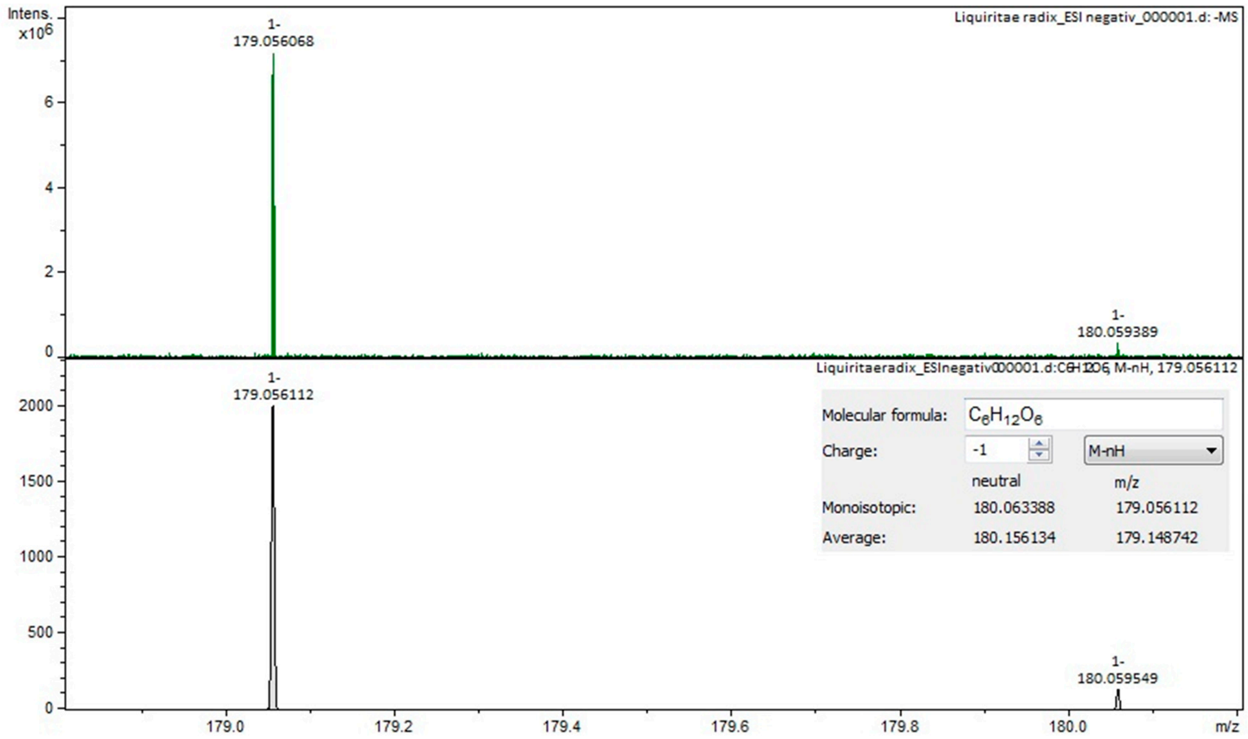

Figure S29. Myo-inositol ($C_6H_{12}O_6$) – m/z is 179.05, ESI negative

5. *Avenae extractum* (AE), ESI positive

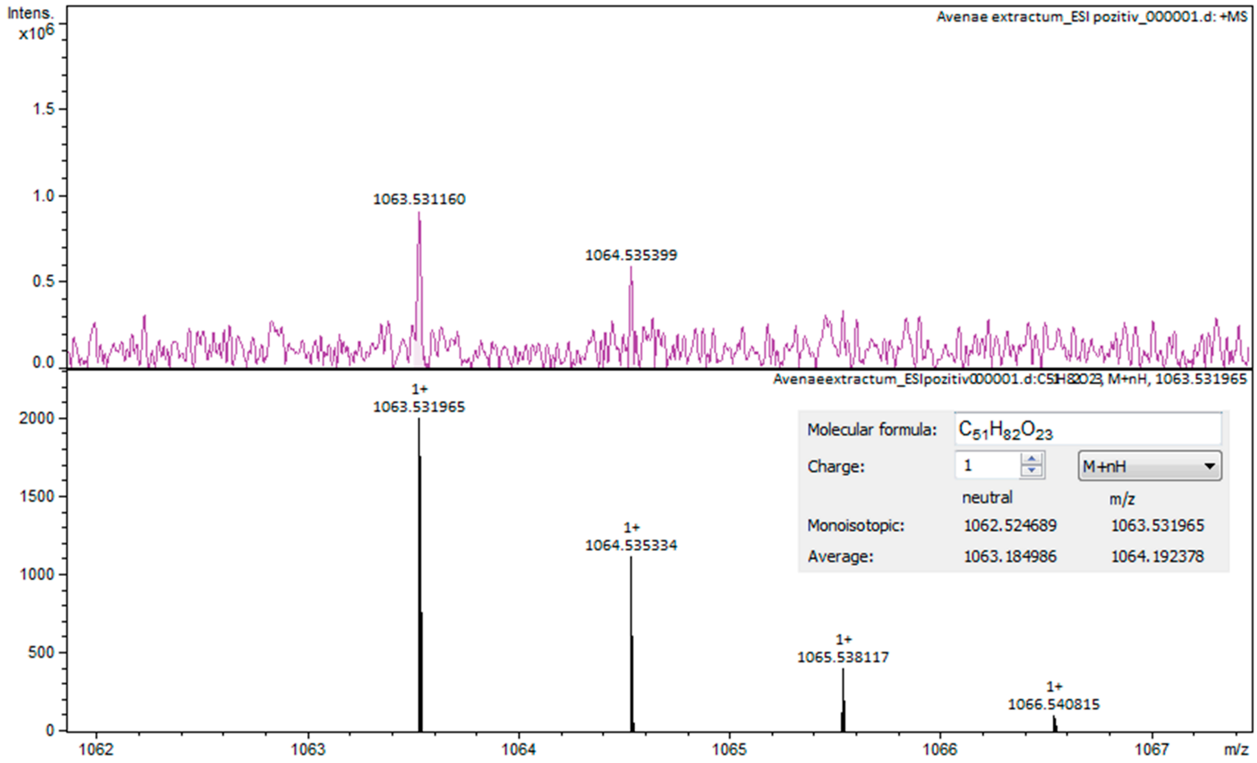

Figure S30. Avenacoside A ($C_{51}H_{82}O_{23}$) – m/z is 1063.53, ESI positive

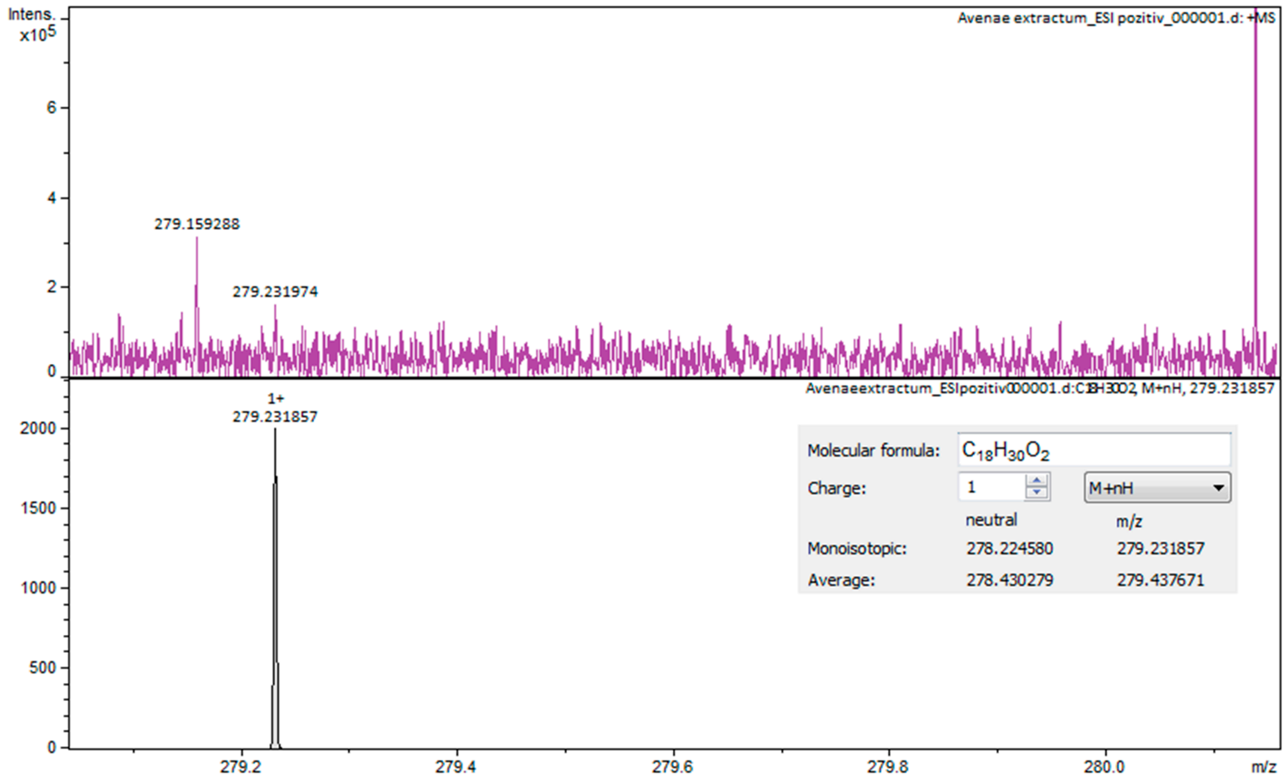

Figure S31. Linolenic acid ($C_{18}H_{30}O_2$) – m/z is 279.23, ESI positive

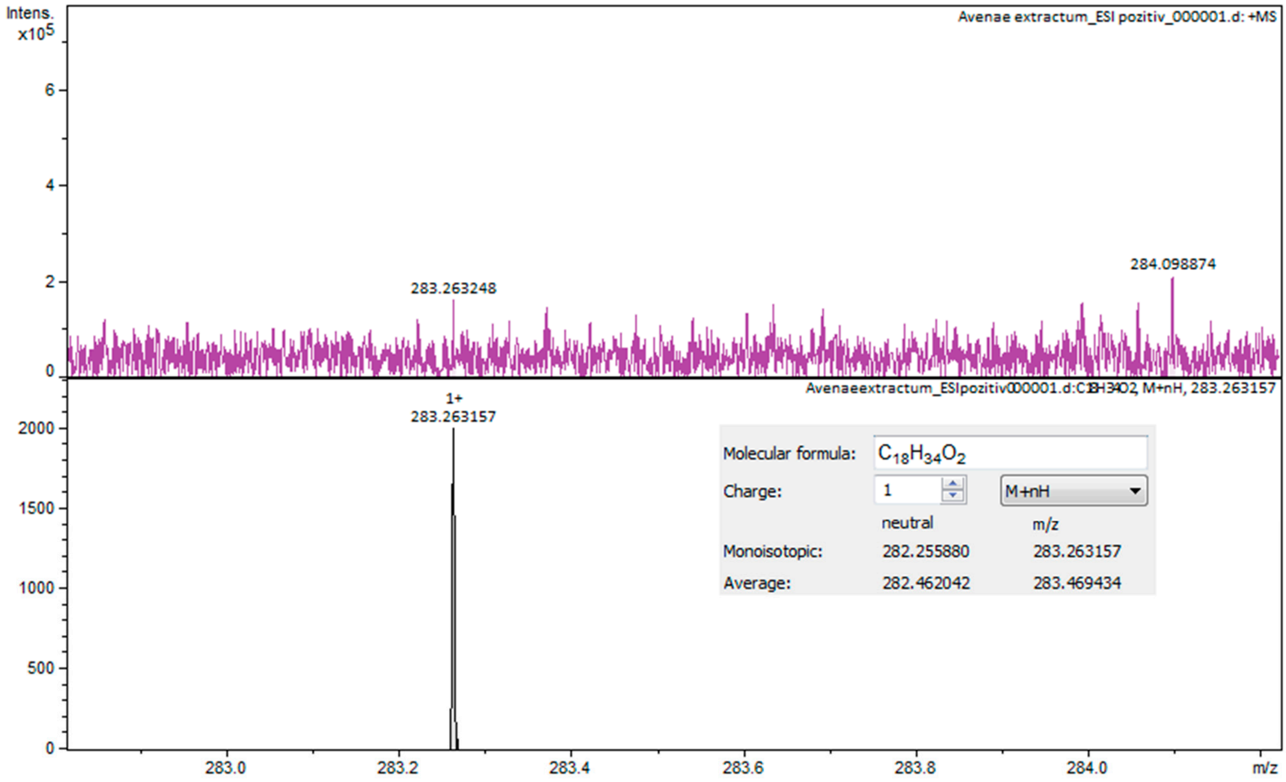

Figure S32. Oleic acid ($C_{18}H_{34}O_2$) – m/z is 283.26, ESI positive

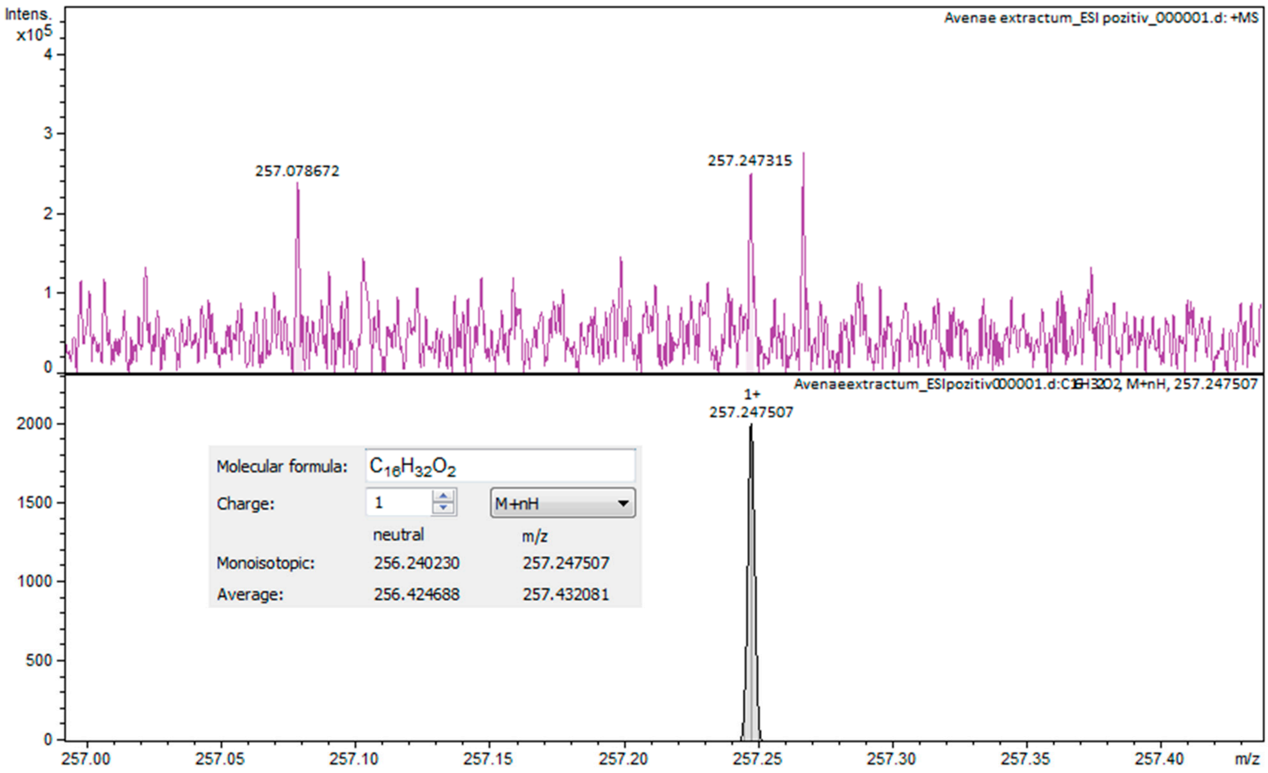

Figure S33. Palmitic acid (C16H32O2) – m/z is 257.24, ESI positive

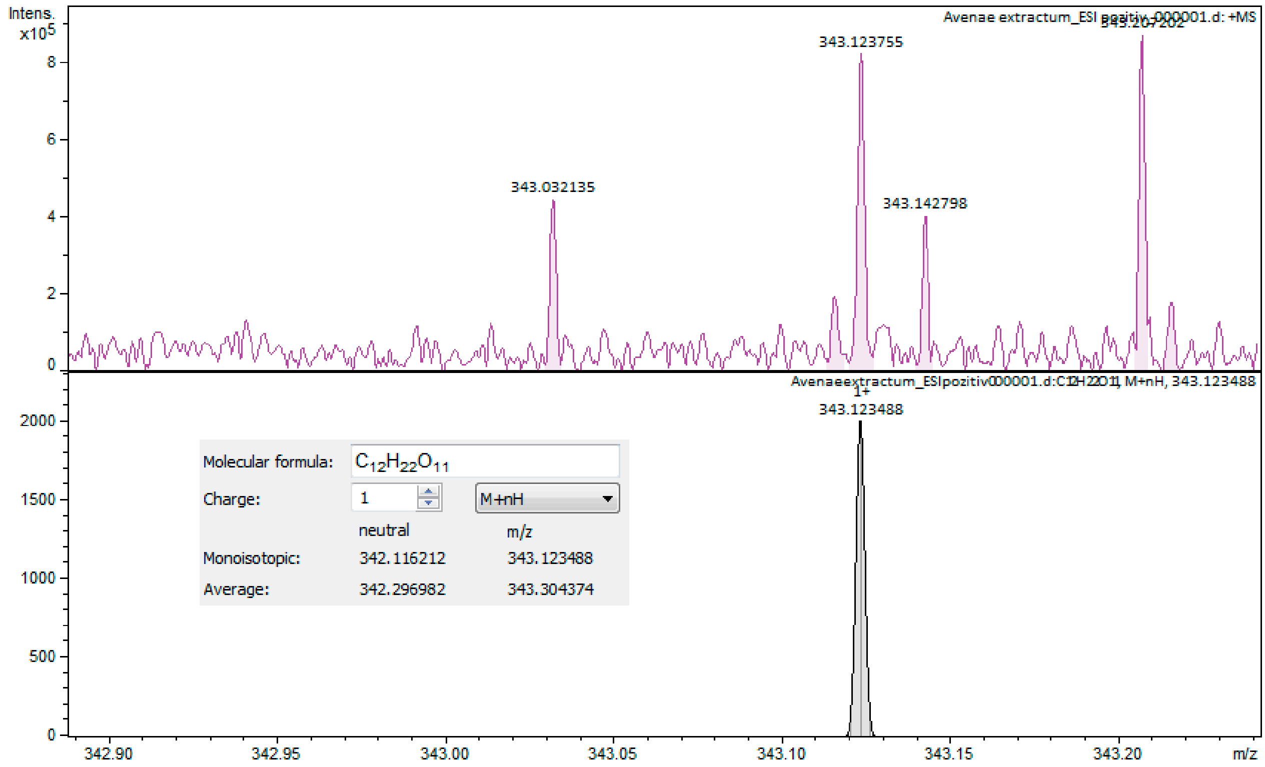

Figure S34. Sucrose ($C_{12}H_{22}O_{11}$) – m/z is 343.12, ESI positive

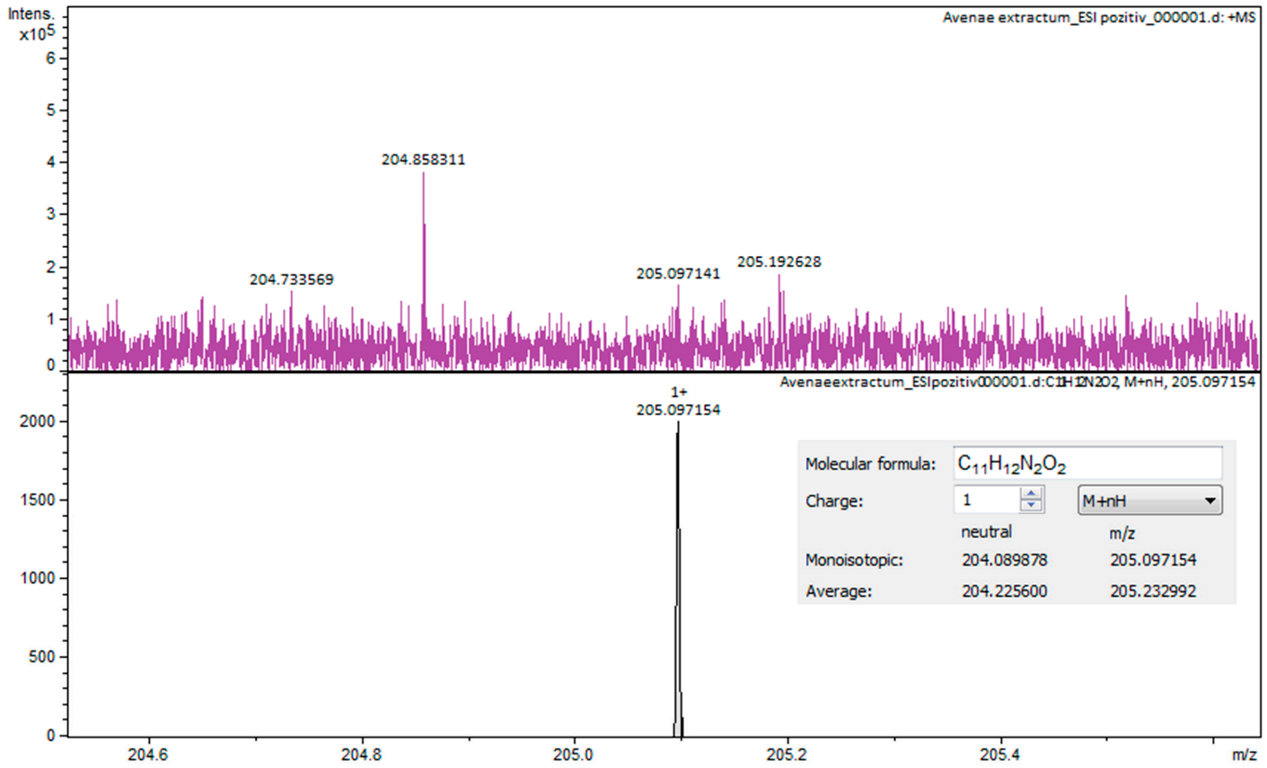

Figure S35. Tryptophan ($C_{11}H_{12}N_2O_2$) – m/z is 205.09, ESI positive

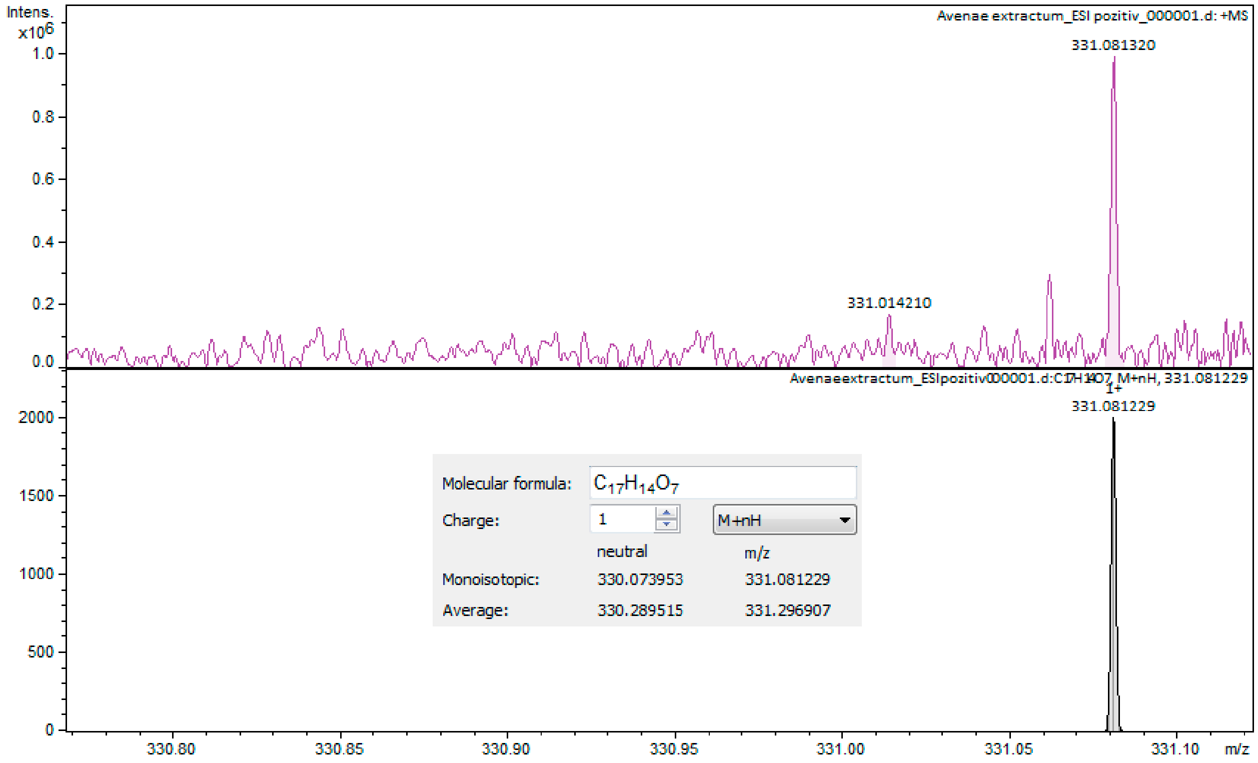

Figure S36. Tricin ($C_{17}H_{14}O_7$) – m/z is 331.08, ESI positive

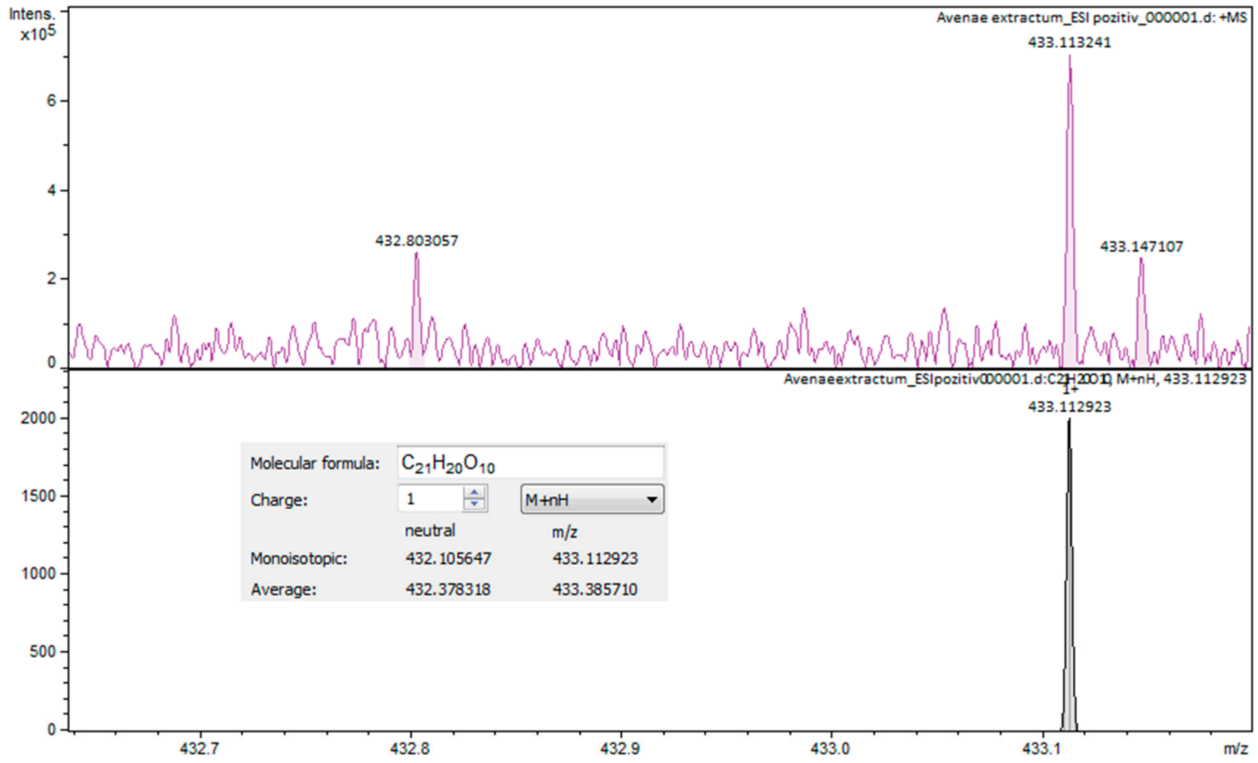

Figure S37. Vitexin ($C_{21}H_{20}O_{10}$) – m/z is 433.11, ESI positive

6. *Avenae extractum* (AE), ESI negative

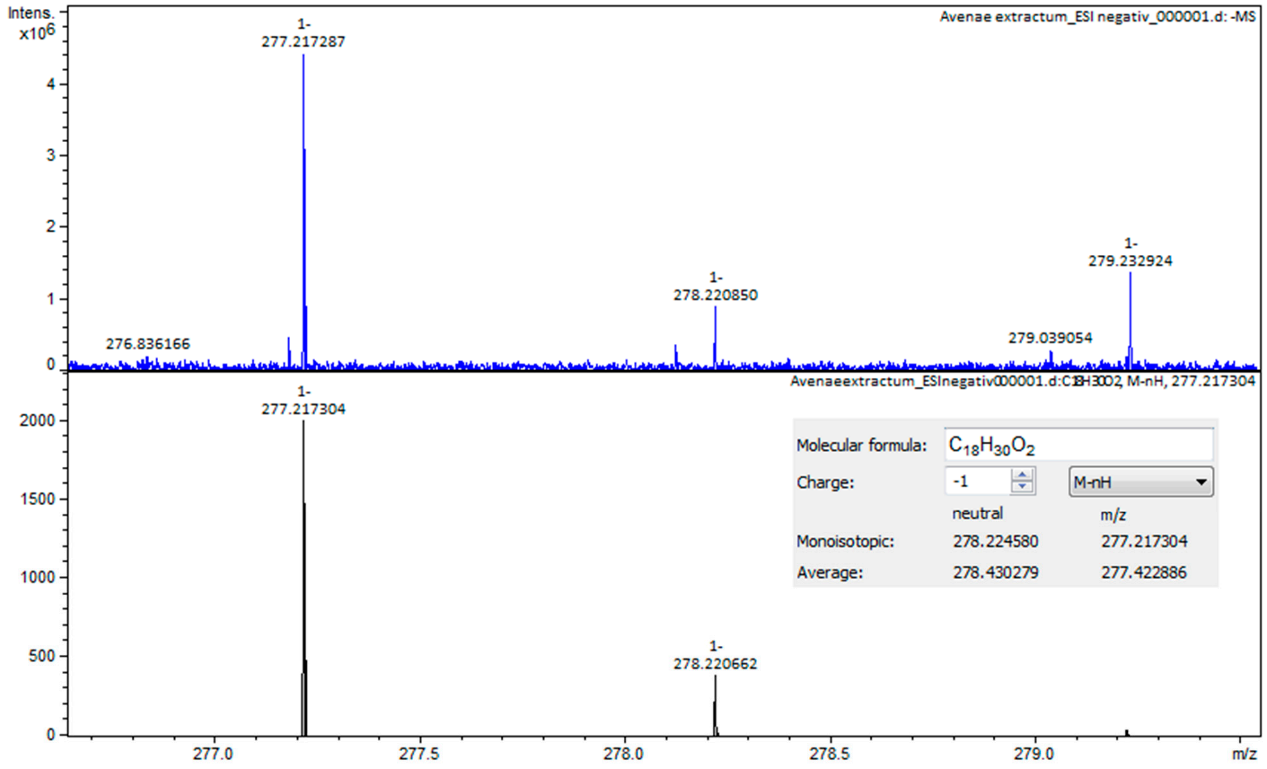

Figure S38. Linolenic acid ($C_{18}H_{30}O_2$) – m/z is 277.21, ESI negative

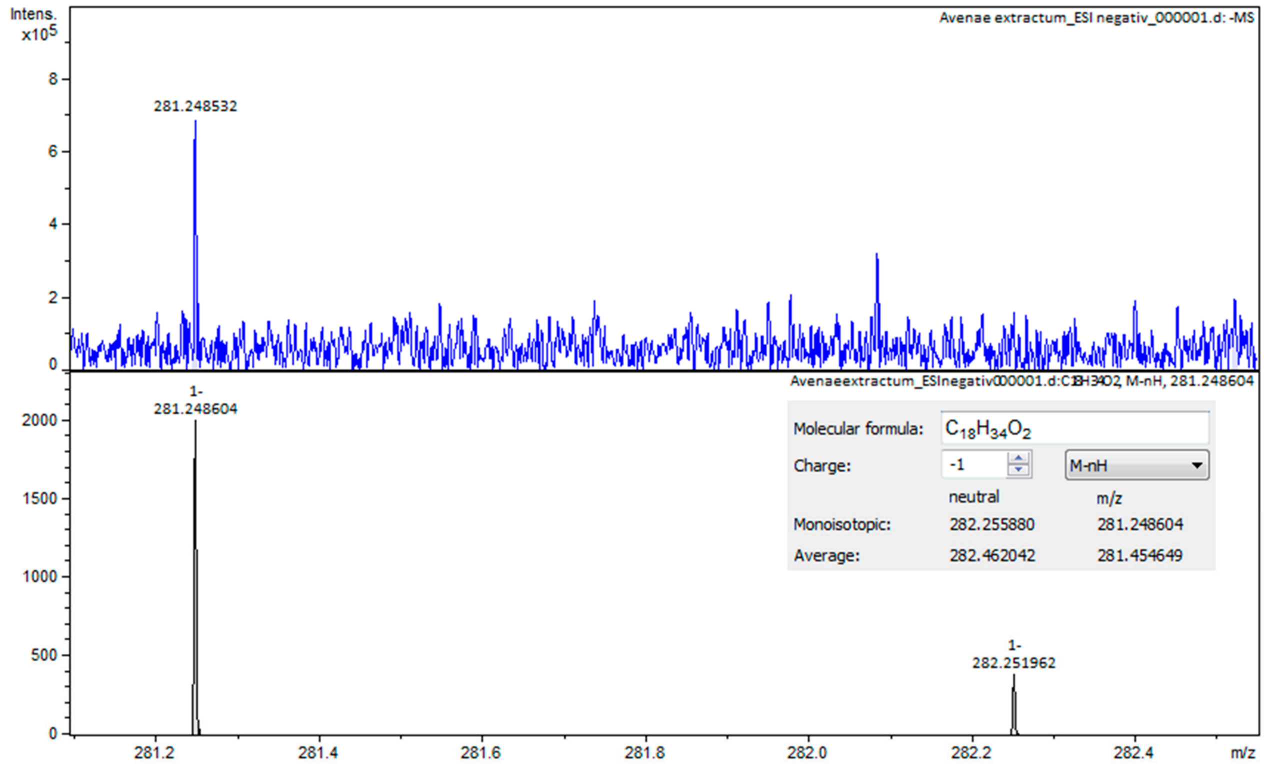

Figure S39. Oleic acid ($C_{18}H_{34}O_2$) – m/z is 281.24, ESI negative

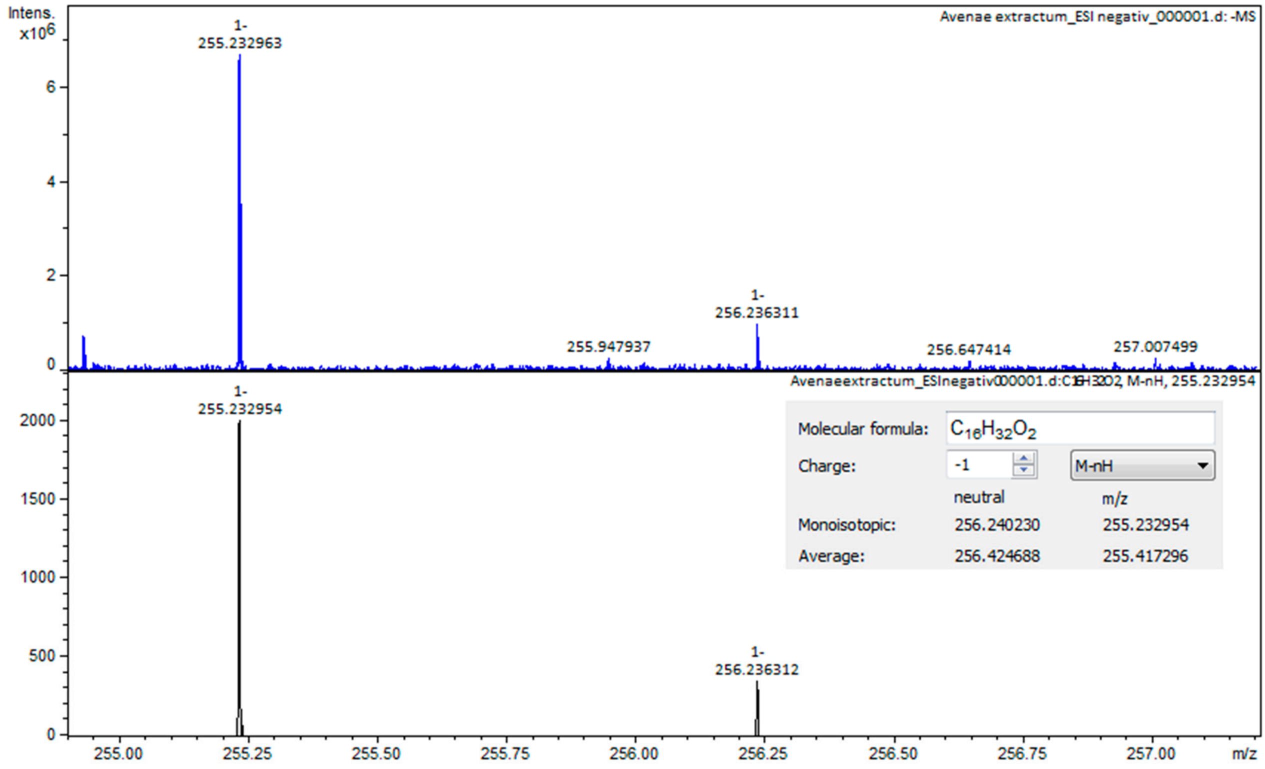

Figure S40. Palmitic acid (C16H32O2) – m/z is 255.23, ESI negative

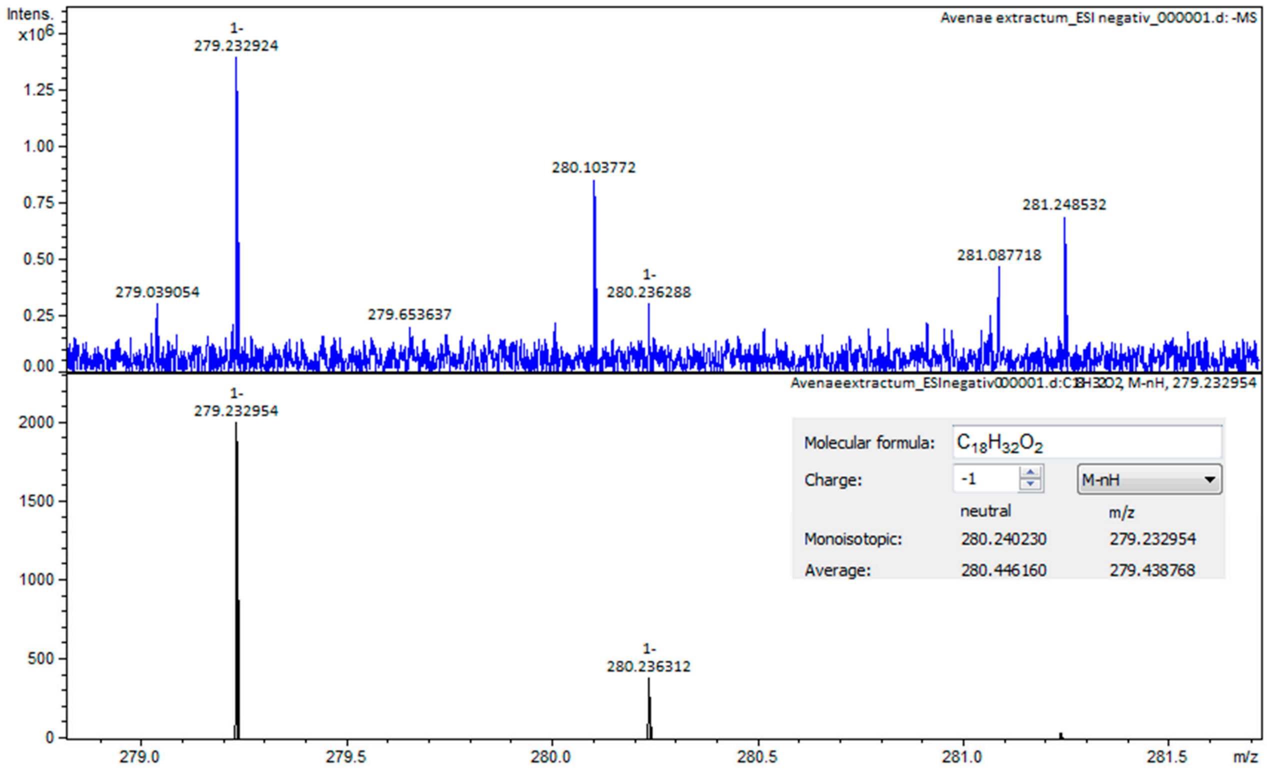

Figure S41. Linoleic acid ($C_{18}H_{32}O_2$) – m/z is 279.23, ESI negative

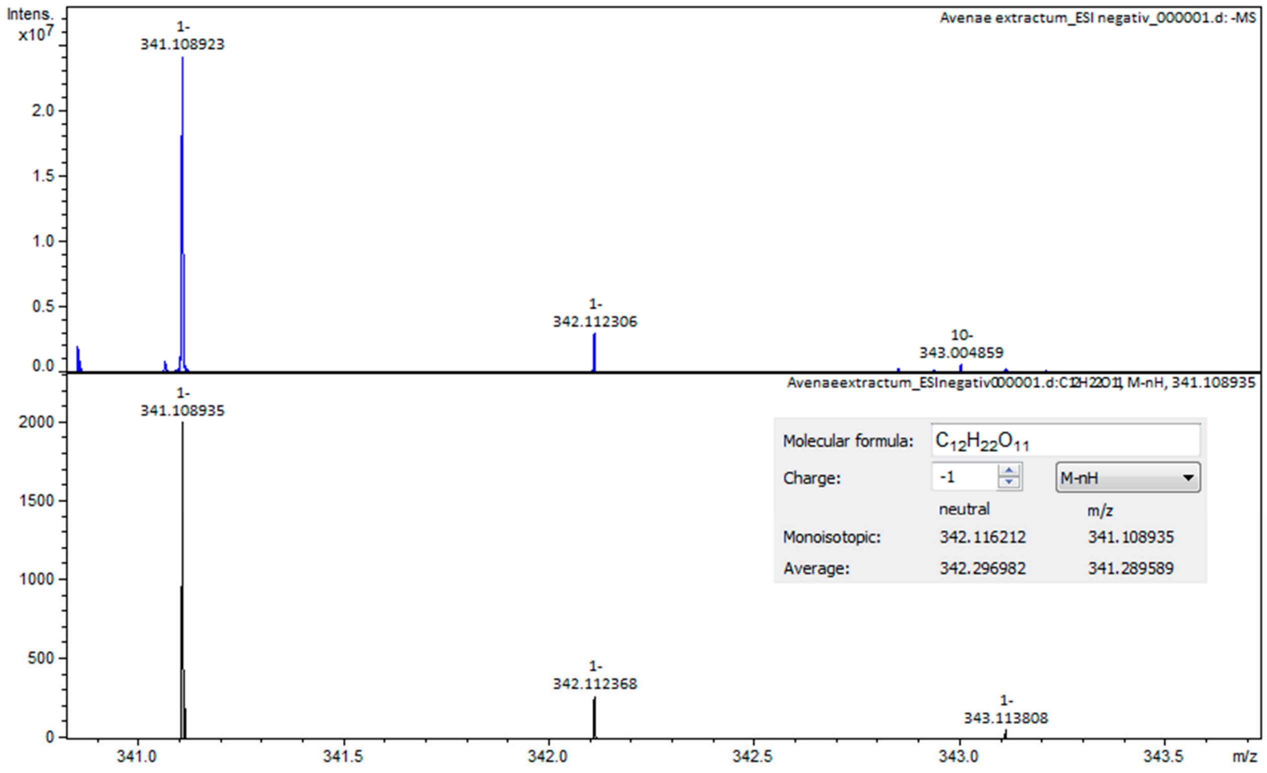

Figure S42. Sucrose ($C_{12}H_{22}O_{11}$) – m/z is 341.10, ESI negative

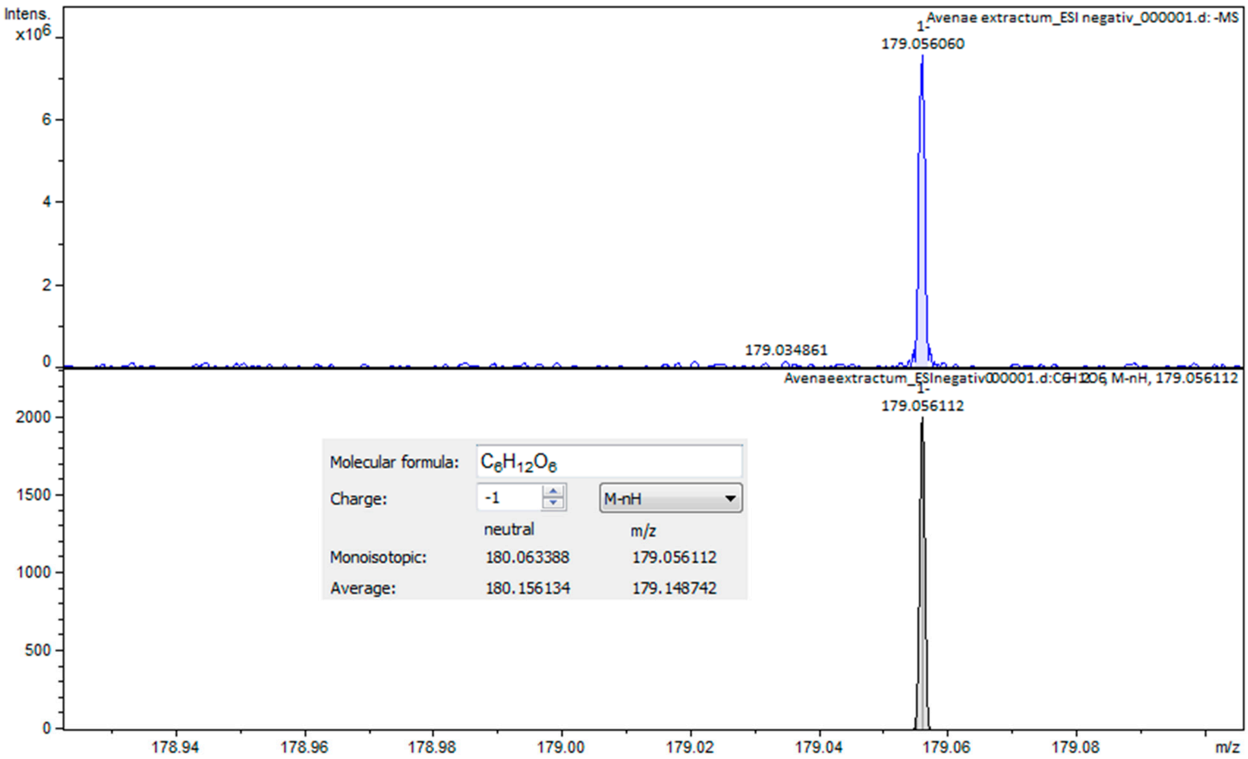

Figure S43. Myo-Inositol/ D-mannose/ D-glucopyranose ($C_6H_{12}O_6$) – m/z is 179.05, ESI negative

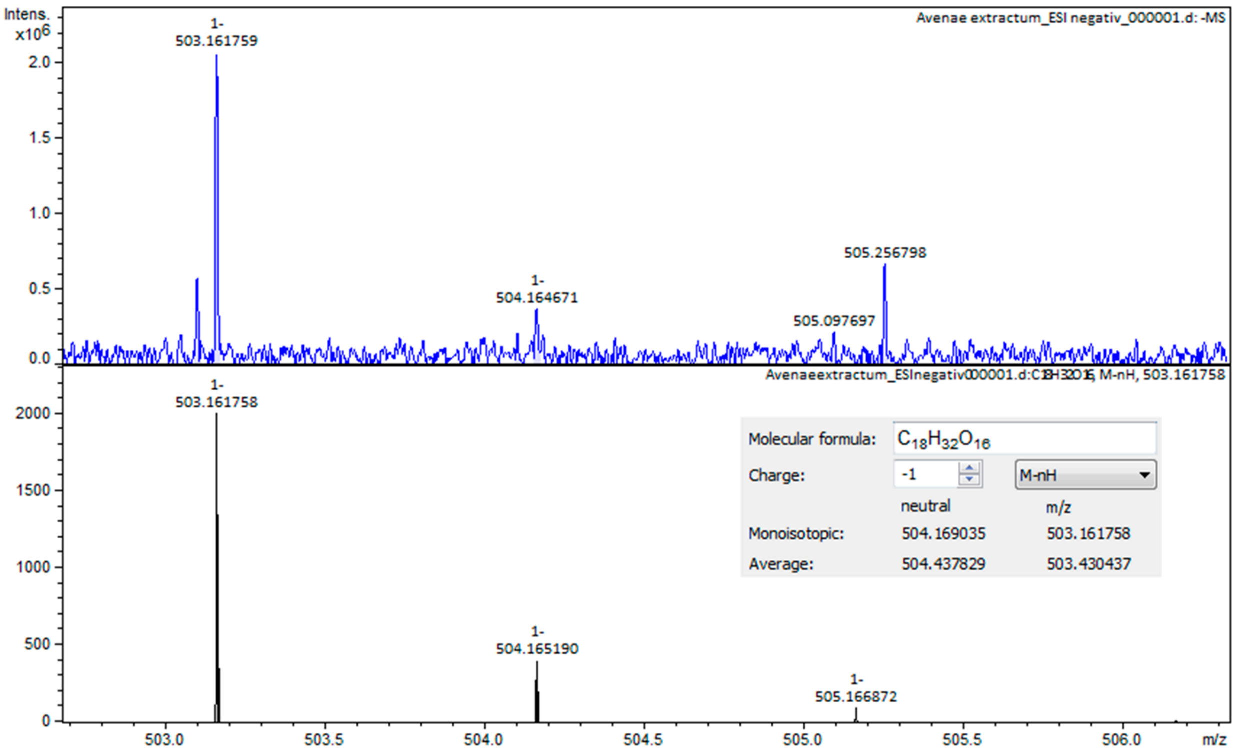

Figure S44. Beta-glucan/ Kestose/ Neokestose ($C_{18}H_{32}O_{16}$) – m/z is 503.16, ESI negative

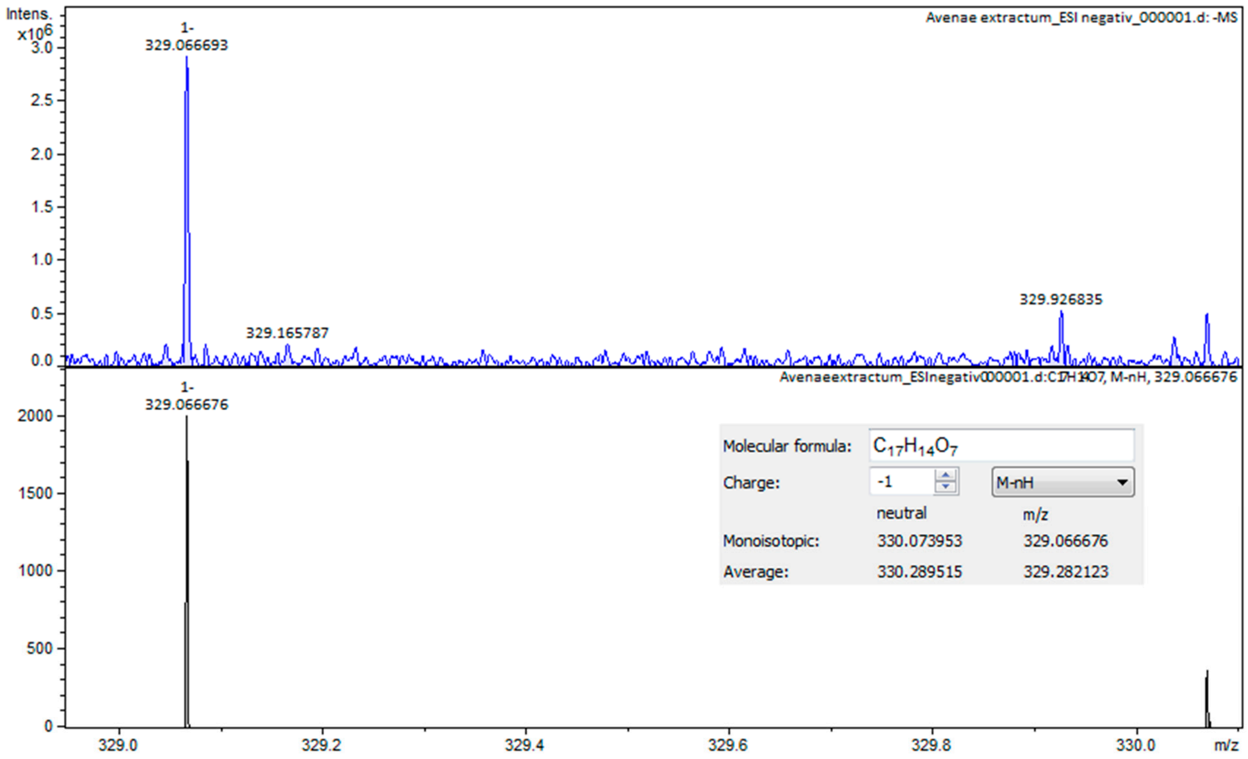

Figure S45. Tricin ($C_{17}H_{14}O_7$) – m/z is 329.06, ESI negative

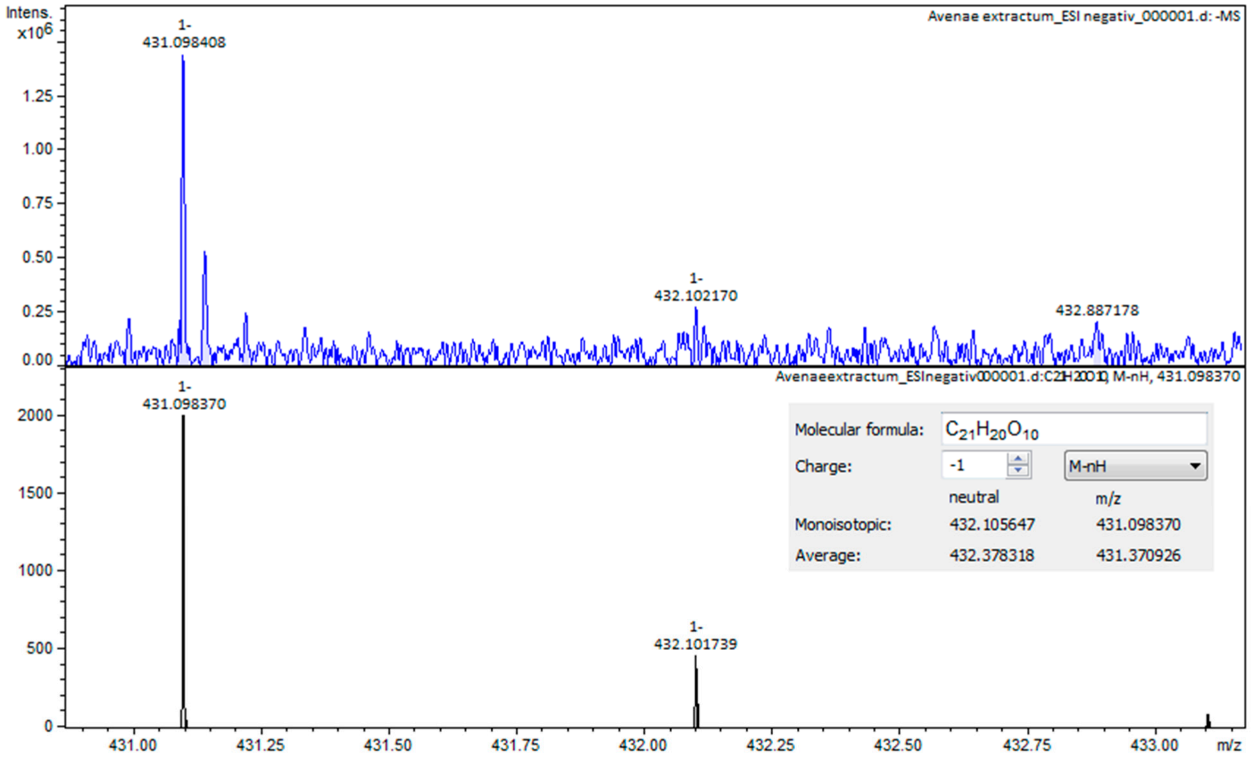

Figure S46. Vitexin $C_{21}H_{20}O_{10}$) – m/z is 431.09, ESI negative

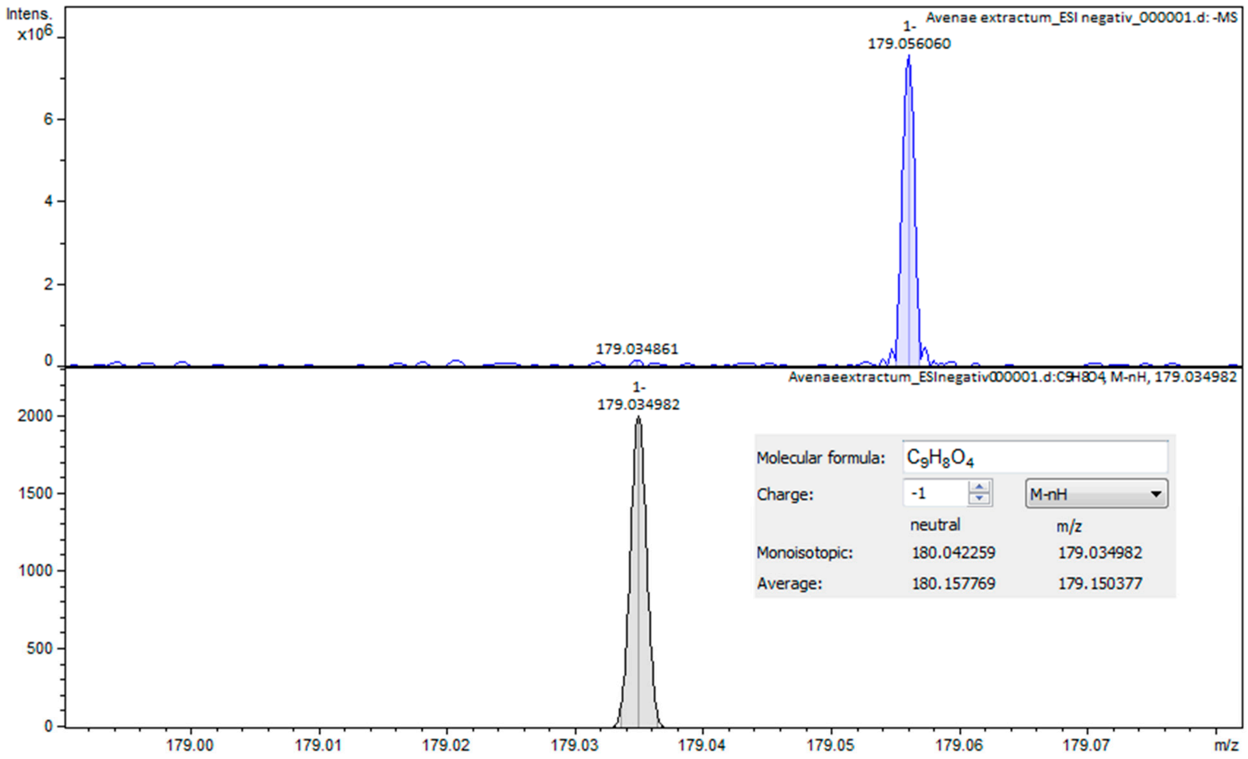

Figure S47. Caffeic acid ($C_9H_8O_4$) – m/z is 179.03, ESI negative

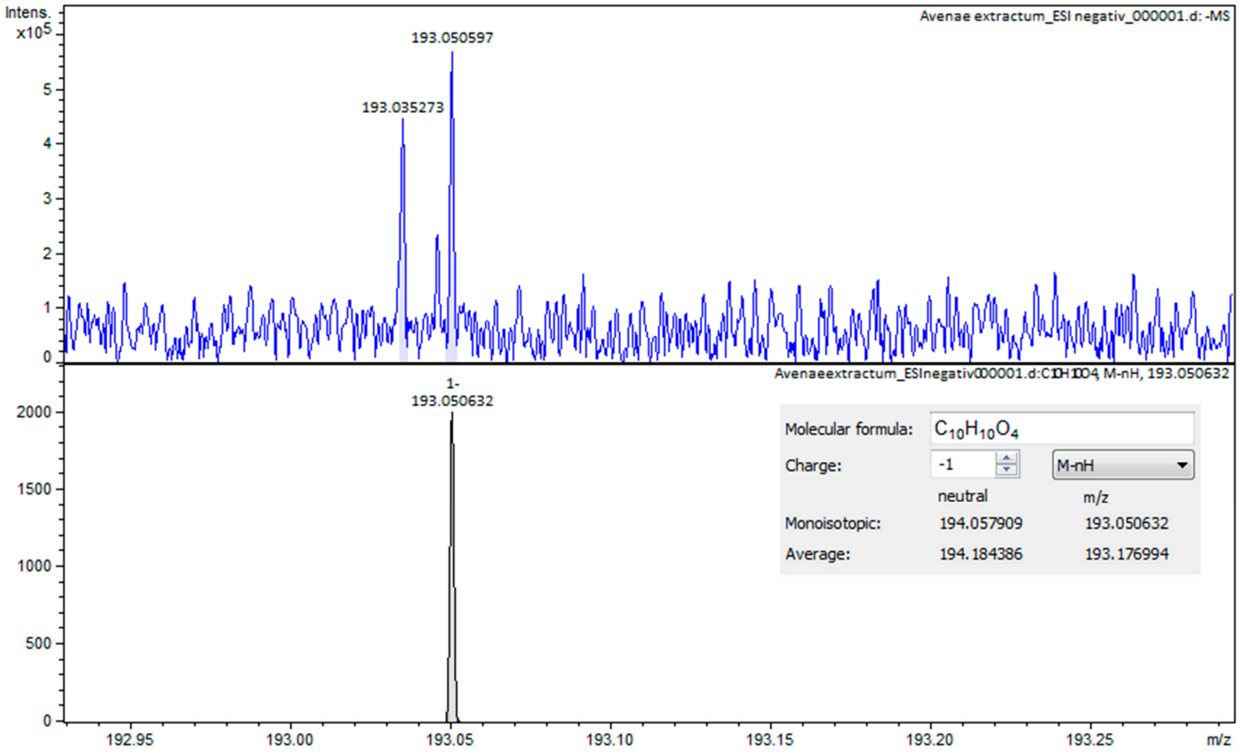

Figure S48. Ferulic acid ($C_{10}H_{10}O_4$) – m/z is 193.05, ESI negative

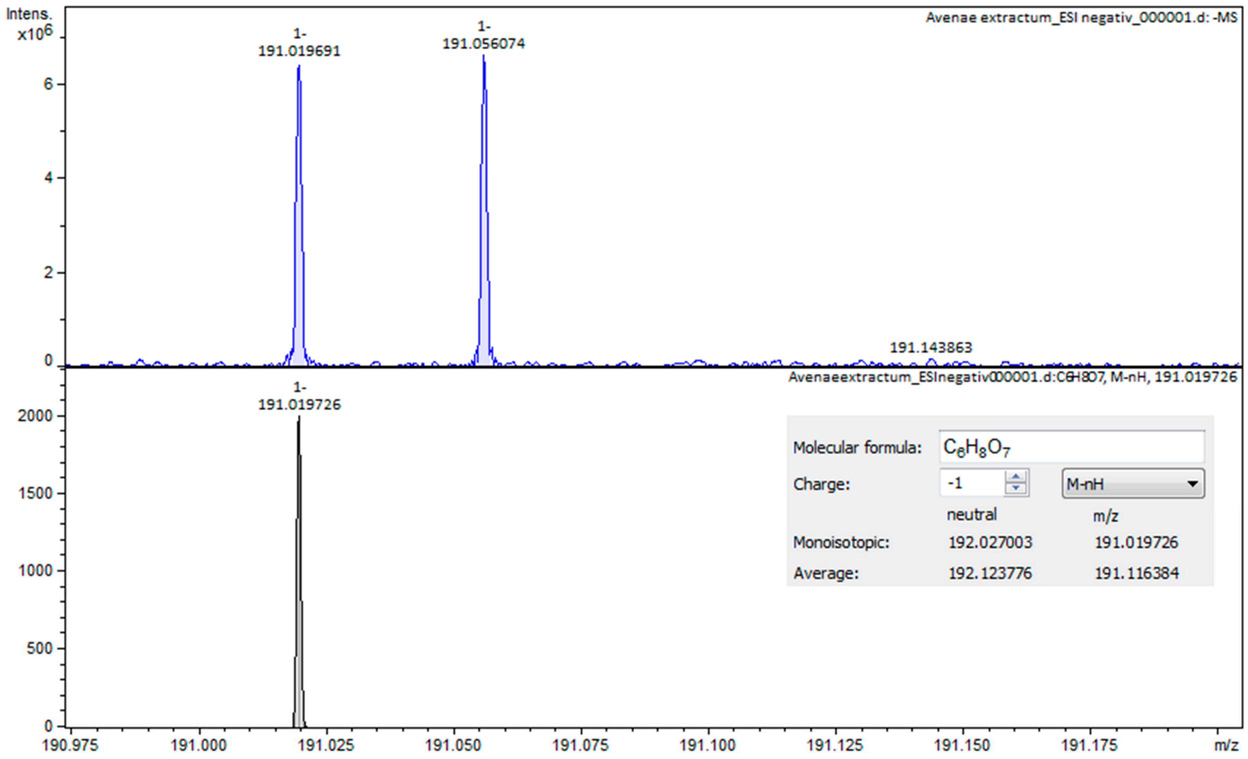

Figure S49. Citric acid ($C_6H_8O_7$) – m/z is 191.01, ESI negative

Table S1. Calculated mass and measured mass for some compounds from *Betulae extractum*, *Liquiritiae extractum* and *Avenae extractum* identified by FT-ICR analysis

Sample name	Compound	Molecular formula	Calculated mass (m/z)		Measured mass (m/z)	
			ESI +	ESI -	ESI +	ESI -
Betulae extractum	Betulinic acid	$C_{30}H_{48}O_3$	457.367622	455.353069	457.367030	455.352750
	Betulin	$C_{30}H_{50}O_2$	443.388357	-	443.387711	-
	Betulinaldehyde	$C_{30}H_{48}O_2$	441.372707	-	441.372232	-
	Betulonic acid	$C_{30}H_{46}O_3$	455.351972	453.337419	455.351537	453.337161
	Betuloside	$C_{16}H_{24}O_7$	329.159480	327.144927	329.159087	327.144745
	Caffeic acid	$C_9H_8O_4$	-	179.034982	-	179.034734
	Erythrodiol	$C_{30}H_{50}O_2$	443.388357	-	443.387711	-
	Kaempferol	$C_{15}H_{10}O_6$	-	285.040462	-	285.040296
	Linoleic acid	$C_{18}H_{32}O_2$	-	279.232954	-	279.232801
	Lupeol	$C_{30}H_{50}O$	427.393443	-	427.393047	-
	Lupenone	$C_{30}H_{48}O$	425.377793	-	425.377374	-
	Myo-inositol	$C_6H_{12}O_6$	-	179.056112	-	179.055943
	Oleanolic acid	$C_{30}H_{48}O_3$	457.367622	455.353069	457.367030	455.352750
	Oleic acid	$C_{18}H_{34}O_2$	-	281.248604	-	281.248488
	Stearic acid	$C_{18}H_{36}O_2$	285.278807	283.264254	285.278431	283.264114
	Ursolic acid	$C_{30}H_{48}O_3$	457.367622	455.353069	457.367030	455.352750
Liquiritiae extractum	Glycyrrhizin / Glycyrrhizinic acid	$C_{42}H_{62}O_{16}$	823.411062	821.396509	823.409702	821.396733
	Glycyrrhetinic acid	$C_{30}H_{46}O_4$	471.346886	469.332334	471.346193	469.332206
	Glabridin	$C_{20}H_{20}O_4$	325.143436	323.128883	325.142893	323.128815
	Linoleic acid	$C_{18}H_{32}O_2$	-	279.232954	-	279.232909

	Liquiritigenin / Isoliquiritigenin	$C_{15}H_{12}O_4$	257.080835	255.066282	257.080415	255.066235
	Myo-inositol	$C_6H_{12}O_6$	-	179.056112	-	179.056068
	Palmitic acid	$C_{16}H_{32}O_2$	257.247507	255.232954	257.247099	255.232910
Avenae extractum	Avenacoside A	$C_{51}H_{82}O_{23}$	1063.531965	-	1063.531160	-
	Beta-glucan	$C_{18}H_{32}O_{16}$	-	503.161758	-	503.161759
	Caffeic acid	$C_9H_8O_4$	-	179.034982	-	179.034861
	Citric acid	$C_6H_8O_7$	-	191.019726	-	191.019691
	D-glucopyranose	$C_6H_{12}O_6$	-	179.056112	-	179.056060
	D-mannose	$C_6H_{12}O_6$	-	179.056112	-	179.056060
	Ferulic acid	$C_{10}H_{10}O_4$	-	193.050632	-	193.050597
	Kestose/ Neokestose	$C_{18}H_{32}O_{16}$	-	503.161758	-	503.161759
	Linoleic acid	$C_{18}H_{32}O_2$	-	279.232954	-	279.232924
	Linolenic acid	$C_{18}H_{30}O_2$	279.231857	277.217304	279.231974	277.217287
	Myo-Inositol	$C_6H_{12}O_6$	-	179.056112	-	179.056060
	Oleic acid	$C_{18}H_{34}O_2$	283.263157	281.248604	283.263248	281.248532
	Palmitic acid	$C_{16}H_{32}O_2$	257.247507	255.232954	257.247315	255.232963
	Sucrose	$C_{12}H_{22}O_{11}$	343.123488	341.108935	343.123755	341.108923
	Tricin	$C_{17}H_{14}O_7$	331.081229	329.066676	331.081320	329.066693
	Tryptophan	$C_{11}H_{12}N_2O_2$	205.097154	-	205.097141	-
	Vitexin	$C_{21}H_{20}O_{10}$	433.112923	431.098370	433.113241	431.098408

S1.2. Standard curve of rutoside for total flavonoid content assay (TFC)

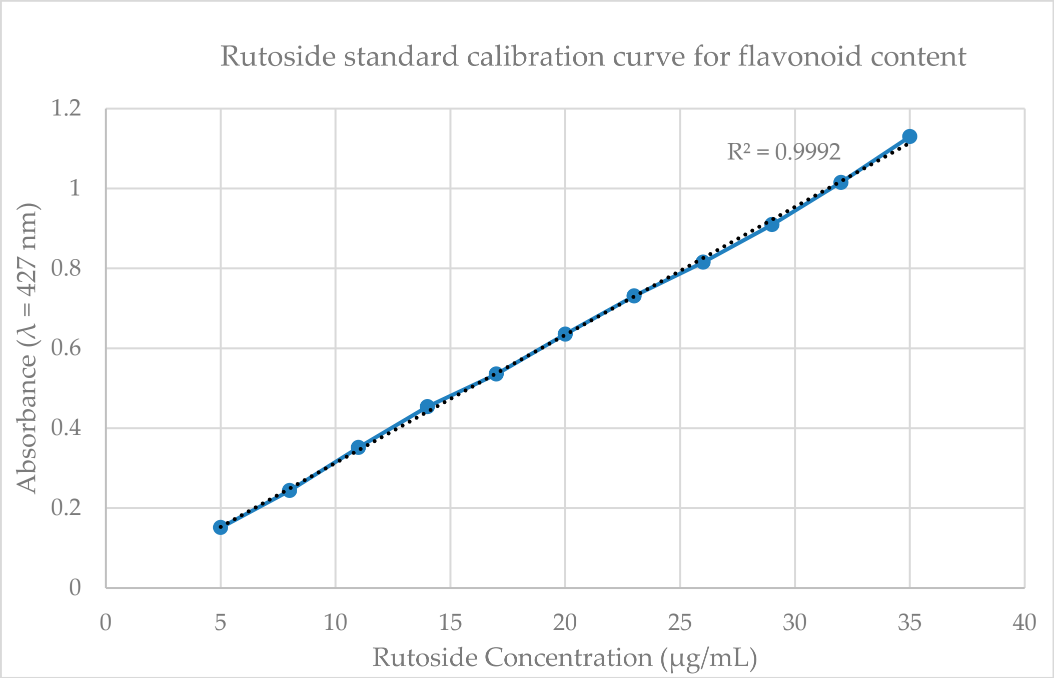

Figure S50. Rutoside standard calibration curve

S1.3. Standard curve of tannic acid for total polyphenols content assay (TPC)

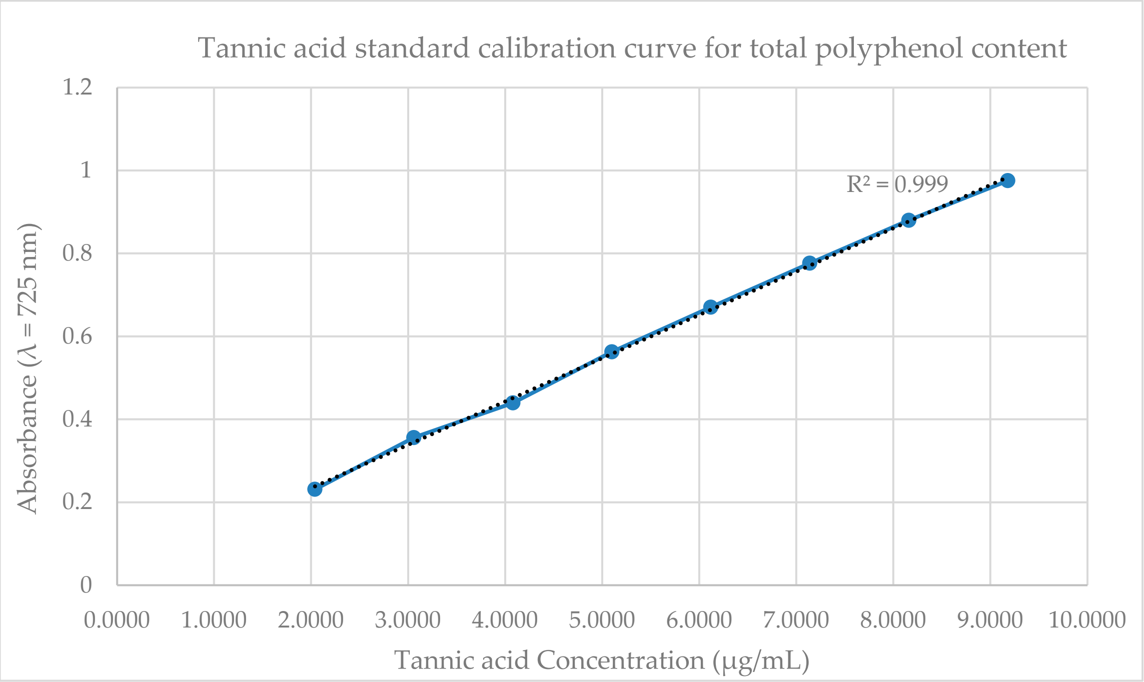

Figure S51. Tannic acid standard calibration curve

S1.4. Standard curve of chlorogenic acid for total phenolic acid content assay (TPA)

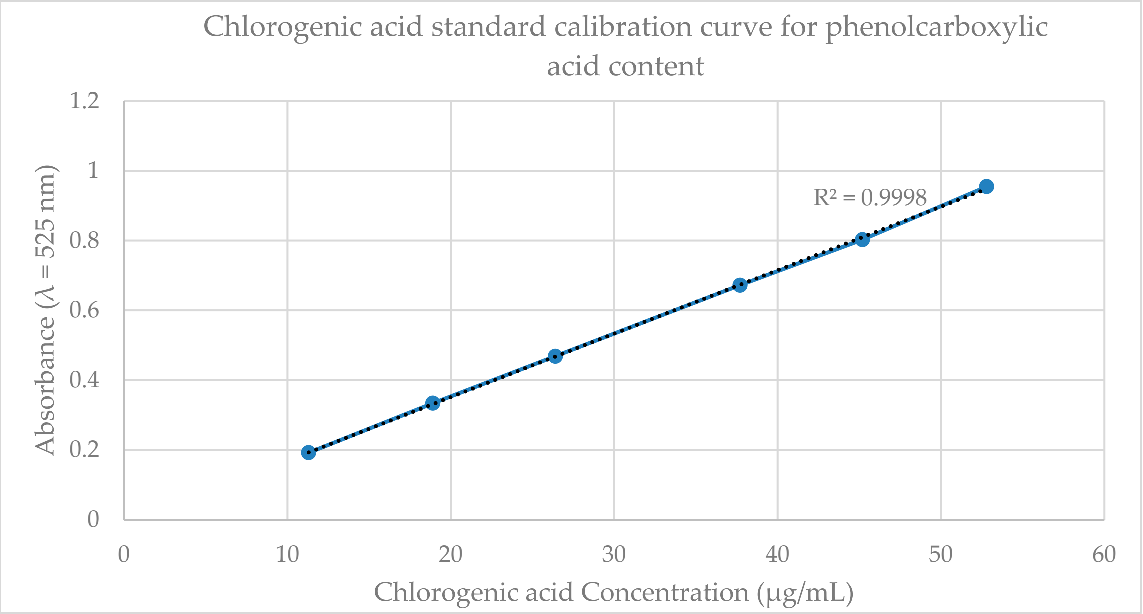

Figure S52. Chlorogenic acid standard calibration curve

S1.5. Standard curve of ascorbic acid for DPPH antioxidant assay

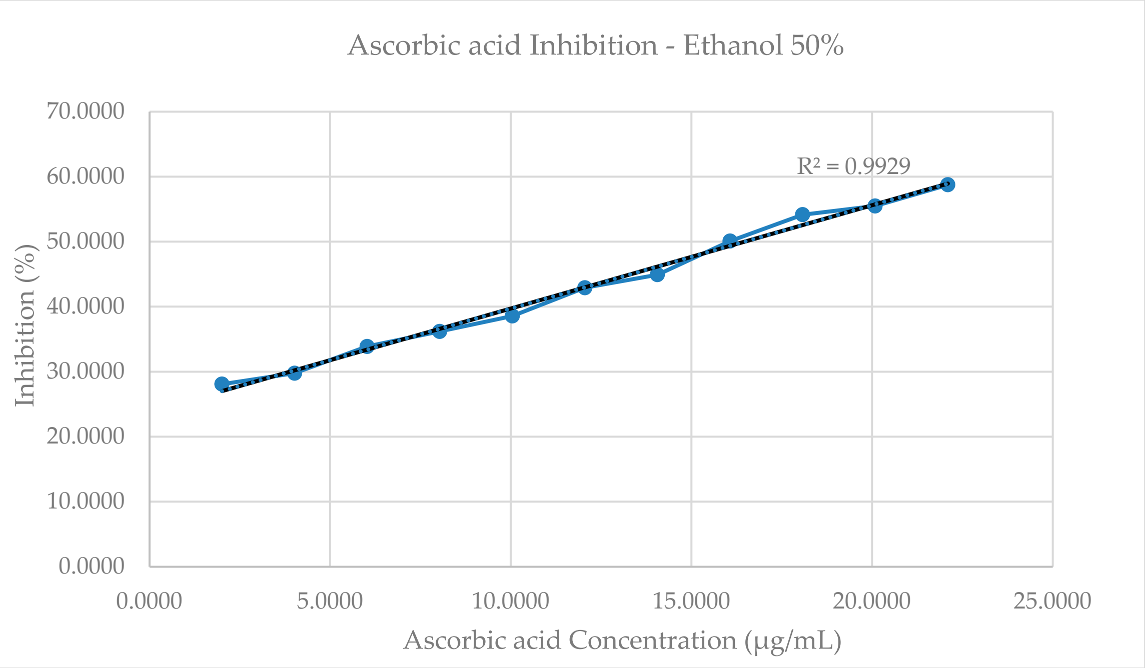

Figure S53. Ascorbic acid standard curve

S1.5. Compounds identified with GC-MS analysis, using derivatization method

Table S2. The compounds identified with the GC-MS analysis in the *Betulae extractum* (BE), grouped in classes

Classes	Presumably identified compounds	% from the total identified compounds
Hexoses	D-(−)-Fructofuranose (isomer 1)<br>D-(−)-Fructofuranose (isomer 2)<br>Glyceric acid<br>D-Mannose<br>D-Glucopyranose<br>Talose<br>D-Altrose<br>D-Allofuranose<br>D-(−)-Fructopyranose<br>Galactopyranose	21.74
Pentoses	Arabinose	0.25
Polyols	D-Mannitol<br>D-Glucitol<br>Erythritol<br>D-Sorbitol<br>Ribitol<br>Adonitol<br>L-(−)-Arabitol<br>Xylitol<br>Glycerol<br>Myo-Inositol	9.45

Disaccharides	D-Pinitol<br>Sucrose<br>D-(+)-Turanose<br>Beta-Lactose<br>2- α -Mannobiose (isomer 1)<br>2- α -Mannobiose (isomer 2)<br>D-(+) Trehalose	8.26
Carboxylic acids	Glycolic acid<br>Succinic acid<br>Erythronic acid<br>Malic acid<br>L-(+)-Tartaric acid<br>D-Gluconic acid<br>3-Hydroxybenzoic acid<br>Quininic acid<br>Phosphoric acid	1.85
Phenolic compounds	Caffeic acid<br>4-Hydroxyphenylethanol (Tyrosol)<br>Catechollactate<br>Protocatechoic acid<br>4-Coumaric acid<br>Hydrocinnamic acid<br>Phenolic derivative<br>Catechine (2R-E)-	4.16
Fatty acids	Palmitic acid<br>Linoleic acid<br>Oleic acid<br>2-Palmitoylglycerol<br>1-Monopalmitin<br>Glycerol monostearate<br>Stearic acid	2.31
Triterpenes	Betulin<br>Lupeol<br>Lupeolic derivative 1<br>Lupeolic derivative 2<br>Lupeolic derivative 3<br>Lupeolic derivative 4<br>Betulinaldehide<br>Betulinic derivative<br>Ursolic acid	51.96
Amino acids	L-5-Oxoproline	0.06

Table S3. Compounds identified with GC-MS analysis for *Betulae extractum*

Compound number	Compound name	Retention Time (min)	% Area
		min	
1	Glycolic acid, 2TMS	4,301	0,1813
2	Phosphoric acid, triTMS	7,879	0,1107
3	Glycerol, 3TMS	7,936	1,5537
4	Succinic acid (2TMS)	8,614	0,0655
5	Glyceric acid, 3TMS	8,855	0,0656

6	Malic acid, 3TMS	11,183	0,3696
7	Erythritol, 4TMS	11,448	0,3316
8	L-5-Oxoproline, 2TMS	11,645	0,0671
9	4-Hydroxyphenylethanol, 2TMS (Tyrosol)	12,387	0,1461
10	L-(+)-Tartaric acid, 4TMS	12,521	0,0488
11	Arabinose, 4TMS	13,44	0,2648
12	Ribitol, 5TMS	14,234	0,1361
13	L-(−)-Arabitol, 5TMS	14,365	0,1147
14	Derivat fenolic	14,496	1,9894
15	Xylitol, 5TMS derivative	14,587	3,4932
16	Adonitol, 5TMS	14,667	0,103
17	D-(−)-Fructofuranose, 5TMS (isomer 1)	15,951	0,635
18	D-(−)-Fructofuranose, 5TMS (isomer 2)	16,082	1,8206
19	D-(−)-Fructopyranose, 5TMS	16,159	2,1736
20	Protocatechoic acid, 3TMS	16,22	0,4434
21	D-Pinitol	16,377	0,072
22	D-Allofuranose, 5TMS	16,629	0,2145
23	Quininic acid (5TMS)	16,847	0,653
24	D-Mannose, 5TMS	17,427	4,1658
25	Talose, 5TMS	17,591	0,1953
26	D-Altrose, 5TMS	17,618	0,1166
27	D-Mannitol, 6TMS	18,047	3,396
28	D-Sorbitol, 6TMS	18,168	0,3302
29	D-Glucitol, 6TMS	18,242	0,0826
30	D-Glucopyranose, 5TMS	18,939	12,3032
31	D-Gluconic acid, 6TMS	19,238	0,3691
32	Palmitic Acid, TMS	20,09	0,4072
33	Catechollactate, 3TMS	20,298	0,0501
34	4-Coumaric acid, 2TMS	20,455	0,2001
35	Myo-Inositol, 6TMS	20,791	0,3479
36	Caffeic acid, 3TMS	21,606	0,0685
37	Linoleic acid, TMS	22,853	0,3442
38	Oleic Acid,TMS	22,95	0,147
39	Stearic acid, TMS	23,406	0,0379
40	D-(+)-Turanose, 8TMS	27,793	0,1589
41	2-Palmitoylglycerol, 2TMS	28,299	0,0633
42	2-a-Mannobiose,8TMS (isomer 1)	28,621	1,057
43	1-Monopalmitin, 2TMS	28,802	0,9938
44	2-α-Mannobiose,8TMS (isomer 2)	29,134	0,2089
45	Sucrose, 8TMS	29,49	2,3609
46	2-a-Mannobiose, 8TMS (isomer 2)	29,741	0,5848
47	Galactopyranose, 5TMS	30,204	1,0039
48	D-(+)-Trehalose, 8TMS ether	30,986	0,8595
49	Glycerol monostearate, 2TMS	31,572	0,4154
50	ß-Lactose, 8TMS	32,263	3,4
51	Catechine (2R-E)-, 5TMS derivative	32,927	1,1657
52	Lupeol, TMS	39,812	0,8684
53	3-epi-betulinaldehide, 3TMS	42,508	40,6877
54	Betulin	42,639	3,3224

55	Betulinaldehyde	42,861	3,353
56	Ursolic acid 2TMS	44,618	1,7849

Table S4. The compounds identified with the GC-MS analysis in the *Liquiritiae extractum* (LE), grouped in classes

Classes	Presumably identified compounds	% from the total identified compounds
Hexoses	D-(−)-Fructofuranose, pentakis(trimethylsilyl) ether (isomer 1)<br>D-(−)-Fructofuranose, pentakis(trimethylsilyl) ether (isomer 2)<br>D-Glucopyranose<br>D-Altrose<br>Talose<br>D-Talofuranose (isomer 1)<br>D-Talofuranose (isomer 2)<br>D-(−)-Fructopyranose<br>Methyl a-D-glucofuranoside<br>D-Mannose<br>D-Allofuranose	10.73
Polyols	D-Mannitol<br>D-Sorbitol<br>D-Pinitol<br>Xylitol<br>Meso-Erythritol<br>Glycerol<br>Myo-Inositol<br>D-Pinitol<br>1-Deoxypentitol	8.42
Disaccharides	Sucrose<br>D-(+)-Turanose<br>D-Trehalose<br>Maltose<br>3-a-Mannobiose<br>D-(+)-Cellobiose, (isomer 1)<br>D-(+)-Cellobiose, (isomer 2)	72.4
Carboxylic acids	Glyceric acid<br>Phosphoric acid<br>Benzoic acid<br>Succinic acid<br>Fumaric acid<br>Malic acid<br>Erythronic acid<br>Arabinonic acid<br>D-Gluconic acid<br>Galacturonic acid<br>Keto-I-gluconic acid	1.90
Phenolic compounds	Caffeic acid<br>4-Coumaric acid<br>Hydrocaffeic acid (3,4-dihydroxyhydrocinnamic acid)	2.39

Fatty acids	Hydrocinnamic acid<br>4-Hydroxybenzoic acid<br>Cinnamic acid<br>Palmitic acid<br>Oleic acid<br>Stearic acid<br>Linoleic acid<br>Glycerol monostearate<br>L-Threonic acid	0.61
Amino acids and derivatives	L-Proline<br>5-Oxoproline<br>L-Alanine<br>L-Valine<br>L-Leucine<br>Serine<br>L-Threonine<br>Homoserine<br>Pipecolic acid<br>L-Aspactic acid<br>4-Aminobutanoic acid<br>L-Glutamic acid<br>Phenylalanine	3.23
Alpha hydroxy acids	Hydroxyglutaric acid	0.01
Monoterpene glycosidic	Aucubin	0.15
Hydroxy steroids	Equilin	0.03

Table S5. Compounds identified with GC-MS analysis for *Liquiritiae extractum*

Compound number	Compound name	Retention Time (min)	% Area
1	L-Alanine, 2TMS	4,815	0,0849
2	L-Valine, 2TMS	6,921	0,0336
3	Benzoic Acid, TBDMS	7,538	0,0278
4	Phosphoric acid TMS	7,877	0,0191
5	Glycerol, 3TMS	7,934	0,2585
6	L-Leucine, 2TMS	8,272	0,0161
7	L-Proline, 2TMS	8,353	2,7575
8	Succinic acid (2TMS)	8,618	0,0317
9	Glyceric acid, 3TMS	8,856	0,0397
10	Fumaric acid, di-TMS	9,205	0,1265
11	Serina, 3TMS	9,336	0,0489
12	L-Threonine, 3TMS	9,725	0,0336
13	Homoserine, 3TMS	10,657	0,0068
14	Malic acid, 3TMS	11,183	0,8952
15	meso-Erythritol, 4TMS	11,448	0,0499
16	Pipecolic acid, 2TMS	11,479	0,0993
17	L-Aspartic acid, 3TMS	11,629	0,0434
18	5-Oxoproline, 2TMS	11,639	0,0328
19	4-Aminobutanoic acid, 3TMS	11,784	0,0538

20	1-Deoxypentitol, 4TMS	11,871	0,0473
21	Erythronic acid 4TMS	11,935	0,0329
22	L-Threonic acid, 3TMS	12,173	0,0185
23	Hydroxyglutaric acid, 3TMS	12,407	0,0136
24	L-Valine, 2TMS	12,515	0,0746
25	L-Glutamic acid, 3TMS	13,068	0,0303
26	Phenylalanine, 2TMS	13,185	0,0195
27	4-Hydroxybenzoic acid, 2TMS	13,216	0,0254
28	Arabinonic acid, 3TMS	13,266	0,1666
29	Xylitol, 5TMS	14,587	0,0486
30	Hydrocinnamic acid, 2TMS	15,281	0,6234
31	2-Keto-l-gluconic acid,	15,55	0,0539
32	Methyl a-D-glucofuranoside, 4TMS	15,885	0,0234
33	D-(−)-Fructofuranose, pentakis(trimethylsilyl) ether (isomer 1)	15,952	0,7894
34	D-(−)-Fructofuranose, pentakis(trimethylsilyl) ether (isomer 2)	16,086	2,4753
35	D-(−)-Fructopyranose, 5TMS	16,157	0,6317
36	D-Pinitol 5TMS	16,381	7,3086
37	D-Allofuranose, 5TMS	16,626	0,4299
38	D-Mannose, 5TMS	17,428	1,7647
39	Talose, 5TMS	17,595	0,1183
40	D-Altrose, 5TMS	17,626	0,0984
41	D-Mannitol, 6TMS	18,048	0,0873
42	D-Sorbitol, 6TMS	18,172	0,1394
43	Hydrocaffeic acid (3TMS)	18,306	0,04
44	D-Pinitol, 5TMS ether	18,467	0,1767
45	D-Talofuranose, 5TMS (isomer 1)	18,672	0,1383
46	D-Glucopyranose, 5TMS	18,94	4,1319
47	D-Talofuranose, 5TMS (isomer 2)	19,041	0,1342
48	D-Gluconic acid, 6TMS	19,232	0,1418
49	Cinnamic acid 3TMS	20,057	0,134
50	Palmitic Acid, TMS	20,08	0,0645
51	4-Coumaric acid, 2TMS	20,456	1,5583
52	Myo-Inositol, 6TMS	20,791	0,2913
53	Caffeic acid, 3TMS	21,61	0,0099
54	Linoleic acid, TMS	22,85	0,1677
55	Oleic Acid, TMS	22,951	0,0317
56	Stearic acid, TMS	23,414	0,0393
57	D-(+)-Cellobiose, (isomer 1), 8TMS	24,319	0,018
58	Galacturonic acid, 5TMS	25,527	0,3575
59	D-(+)-Cellobiose, (isomer 2), 8TMS	26,492	0,0247
60	D-(+)-Turanose, 8TMS ether	27,797	6,2815
61	D-Trehalose, 7TMS	28,79	3,7401
62	Sucrose, 8TMS	29,548	61,7973
63	Maltose, 8TMS	30,691	0,1739
64	Glycerol monostearate, 2TMS	31,573	0,2933
65	Equilin, TMS	32,163	0,0325
66	3-a-Mannobiose, 8TMS isomer 1	39,655	0,3895
67	Aucubin, 6TMS ether	41,775	0,1523

Table S6. Compounds identified with the GC-MS analysis for *Avenae extractum* (AE), grouped in classes

Classes	Presumably identified compounds	% from the total identified compounds
	D-(−)-Fructofuranose, pentakis(trimethylsilyl) ether (isomer 1)	
	D-(−)-Fructofuranose, pentakis(trimethylsilyl) ether (isomer 2)	
	D-Glucopyranose	
Hexoses	D-Allofuranose	39.43
	Alfa-D-Mannopyranose	
	D-(+)-Talofuranose	
	D-Psicopyranose	
	D-Fructose	
	D-Glucose	
	D-Mannitol	
	Beta-D-Lactose	
Polyols	Glycerol	2.20
	Xylitol	
	Erythritol	
	Myo-Inositol	
	Sucrose	
Disaccharides	D-(+)-Turanose	29.84
	D-Trehalose	
Sugar derivatives	Glyceryl-glycoside	0.55
	Glyceric acid	
	Phosphoric acid	
	Lactic acid	
	Succinic acid	
	Fumaric acid	
	Malic acid	
Carboxylic acids	Quininic acid	21.99
	Citric acid	
	Erythronic acid	
	D-Gluconic acid	
	D-(+)-Galacturonic acid	
	Itaconic acid	
	Ribonic acid	
	2-Keto-L-gluconic acid	
Phenolic compounds	Caffeic acid	0.22
	4-Coumaric acid	
	Palmitic acid	
	α-Linolenic acid	
	Stearic acid	
	Linoleic acid	
Fatty acids	1-Monopalmitin	3.57
	Glycerol monostearate	
	L-Threonic acid, tris(trimethylsilyl) ether	
	Di-hidroxibutanoic acid	
	5-Oxoproline	1.78

Amino acids and derivatives	L-Alanine<br>L-Valine<br>L-Isoleucine<br>L-Serine<br>L-Threonine<br>L-Aspactic acid<br>4-Aminobutanoic acid<br>L-Glutamic acid<br>Phenylalanine	
Alpha hydroxy acids	Glycolic acid	0.14
Diamide	Urea	0.06
Other	2-Isopropyl-3-ketobutyrate	0.22

Table S7. Compounds identified with GC-MS analysis for *Avenae extractum*

Compound number	Compound name	Retention Time (min)	% Area
1	Lactic Acid, 2TMS	4.022	1.3501
2	Glycolic acid, 2TMS	4.318	0.1414
3	L-Alanine, 2TMS	4.834	0.4262
4	L-Valine, 2TMS	6.94	0.1441
5	Urea, 2TMS	7.366	0.0573
6	Phosphoric acid, TMS	7.916	3.6387
7	Glycerol, 3TMS	7.953	1.1858
8	L-Isoleucine, 2TMS	8.288	0.0989
9	Succinic acid (2TMS)	8.634	0.8848
10	Glyceric acid, 3TMS	8.868	0.3679
11	Itaconic acid, 2TMS	9.07	0.0382
12	Fumaric acid (2TMS)	9.22	1.1657
13	L-Serine, 3TMS	9.348	0.1012
14	L-Threonine, 3TMS	9.734	0.1316
15	Di-hidroxibutanoic acid, 3TMS	10.418	0.0493
16	2-Isopropyl-3-ketobutyrate, 2TMS	10.616	0.2191
17	L-Aspartic acid, 3TMS	10.944	0.1608
18	Malic acid, 3TMS	11.189	9.6273
19	Erythritol, 4TMS	11.451	0.0502
20	5-Oxoproline, 2TMS	11.662	0.5914
21	4-Aminobutanoic acid, 3TMS	11.799	0.0472
22	Erythronic acid, 4TMS	11.95	0.1361
23	L-Threonic acid, tris(trimethylsilyl) ether-3TMS	12.202	1.1761
24	L-Glutamic acid, 3TMS	13.174	0.0396
25	Phenylalanine, 2TMS	13.295	0.0449
26	Xylitol, 5TMS	14.925	0.0645
27	2-Keto-L-gluconic acid, 5TMS	15.767	0.3745
28	Ribonic acid, 5TMS	15.988	0.319
29	D-(−)-Fructofuranose, pentakis(trimethylsilyl) ether (isomer 1)	16.635	5.3487
30	D-(−)-Fructofuranose, pentakis(trimethylsilyl) ether (isomer 2)	16.806	11.0069

31	D-Psicopyranose, 5TMS	16.887	1.774
32	Citric acid, 4TMS	16.954	0.5083
33	D-(+)-Talofuranose, 5TMS	17.537	1.4911
34	D-Fructose, 5TMS	17.625	0.2509
35	Quininic acid (5TMS)	17.812	2.7279
36	a-D-Mannopyranose, 5TMS	18.53	6.0917
37	D-Glucose, 5TMS	18.745	0.4868
38	D-Mannitol, 6TMS	19.204	0.0943
39	4-Coumaric acid, 2TMS	19.399	0.1499
40	D-Allofuranose, 5TMS	19.824	0.3743
41	D-Glucopyranose, 5TMS	20.103	12.6212
42	D-Gluconic acid, 6TMS	20.341	0.7293
43	Palmitic Acid, TMS	21.072	0.2526
44	Myo-Inositol, 6TMS	21.612	0.245
45	Caffeic acid, 3TMS	22.222	0.067
46	Linoleic acid, TMS	23.074	0.252
47	a-Linolenic acid, TMS	23.141	1.1107
48	Stearic acid, TMS	23.43	0.036
49	Glyceryl-glycoside, TMS	24.1	0.5521
50	D-(+)-Galacturonic acid, 5TMS	24.674	0.1113
51	Sucrose, 8TMS	25.945	1.2535
52	D-(+)-Turanose, 8TMS	26.488	1.5896
53	1-Monopalmitin, 2TMS	26.532	0.5171
54	Sucrose, 8TMS	26.89	26.7859
55	Glycerol monostearate, 2TMS	28.208	0.1653
56	ß-D-Lactose, 8TMS	32.95	0.5667
57	D-(+)-Trehalose, 8 TMS	35.784	0.2079

S1.6. Statistical analysis

Table 8. Multiple comparisons between groups for ABTS method (Games-Howell Post-Hoc Test - unequal variances)

(I) Vegetal extract group	(J) Vegetal extract group	Mean Difference (I-J)	Std. Error	Sig.	95% Confidence Interval	
					Lower Bound	Upper Bound
ABTS Betulae	*ABTS Liquiritiae*	0.0010333	0.1179421	1.000	-0.323338	0.325404
	ABTS Avenae	.3240000*	0.0923033	0.031	0.036919	0.611081
ABTS Avenae	*ABTS Betulae*	-.3240000*	0.0923033	0.031	-0.611081	-0.036919
	ABTS Liquiritiae	-.3229667*	0.0809888	0.017	-0.571839	-0.074094
ABTS Liquiritiae	*ABTS Betulae*	-0.0010333	0.1179421	1.000	-0.325404	0.323338
	ABTS Avenae	.3229667*	0.0809888	0.017	0.074094	0.571839

Table S9. Multiple comparisons between groups for DPPH method (Games-Howell Post-Hoc Test - unequal variances)

(I) Vegetal extract group	(J) Vegetal extract group	Mean	Std. Error	Sig.	95% Confidence Interval

		Difference (I-J)			Lower Bound	Upper Bound
DPPH Betulae	*DPPH Liquiritiae*	.2258400*	0.0757520	0.026	0.026576	0.425104
	DPPH Avenae	.3842700*	0.0676078	0.001	0.195858	0.572682
DPPH Liquiritiae	*DPPH Betulae*	-.2258400*	0.0757520	0.026	-0.425104	-0.026576
	DPPH Avenae	.1584300*	0.0348389	0.003	0.061825	0.255035
DPPH Avenae	*DPPH Betulae*	-.3842700*	0.0676078	0.001	-0.572682	-0.195858
	DPPH Liquiritiae	-.1584300*	0.0348389	0.003	-0.255035	-0.061825

Table S10. Multiple comparisons between groups for FRAP method (Games-Howell Post-Hoc Test - unequal variances)

(I) Vegetal extract group	(J) Vegetal extract group	Mean Difference (I-J)	Std. Error	Sig.	95% Confidence Interval	
					Lower Bound	Upper Bound
FRAP Betulae	*FRAP Liquiritiae*	1.0506600*	0.1844807	0.000	0.546845	1.554475
	FRAP Avenae	.7373800*	0.1820244	0.006	0.236123	1.238637
FRAP Liquiritiae	*FRAP Betulae*	-1.0506600*	0.1844807	0.000	-1.554475	-0.546845
	FRAP Avenae	-.3132800*	0.0590746	0.000	-0.464930	-0.161630
FRAP Avenae	*FRAP Betulae*	-.7373800*	0.1820244	0.006	-1.238637	-0.236123
	FRAP Liquiritiae	.3132800*	0.0590746	0.000	0.161630	0.464930

S1.7. Molecular docking

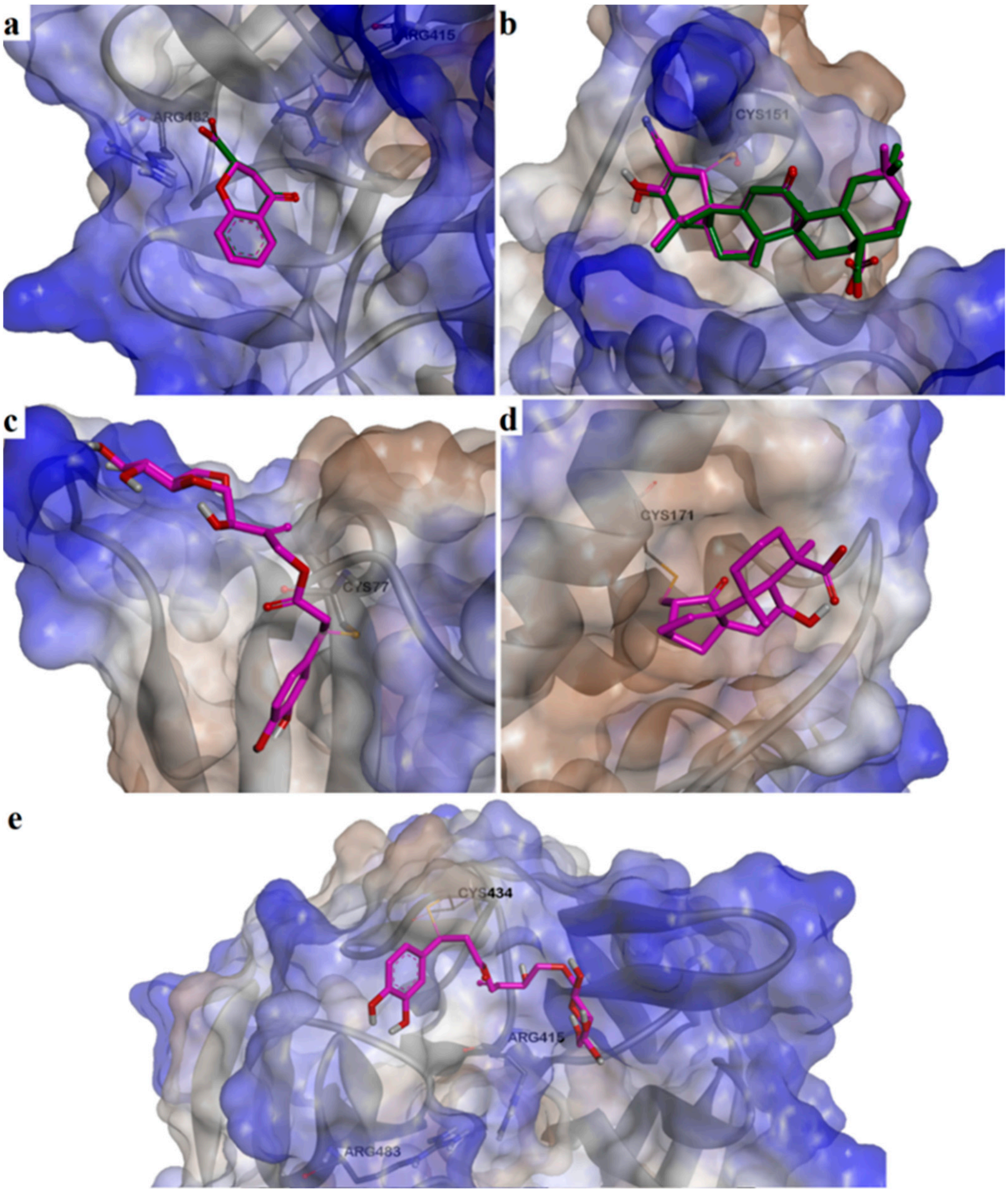

Figure S54. Binding poses of positive controls. (a) – superposition of predicted pose (pink) on initial conformation (green) for chromone-2-carboxylic acid (Kelch domain, noncovalent site); (b) – superposition of predicted pose (green) on initial conformation (pink) for bardoxolone (BTB domain, Cys151); (c) – predicted pose for pubescenoside A after binding to Cys77 (BTB domain); (d) – predicted pose for pterisolic acid B after binding to Cys171 (BTB domain); (e) – predicted pose for pubescenoside A after binding to Cys434 (Kelch domain); adjacent arginine residues that bind noncovalent ligands are also highlighted (Arg415, Arg483). Protein surface is colored by hydrophobicity, from blue (hydrophilic residues) to brown (hydrophobic residues).

Table S11. Molecular docking results after simulating noncovalent interactions between phytochemicals and Keap1 Kelch domain.

Ligand	ΔG (kcal/mol)	LE	Polar/electrostatic interactions
Aucubin	-7.014	0.292	5
Avenacoside A	-8.125	0.110	5
Betulin	-7.657	0.239	2
Betulinaldehyde	-7.827	0.245	0
Betulinic acid	-7.882	0.239	0
Betulonic acid	-8.119	0.246	0
Betuloside	-7.053	0.307	6
Caffeic acid	-6.158	0.474	5
Chlorogenic acid	-7.595	0.304	6
Cinnamic acid	-5.928	0.539	5
Dihydrocaffeic acid	-6.105	0.470	5
Ferulic acid	-6.343	0.453	5
Glabridin	-7.846	0.327	0
Glycyrrhizin	-10.112	0.174	8
Isoliquiritigenin	-7.592	0.400	4
Kaempferol	-7.608	0.362	3
Liquiritigenin	-7.226	0.380	4
Lupenone	-8.339	0.269	1
Lupeol	-8.483	0.274	3
Oleanolic acid	-8.642	0.262	0
p-Coumaric acid	-6.102	0.509	4
Quercetin	-7.538	0.343	2
Tricin	-7.597	0.317	6
Ursolic acid	-9.791	0.297	5
Vitexin	-9.014	0.291	4
Chromone-2-carboxylic acid*	-7.204	0.515	6

ΔG – binding energy; LE – ligand efficiency; * – positive control.

Table S12. Molecular docking results after simulating covalent interactions with reactive cysteines within Keap1 BTB and Kelch domains.

**Ligand**	**Cys77**		**Cys151**		**Cys171**		**Cys434**	
	**ΔG (kcal/mol)**	**LE**	**ΔG (kcal/mol)**	**LE**	**ΔG (kcal/mol)**	**LE**	**ΔG (kcal/mol)**	**LE**
Bardoxolone	3.700	-	-12.600*	0.350*	0.000	-	-8.390	0.233
Pterisolic acid B	-9.320	0.388	-12.150	0.506	-13.450*	0.560*	-11.840	0.493
Pubescenoside A	-17.790*	0.574*	-15.180	0.490	-17.780	0.574	-16.200*	0.523*
Isoliquiritigenin	-9.580	0.504	-11.560	0.608	-13.050	0.687	-12.020	0.633
Chlorogenic acid	-14.260	0.570	-14.640	0.586	-16.260	0.650	-14.140	0.566

ΔG – binding energy; LE – ligand efficiency; * – positive control.

S1.8. Radical scavenging activity

Table S13. The concentration range for *Betulae extractum*, *Liquiritiae extractum* and *Avenae extractum* used in the in vitro methods for antioxidant activity evaluation

**Method of antioxidant activity evaluation**	***Betulae extractum* concentration range**	***Liquiritiae extractum* concentration range**	***Avenae extractum* concentrantion range**
DPPH method	16.0 – 160.0 μg/mL	80.3 – 803.2 μg/mL	16.0 – 160.5 μg/mL
ABTS method	8.0 – 48.2 μg/mL	80.4 – 482.4 μg/mL	16.0 – 96.3 μg/mL
FRAP method	80.4 – 803.6 μg/mL	80.3 – 803.2 μg/mL	80.2 – 802.4 μg/mL
